# The First Phytochemical Investigation of *Artemisia divaricate*: Sesquiterpenes and Their Anti-Inflammatory Activity

**DOI:** 10.3390/molecules28104254

**Published:** 2023-05-22

**Authors:** Siqi Yan, Changqiang Ke, Zheling Feng, Chunping Tang, Yang Ye

**Affiliations:** 1 State Key Laboratory of Drug Research, Shanghai Institute of Materia Medica, Chinese Academy of Sciences, Shanghai 201203, China; s20-yansiqi@simm.ac.cn (S.Y.); kechangqiang@simm.ac.cn (C.K.); fengzheling@simm.ac.cn (Z.F.); 2 Natural Products Chemistry Department, Shanghai Institute of Materia Medica, Chinese Academy of Sciences, Shanghai 201203, China; 3 University of Chinese Academy of Sciences, No. 19A Yuquan Road, Beijing 100049, China; 4 School of Life Science and Technology, ShanghaiTech University, Shanghai 201203, China

**Keywords:** *Artemisia divaricate*, sesquiterpene, divaricanolide A–I, anti-inflammatory activity

## Abstract

*Artemisia divaricate* belongs to the *Artemisia* genus of the family of Compositae, a sort of perennial herb endemic in most regions of China. For the first time, a phytochemical investigation was carried out on the whole plant of *Artemisia divaricate*, resulting in the identification of 39 sesquiterpenes, with 9 of them being new (**1**–**9**). The structures of the new compounds were fully established using extensive analysis of MS and 1D and 2D NMR spectroscopic data and density functional theory (DFT) NMR calculations. Their structures involve germacrane, eudesmane, and bisabolane types. All the new isolates were evaluated for their anti-inflammatory activities in lipopolysaccharide (LPS)-stimulated murine macrophages of RAW 264.7 cells. Compounds **2** and **8** showed a significant inhibition effect on NO production, with IC_50_ values of 5.35 ± 0.75 and 7.68 ± 0.54 µM, respectively.

## 1. Introduction

Natural products, defined as components or metabolites of animals, plants, or microorganisms, play a highly important role in drug discovery and development due to their diversified structures and bioactivities [1]. Sesquiterpenes, which are an essential class of secondary metabolites, have a wide distribution in plants, especially those of the Asteraceae family [2]. The *Artemisia* genus, one of the largest genera of the Asteraceae family, possesses a unique position in traditional Chinese medicine, as a variety of *Artemisia* plants has a long history of being used as a medicine [3]. Extensively, phytochemical investigations on *Artemisia* plants have been launched for decades, yielding numerous sesquiterpenoid compounds, including mainly eudesmanolides, guaianolides, and germacranolides [4,5,6,7]. It is noteworthy that germacranolides exist as the largest group, which act as biogenetic precursors for the other types of sesquiterpene lactones. Previous investigations revealed the diversity of the pharmacological properties of germacranolides, such as antitumor [8], anti-inflammatory [9], antibacterial [10], cytotoxic [11], and immune [12] effects.

*Artemisia divaricate* (Pamp.) Pamp., a species endemic in China, is a perennial herbaceous plant mainly distributed in western Hubei, western Sichuan, and northern Yunnan [13]. So far, no phytochemical investigations have been reported on this plant. 

**Figure 1:**
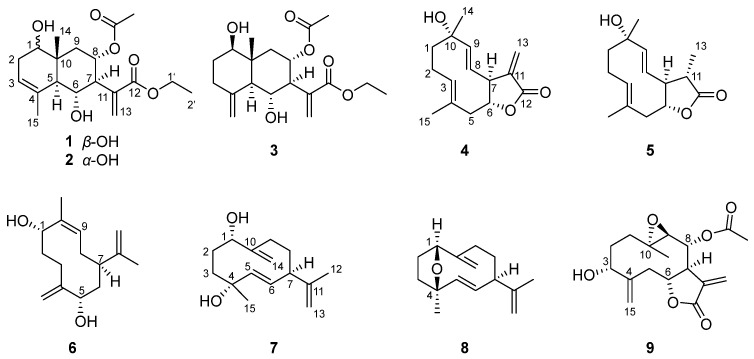
Structures of new compounds **1**–**9**.

In our continuous effort to search for bioactive constituents from natural sources, a systematic investigation of *A. divaricate* was performed, resulting in the isolation of 39 sesquiterpenes from the title plant for the first time. Herein, we describe the isolation and structural elucidation of nine undescribed sesquiterpenes of eudesmane or germacrane types (Figure 1; structures of known compounds, see Figure S1 in Supporting Information). Their structures were elucidated using extensive analysis of the spectroscopic data and DFT NMR calculations. All the new isolates were evaluated for their anti-inflammatory activities in lipopolysaccharide (LPS)-stimulated murine macrophages of RAW 264.7 cells, and two compounds (**2** and **8**) showed a significant inhibitory effect on NO production.

## 2. Results and Discussion

### 2.1. Structural Elucidation

**Figure 2:**
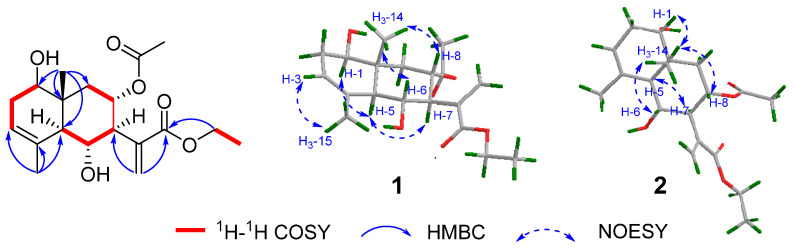
Key ^1^H–^1^H COSY, HMBC, and NOESY correlations of compounds **1** and **2**.

Compound **1**, the obtained white powder, had a molecular formula of C_19_H_28_O_6_, which was established using the HR-ESIMS ion at *m*/*z* 375.1858 [M + Na]^+^ (calcd. for C_19_H_28_O_6_Na, 375.1784), suggesting the presence of six degrees of unsaturation. The IR showed absorption bands for hydroxy (3440 cm^−1^) and carbonyl groups (1715 and 1626 cm^−1^). In the ^1^H and ^13^C NMR spectra of **1** (Table 1 and Table 2), signals for a 15-carbon skeleton, two substitution groups of an ethoxy group [*δ*_H_ 4.25 (2H, q), *δ*_C_ 61.2; *δ*_H_ 1.33 (3H, t), *δ*_C_ 14.3], and an acetoxy group [*δ*_H_ 1.95 (3H, s); *δ*_C_ 170.3, 21.2] were observed. The skeletal carbons were ascribed to a tertiary methyl [*δ*_H_ 0.94 (3H, s), *δ*_C_ 24.2], a vinylic methyl [*δ*_H_ 1.86 (3H, s), *δ*_C_ 11.5], three oxygenated methine [*δ*_H_ 3.61 (dd), *δ*_C_ 75.6; *δ*_H_ 4.03 (dd), *δ*_C_ 71.3; *δ*_H_ 5.41 (ddd), *δ*_C_ 70.0], trisubstituted double bonds [*δ*_H_ 5.34 (dd); *δ*_C_ 122.1, 134.5], an exocyclic double bond [*δ*_H_ 5.73 (s), 6.35 (s); *δ*_C_ 128.9, 138.6], and an ester carbonyl group (*δ*_C_ 167.0), which suggested an eudesmane-type structure of **1**. In the ^1^H–^1^H COSY spectrum, two fragments of H-1/H_2_-2/H-3 and H-5/H-6/H-7/H-8/H_2_-9 were established (Figure 2). The HMBC correlations from one methyl (*δ*_H_ 1.86) to C-3 (*δ*_C_ 122.1), C-4 (*δ*_C_ 134.5), and C-5 (*δ*_C_ 52.0) and from the other methyl (*δ*_H_ 0.94) to C-1 (*δ*_C_ 75.6), C-5 (*δ*_C_ 52.0), C-9 (*δ*_C_ 40.2), and C-10 (*δ*_C_ 38.9) further presented a nucleus moiety of two fused six-membered rings, with a hydroxyl group attached to C-1. The HMBC correlations from H_2_-13 (*δ*_H_ 6.35, 5,73) to C-7 (*δ*_C_ 57.2), C-11 (*δ*_C_ 138.6), and C-12 (*δ*_C_ 167.0) indicated an acrylic acid was placed at C-7 (Figure 2). The ethoxy group was confirmed to be a part of an ethyl ester group from the HMBC correlation between the oxygenated methylene and C-12 (*δ*_C_ 167.0). However, the location of the acetoxyl group remained unclear due to the absence of a key HMBC correlation relevant to H-8 (*δ*_H_ 5.41, ddd).

Based on the HR-ESIMS data, compound **2** (white powder) was designated a molecular formula of C_19_H_28_O_6_, the same as that of **1**. The IR showed absorption bands for hydroxy (3440 cm^−1^) and carbonyl groups (1715 and 1626 cm^−1^). A detailed NMR analysis of compound **2** (Table 1 and Table 2) showed high similarities between these two compounds, suggesting they might share the same planar structure. Detailed analysis of its ^1^H-^1^H COSY and HMBC data further supported such elucidation (Figure 2).

Given the acetoxyl group is likely to be attached to C-8 or C-6, there were two possible structures for compounds **1** and **2**, namely the 8-OAc and 6-OAc isomers (Figure S18). DFT NMR calculation was performed on the two isomers to figure out the most possible structure. A conformational search was conducted using Conflex in a 5.0 kcal/mol energy window [14]. All conformers were reoptimized at the B3LYP/6-31G(d) in vacuo, and their ^1^H and ^13^C NMR chemical shifts were calculated at the level of mPW1PW91/6-311G (d,p) with the PCM solvent mode for chloroform [15]. The improved statistical method DP4+ was used to analyze the calculated data of two possible structures and the experimental data [16]. The results of compound **1** gave a 100.00% (all data) possibility for the 8-OAc isomer, and compound **2** gave 97.20% (all data) for the same isomer (Figures S19 and S20), suggesting both are 8-OAc derivatives.

The relative configurations of **1** and **2** were determined using the coupling constant and NOESY cross-peaks. The *Z*-form of C-3 and C-4 was revealed using the NOESY correlation of H–3/H_3_–15 in both **1** and **2**. The correlations of H_3_-14/H-8/H-6 were observed in the NOESY spectrum of **1** and **2**, which supported that H_3_-14, H-8, and H-6 were on the same face and in *β*-orientation. The coupling constant of *J*_6-7_ (10.4 Hz for **1**, 10.5 Hz for **2**) indicated that these two protons in both compounds were in a trans form. The correlation of H-1/H-5 and H-5/H-7 was observed for **1**, suggesting that H-1, H-5, and H-7 were on the same face and in α-orientation. The correlations of H-5/H-7 and H_3_-14/H-1 were observed for **2**, suggesting that H-5 and H-7 were α-orientated, while H-1 was *β*-orientated (Figure 2). Thus, compound **2** was proposed as a C-1 epimer of **1**. Therefore, the full structures of compounds **1** and **2** were established as shown and named divaricanolides A and B.

Compound **3** was obtained as a colorless oil. Its molecular formula C_19_H_28_O_6_ was deduced from the HR-ESIMS data, indicative of six indices of hydrogen deficiency. However, in the ^13^C NMR and DEPT spectra, only 18 carbon resonances were well resolved. After a detailed analysis of its HSQC and HMBC data, two methine carbon signals (*δ*_C_ 54.80; *δ*_H_ 2.62, 1.83) were found to have overlapped.

**Figure 3:**
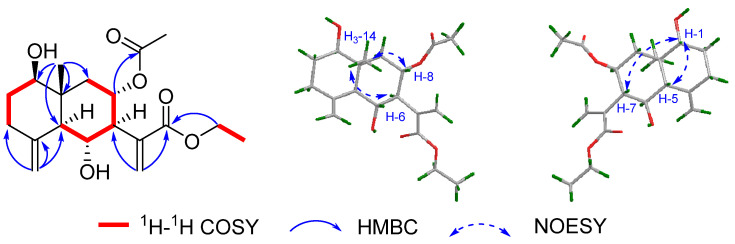
Key ^1^H–^1^H COSY correlations and HMBC and NOESY correlations of compound **3**.

A comparison of its NMR data (Table 1 and Table 2) with those of **1** showed that they possessed the same skeleton, having an acetoxyl (*δ*_H_ 1.95; *δ*_C_ 170.3, 21.2) located at C-8, with an ethyl ester group located at C-13. The diagnostic signals for a terminal double bond (*δ*_H_ 5.03, 4.76; *δ*_C_ 109.1, 144.6) were observed for **3**, which were located at C-4 and C-15 using HMBC correlation (Figure 3) from H_2_-15(*δ*_H_ 5.03 and 4.76) to C-3 (*δ*_C_ 34.9), C-4 (*δ*_C_ 144.6), and C-5 (*δ*_C_ 54.8). Analysis of the coupling constants and NOESY data established the relative stereochemistry of **3**. The NOESY correlations of H_3_-14/H-6 and H_3_-14/H-8 supported *β*-orientations of Me-14, H-6, and H-8, and *α*-orientations of the hydroxy group at C-6 and the acetoxy group at C-8. The coupling constant of *J*_6-7_ (10.4 Hz) indicated a trans form of H-6 and H-7. The *β*-orientations of H-5 and H-1 were proved using the NOE correlations H-1/H-5 and H-1/H-7 (Figure 3). Therefore, the structure of **3** was proposed and named divaricanolide C.

Compound **4**, an colorless oil, had a molecular formula of C_15_H_20_O_3_, which was established based on the HR-ESIMS ion at *m*/*z* 249.1507 [M + H]^+^ (calcd. for C_15_H_21_O_3_, 249.1491), suggesting the presence of six degrees of unsaturation. The IR showed absorption bands for hydroxy (3447 cm^−1^) and carbonyl groups (1763 cm^−1^). The ^13^C NMR spectrum of **4** (Table 1) displayed one carbonyl carbon at *δ*_C_ 170.6 and six olefinic carbons at *δ*_C_ 147.1, 139.6, 132.5, 127.2, 123.4, and 121.1. The ^1^H NMR spectrum (Table 2) exhibited two methyl groups at *δ*_H_ 1.39 (s) and 1.50 (overlapped) and five olefinic protons at *δ*_H_ 6.17 (d), 5.71 (d), 5.46 (d), 5.18 (dd), and 5.15 (dd). The above spectroscopic data accounted for seven degrees of unsaturation, and the remaining two degrees of unsaturation were represented by a bicyclic carbon skeleton in compound **4**.

**Figure 4:**
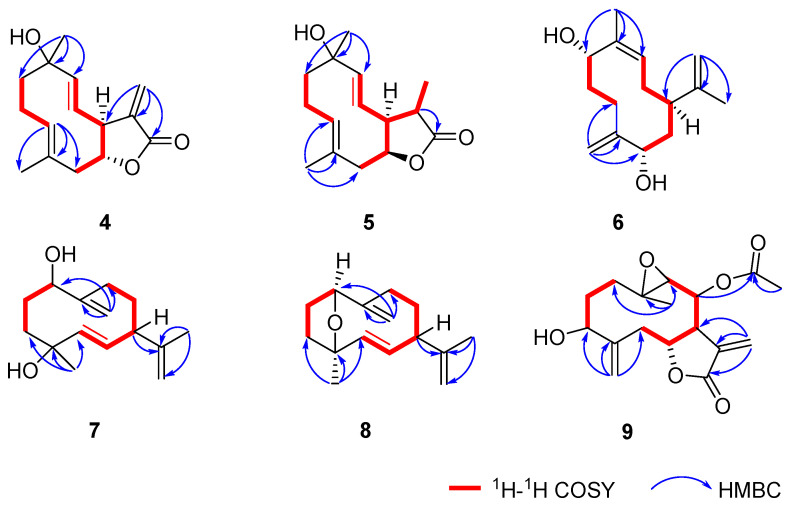
Key ^1^H–^1^H COSY and HMBC correlations of compounds **4**–**9**.

In the ^1^H–^1^H COSY spectrum, two fragments were established from the correlations of H_2_-5/H-6/H-7/H-8/H-9 and H_2_-1/H_2_-2/H-3 (Figure 4). Further HMBC correlation (Figure 4) from H-3 (*δ*_H_ 5.18, dd) to C-4 (*δ*_C_ 127.2)/C-5 (*δ*_C_ 45.4)/C-15(17.8) and from H_3_-14 (*δ*_H_ 1.39, s) to C-1 (*δ*_C_ 41.2)/C-9(*δ*_C_ 147.1)/C-10 (*δ*_C_ 73.0) indicated the occurrence of a 10-membered ring with two methyl groups located at C-4 and C-10, respectively, and a hydroxy group at C-10. The presence of a fused unsaturated lactone ring at C-6 and C-7 was established using the HMBC correlations from H_2_-13(*δ*_H_ 6.17, d; 5.46, d) to C-7 (*δ*_C_ 55.8), C-11 (*δ*_C_ 139.6), and C-12 (*δ*_C_ 170.6). Consequently, compound **4** was elucidated as a germacrane-type sesquiterpene. 

**Figure 5:**
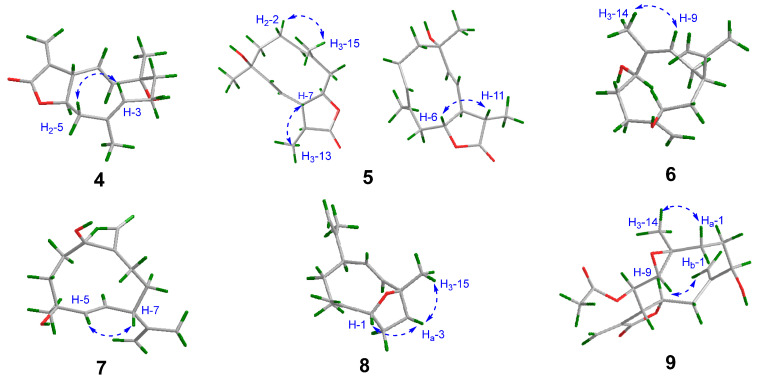
NOESY correlations (dashed arrow) of compounds **4**–**9**.

The NOESY correlations between H-3/H-5 and the coupling constant of *J*_8-9_ (16.1 Hz) inferred *E*-geometry for both C-3/C-4 and C-8/C-9 double bonds (Figure 5). Due to the fact that the *α*-orientation of H-7 was reported for the majority of the naturally occurring germacrane-type sesquiterpenes, H-7 in compound **4** was tentatively defined as *α*-orientation. The coupling constant of *J*_6-7_ (9.8 Hz) indicated that these two protons were in a trans form; namely, H-6 was *β*-orientated. The relative configuration of the OH-10 remained unclear. To further elucidate the relative configuration, the same DFT NMR calculation method used to describe compounds **1** and **2** was performed on two possible isomers with *rel*-(6*R*,7S,10*R*) and *rel*-(6*R*,7S,10*S*) configurations (Figure S37). The DP4+ statistical analysis showed a 100% DP4+ probability (all data) for the *rel*-(6*R*,7S,10*S*) isomer (Figure S38). Therefore, the structure of **4** was proposed as shown and named divaricanolide D.

Compound **5**, a colorless oil, had a molecular formula of C_15_H_22_O_3_ established using HR-ESIMS. Its NMR data (Table 1 and Table 2) were similar to those of compound **4**, suggesting that **5** might be an analogue of **4**. A comparison of their ^1^H and ^13^C NMR data revealed that an additional methyl signal (*δ*_C_ 12.6 and *δ*_H_ 1.19) was observed in **5**, accompanied by the absence of an exocyclic double bond when compared with **4**. The position of the additional methyl group was designated as Me-13 using the ^1^H–^1^H COSY correlations (Figure 4) of H-7 (*δ*_H_ 2.62)/H-11 (*δ*_H_ 2.43)/H_3_-13 (*δ*_H_ 1.19). 

The NOESY correlations between H_2_-2/H_3_-15 and the coupling constant of *J*_8-9_ (16.0 Hz) revealed *E*-geometry for both the C-3/C-4 and C-8/C-9 double bonds. The coupling constant of *J*_6-7_ (12.1 Hz) indicated that these two protons kept in a trans form. The correlation of H_3_-13/H-7 and H-11/H-6 was observed in the NOESY spectrum, which supported that H_3_-13, and H-7 were in *α*-orientation, while H-6 and H-11 were in *β*-orientation (Figure 5). Similar to compound **4**, the relative configuration of the hydroxyl group at C-10 was unclear. The same DFT NMR calculation was performed on two possible isomers, *rel*-(6*R*,7S,10*R*,11*S*)-**5** and (6*R*,7S,10*S*,11*S*)-**5** (Figure S47). The DP4+ analysis result provided a 100% probability (all data) for the (6*R*,7S,10*S*,11*S*)-**5** (Figure S48). Accordingly, the full structure of **5** was proposed and named divaricanolide E.

Compound **6**, a colorless oil, had a molecular formula of C_15_H_24_O_2_ established using HR-ESIMS, implying four degrees of unsaturation. Analysis of the ^1^H and ^13^C NMR data of **6** (Table 1 and Table 3) revealed the existence of three double bonds, including one trisubstituted [*δ*_C_ 128.4, *δ*_H_ 5.25 (d); *δ*_C_ 136.0], two exocyclic [*δ*_C_ 113.0, *δ*_H_ 5.17 (s), 5.01 (s); *δ*_C_ 150.0; *δ*_C_ 110.5, *δ*_H_ 4.89 (s), 4.64 (s); *δ*_C_ 147.1], and two oxymethines [*δ*_C_ 74.6, *δ*_H_ 4.10 (dd); *δ*_C_ 67.7, *δ*_H_ 4.91 (dd)]. The remaining seven carbons were assigned to two methyls, four methylenes, and one methine. With one remaining unsaturation degree, **6** should be monocyclic. 

The ^1^H-^1^H COSY spectrum of compound **6** proved to be very informative, as only two spin systems were detected, H-1/H_2_-2/H-3 and H-5 /H_2_-6/H-7/H_2_-8/H-9 (Figure 4). The HMBC correlations from H_2_-15 (*δ*_H_ 5.17, 5.07) to C-3 (*δ*_C_ 22.7), C-4 (*δ*_C_ 150.0), and C-5 (*δ*_C_ 74.6) and H_3_-14 (1.74) to C-1 (67.7), C-9 (128.4), and C-10 (136.0) eventually merged two fragments into a 10-membered carbocycle typical of the germacrane skeleton, with two hydroxy groups located at C-1 and C-5, respectively (Figure 4). The 2-methyl-1-ene isopropyl group was placed at C-7 using the HMBC correlations from H_3_-12 (*δ*_H_ 1.85) to C-7 (*δ*_C_ 42.2), C-11 (*δ*_C_ 147.1), and C-13 (*δ*_C_ 110.5). 

The NOESY correlations between H_3_-14/H-9 inferred *Z*-geometry for the C-9/C-10 double bond (Figure 5). Same as compound **4**, the relative configuration of H-7 was tentatively defined as *α*-orientation. Thus, the relative configurations of H-1 and H-5 still remained unclear. Then, DFT NMR calculation was performed on four possible isomers, with relative configurations of *rel*-(1*R*,5*R*,7*R*), *rel*-(1*S*,5*R*,7*R*), *rel*-(1*S*,5*R*,7*R*), and *rel*-(1*S*,5*S*,7*R*) (Figure S57). The DP4+ probability finally supported the *rel*-(1*S*,5*S*,7*R*) configuration with a 100% possibility for all data (Figure S58). Thus, the structure of **6** was proposed and named divaricanolide F.

Compound **7**, a colorless oil, was given a molecular formula of C_15_H_24_O_2_ using HR-ESIMS, corresponding to four degrees of unsaturation. Its NMR data (Table 1 and Table 3) were similar to those of compound **6** except that one disubstituted double bond [*δ*_C_ 138.0, *δ*_H_ 5.51(d); *δ*_C_ 130.9, *δ*_H_ 5.10(dd)] and one oxygenated quaternary carbon (*δ*_C_ 74.0) were observed in **7**, taking the place of the exocyclic double bond and the oxygenated methine in **6**. In the ^1^H-^1^H COSY spectrum of compound **7** (Figure 4), the correlations of H-5/H-6/H-7/H_2_-8/H_2_-9 proved the double bond located at C-5 and C-6. The hydroxyl group was placed at C-4, deduced from the HMBC correlations from H_3_-15 (*δ*_H_ 1.30) to C-3 (*δ*_C_ 38.0), C-4 (*δ*_C_ 74.0), and C-5 (*δ*_C_ 138.0) (Figure 4). The remaining exocyclic double bond was placed at C-10/C-14 using the HMBC correlations from H_2_-14 (*δ*_H_ 5.16, 4.99) to C-1 (*δ*_C_ 76.2), C-10 (*δ*_C_ 150.3), and C-9 (*δ*_C_ 30.5).

The NOESY correlations of H-5/H-7 and the coupling constant of *J*_5-6_ (16.0 Hz) all suggested *E*-geometry for the C-5/C-6 double bond (Figure 5 and Table 3). When H-7 was tentatively assigned as *α*-orientated, the relative configurations of C-1 and C-4 remained unclear. Similarly, DFT NMR calculation was conducted on four possible isomers with relative configurations of *rel*-(1*R*,4*S*,7*R*), *rel*-(1*R*,4*R*,7*R*), *rel*-(1*S*,4*S*,7*R*), and *rel*-(1*S*,4*R*,7*R*) (Figure S67). The DP4+ probability analysis gave a 100% possibility (all data) for the *rel*-(1*S*,4*R*,7*R*) configuration (Figure S68). Therefore, the structure of **7** was proposed and named divaricanolide G. 

Compound **8** had a molecular formula of C_15_H_22_O established using HR-ESIMS, corresponding to five indices of hydrogen deficiency. The IR absorption at 3450 was assigned to the hydroxyl group. The ^13^C NMR spectrum of **8** (Table 1) was very similar to that of **7**, suggesting they shared the same germacrane skeleton with similar functional groups. As compound **8** had one more degree of unsaturation than **7**, compound **8** might possess one more ring in the molecule. Given the existence of one nonproton-bearing oxygenated carbon at *δ*_C_ 73.7 in **8** and less H_2_O in the molecular formula when compared with **7**, an oxygen bridge between C-1 and C-4 was constructed, which was consistent with the low-field resonance of H-1 (*δ*_H_ 4.11, dd). Thus, the planar structure of **8** was established. 

The C-5/C-6 double bond was given *E*-geometry using the NOESY correlations of H-5/H-7 and the coupling constant of *J*_5-6_ (16.0 Hz) (Figure 5 and Table 3). The presence of the NOESY correlations of H-1/H_a_-3 and H_a_-3/H_3_–15 implied that H-1 and H_3_–15 were on the same face. However, the relationship between H-1/H_3_-15 and H-7 was uncertain. Similarly, DFT NMR calculation was performed on two possible isomers with relative configurations of *rel*-(1*R*,4*S*,7*R*) and *rel*-(1*S*,4*R*,7*R*) (Figure S77). The *rel*-(1*R*,4*S*,7*R*) configuration was finally designated for **8** using DP4+ probability with a 100% possibility for all data (Figure S78). Thus, the structure of **8** was proposed and named divaricanolide H.

Compound **9** was obtained as a colorless oil and possessed a molecular formula of C_17_H_22_O_6_ using HR-ESIMS in coincidence with seven degrees of unsaturation. The ^1^H and ^13^C NMR (Table 1 and Table 3) data of **9** exhibited characteristic signals for an acetoxyl [*δ*_H_ 2.21 (s, 3H); *δ*_C_ 173.1, 20.6], two exocyclic methylenes [*δ*_C_ 118.2, *δ*_H_ 5.39 (d), 5.16 (d); *δ*_C_ 144.5; *δ*_C_ 120.6, *δ*_H_ 6.18 (d), 5.40 (d); *δ*_C_ 138.7], four oxygenated methines [*δ*_C_ 81.8, *δ*_H_ 4.16 (ddd); *δ*_C_ 77.7, *δ*_H_ 4.47 (d); *δ*_C_ 77.1, *δ*_H_ 3.00 (d); *δ*_C_ 73.9, *δ*_H_ 5.32 (dd)], and one quaternary carbon [*δ*_C_ 69.8]. As the identified functional groups satisfied four degrees of unsaturation, the three left were indicative of a tricyclic ring system. The ^1^H–^1^H COSY spectrum revealed two spin systems of H_2_-1/H_2_–2/H-3 and H_2_-5/H-6/H-7/H-8/H–9 (Figure 4). The *γ*-lactone ring was deduced to be fused to C-6 and C-7 from the key HMBC correlations from H_2_-13 (*δ*_H_ 6.18, d; 5.40, d) to C-7 (*δ*_C_ 49.7)/C-11(*δ*_C_ 138.7)/C-12 (*δ*_C_ 168.8). The HMBC correlations (Figure 4) from H_3_–14 (*δ*_H_ 1.23, s) to C-1 (*δ*_C_ 37.3) /C-9 (*δ*_C_ 77.1)/C-10 (*δ*_C_ 69.8) and from H_2_-15 (*δ*_H_ 5.39, d; 5.16, d) to C-3 (*δ*_C_ 77.7)/C-4 (*δ*_C_ 144.5)/C-5 (*δ*_C_ 41.9) further constructed a germacrane-type sesquiterpenoid lactone. A hydroxyl group was assigned to C-3 (*δ*_C_ 77.7) according to its low-field chemical shift. An acetoxyl group was fixed at C-8 based on the HMBC correlations from H-8 (*δ*_H_ 5.32, dd) to a carboxylic carbon (*δ*_C_ 173.0). Given the one degree of unsaturation left and the molecular formula, an epoxide ring might be present in **9**. The low-field resonances of H-9 (*δ*_H_ 3.00) and C-10 (*δ*_C_ 69.8) verified that the ether linkage was at C-9/C-10. 

The NOESY correlations between H_a_-1/H_3_-14 and H_b_-1/H-9 revealed that H_3_-14 and H-9 were in a trans form (Figure 5). The coupling constant of *J*_6-7_ (9.2 Hz) also indicated a trans form for H-6 and H-7 (Table 3). Still, there were no evidence to clarify the relative configurations of **9** due to the four isolated chiral centers, C-3, C-6/C-7, C-8, and C-9/C-10. Given the tentative *α*-orientation for H-7, the left three chiral centers generated eight possible isomers, *rel*-(3*S*,6*R*,7*S*,8*R*,9S,10S), *rel*-(3*R*,6*R*,7*S*,8*R,*9S,10*S*), *rel*-(3*S*,6*R*,7*S*,8*S*,9*S*,10*S*), *rel*-(3*R*,6*R*,7*S*,8*S*,9*S*,10*S*), *rel*-(3*S*,6*R*,7*S*,8*R*,9*R*,10*R*), *rel*-(3*R*,6*R*,7*S*,8*R*,9*R*,10*R*), *rel*-(3*S*,6*R*,7*S*,8*S*, 9*R*,10*R*), and *rel*-(3*R*,6*R*,7*S*,8*S*,9*R*,10*R*) (Figure S87). DFT NMR calculation was conducted on the eight possible isomers, and the DP4+ probability analysis finally gave a 98.51% possibility (all data) to support the *rel*-(3*R*,6*R*,7*S*,8*R,*9S,10*S*) configuration (Figure S88). Thus, the structure of **9** was proposed and named divaricanolide I.

Apart from compounds **1**–**9**, 30 other known components were isolated and identified as austroyunnane H (**10**) [17], baynol C (**11**) [18], jatrophaeudesmene C (**12**) [19], dihydro-β-cyclopyrethrosin (**13**) [20], *β*-Cyclopyrethrosin (**14**) [21], yomogin (**15**) [22], (11*S*)-1*β*-Hydroxyeudes-m-4(14)-eno-13,6*α*-lactone (**16**) [23], reynosin (**17**) [24], 1*β*,8*β*-Dihydroxy-reynosin (**18**) [25], artemorin (**19**) [26], 1-epi-Dihydrochrysanolide (**20**) [27], dihydrochrysanolide (**21**) [27], 5*β*,6*α*-Hydroxygermacra-1(10)*E*,4(15),11(13)-trien-12,8*α*-olide (**22**) [28], tulirinol (**23**) [29], chamissarin (**24**) [19], 6*β*-Acetoxy-14-hydroxygermacra-4*E*,1(10)*E*,11(13)-trien-12,8*α*-olide (**25**) [30], 6*β*-Acetoxy-3-formyl-3*Z*,9*E*,11(13)-trien-12,8*α*-olide (**26**) [31], chrysanolide (**27**) [22], haagenolide (**28**) [32], 14-hydroxygermacra-4*E*,1(10)*E*, 11(13)-trien-12,7*α*-olide (**29**) [30], Chihuahuin (**30**) [29], austroliolide (**31**) [29], pyrethrosin (**32**) [29], 9*α*,10*β*-epoxi-8*α*-Hydrox-germacra-3*Z*,11(13)-dien-6*α*,12-olide (**33**) [33], sinugibberodiol (**34**) [34], (3*R*,7*S*,9*S*)-3,9-Dihydroxygermacra-4(15),10(14),11(12)-triene (**35**) [35], (3*S*,7*S*,9*S*)-3,9-dihydroxygermac-ra-4(15),10(14),11(12)-triene (**36**) [34], germacra-1(10)*E*,4*E*-dien-2*β*,6*β*-diol (**37**) [36], 4*β*,5*α*-Dihydroxycubenol (**38**) [37], and amarantholidoside III (**39**) [38] by comparing their spectroscope data with those reported in the literature.

### 2.2. Anti-Inflammatory Activity Assay 

**Figure 6:**
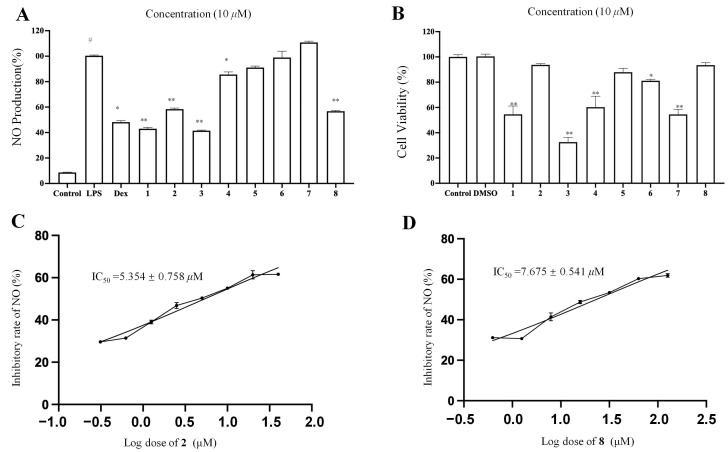
Cytotoxic and NO production inhibitory effects of compounds isolated from *A. divaricate.* (**A**) Inhibitory rate of NO production. (**B**) The cell viability of RAW 264.7 cells treated with different compounds at concentration of 10 μM. (**C**) Inhibitory rate of NO production for compounds **2** in LPS-induced RAW 264.7 cells. (**D**) Inhibitory rate of NO production for compounds **8** in LPS-induced RAW 264.7 cells. Data are shown as mean ± SEM. ^#^ *p* < 0.001 LPS vs. vehicle control. * *p* < 0.01 and ** *p* < 0.001 compounds or dexamethasone vs. LPS.

Macrophage inflammation plays a vital role in metabolic diseases, neurodegenerative diseases, and cancers [39]. The current research suggests that inflammation involves a lot of pro-inflammatory cytokines. NO is an important inflammatory mediator in inflammation. Natural products have been considered important sources to identify anti-inflammatory agents [40]. Herein, eight new compounds (**1**−**8**) were tested for their inhibitory effects on NO production in LPS-stimulated RAW 264.7 macrophages for a preliminary evaluation of their anti-inflammatory activity (Figure 6). Firstly, the noncytotoxic concentrations of compounds **1**–**8** were evaluated using an MTT [3-(4,5-dimethylthiazol-2-yl)-2,5-diphenyltetrazolium bromide] assay. The MTT results showed that compounds **2**, **5**–**6**, and **8** did not show evident cytotoxicity up to 10 µM (Figure 6B). Among these four compounds, only compounds **2** and **8** showed a significant inhibitory effect on the release of nitric oxide (NO) from RAW 264.7 cells (Figure 6A). Next, the IC_50_ values of compounds **2** and **8** in inhibiting NO production were evaluated, which were 5.35 ± 0.75 and 7.68 ± 0.54 µM, respectively. 

## 3. Materials and Methods

### 3.1. General Experimental Procedures 

Optical rotations were recorded on a Rudolph Research Analytical Autopol VI 90079 polarimeter (Hackettstown, NJ, USA). ECD spectra were recorded using a J-815 CD spectropolarimeter (JASCO, Tokyo, Japan). IR spectra were recorded on a Nicolet FTIR IS5 spectrometer (Thermo Fisher, Walthan, MA, USA). HR-ESIMS spectra were measured on a Waters Synapt G2-Si Q-TOF instrument and Agilent G6520 Q-TOF. NMR spectra were obtained on a Bruker AVANCE III 500 or 600 MHz spectrometer (Bruker Biospin AG, Frankenstein, Switzerland). Analytical HPLC was performed on a Waters e2695 system equipped with a Waters 2998 photodiode array detector (PDA), a Waters 2424 evaporative light-scattering detector (ELSD), and a Waters 3100 MS detector. Preparative HPLC was run on a Waters system equipped with a Waters 2767 autosampler, a Waters 2545 pump, a Waters 2489 PDA, and an Acuity ELSD using a Waters Sunfire RP C18 column (5 μm, 30 × 150 mm, flow rate 30 mL/min). Sephadex LH-20 (Pharmacia Biotech AB, Uppsala, Sweden), MCI gel CHP20P (75–150 μm, Mitsubishi Chemical Industries, Tokyo, Japan), ODS gel AAG12S50 (12 nm, S-50 μm, YMC Co., Ltd., Tokyo, Japan), and silica gel (300–400 mesh, Qingdao Marine Chemical Inc., Qingdao, China) were used for column chromatography (CC). TLC analyses were performed on prefabricated GF_254_ silica gel plates (Yantai Jiangyou silica gel development Co., Ltd., Yantai, China). All solvents were of analytical grade (Sinopharm Chemical Reagents Co., Ltd., Shanghai, China) for CC and of HPLC grade (Merck KGaA, Darmstadt, Germany) for HPLC and preparative HPLC.

### 3.2. Plant Material

The whole plant of *A. divaricate* was collected from Ganzi Area of Sichuan Province, China, in August 2020. The plant material was authenticated by Mr. Zhang Jun from Kunming Zhifen Biotechnology Co., Ltd., Kunming, China. A voucher specimen (No. 2018090606) was deposited in the herbarium of Shanghai Institute of Materia Medica, Chinese Academy of Sciences.

### 3.3. Extraction and Isolation 

The dried whole plant of *A. divaricate* (30.0 kg) was ground and extracted with 75 L 95% EtOH (7 days × 3) at room temperature. After removing the solvent, a crude residue (1.29 kg) was obtained, which was suspended in water and extracted successively with petroleum ether (PE), dichloromethane (DCM), and ethyl acetate (EA), affording fractions of PE (447.5 g), DCM (227.8 g), and EA (125.9 g), respectively. The DCM part was subjected to an AB-8 macroporous resin column eluted with aqueous EtOH in a step manner (20, 40, 60, 80, and 95%), affording five fractions: Frs. 1–5. Fr.2 (55.3 g) and Fr.3 (53.5 g) were combined and chromatographed over an MCI column eluted with aqueous MeOH (20, 30, 40, 50, 60, 70, 80, and 90%) to give Frs. 2A–2K. Fr. 2G (15.6 g) was subjected to an ODS gel column eluted with gradient elution of aqueous MeOH (30, 40, 50, 60, 70, and 80%) to afford subfractions of Frs. 2G1–2G10. Fr. 2G4 (4.0 g) was subjected to column chromatograph over Sephadex LH-20 gel (MeOH) to provide Frs. 2G4A–2G4F. Fr. 2G4B was subjected to silica gel CC (300–400 mesh) with gradient elution of DCM/MeOH (70:1–15:1) and then purified by preparative HPLC using CH_3_CN/H_2_O (0–35 min, from 21% to 41%) to give compound **3** (3.7 mg). Fr. 2G4C was separated using silica gel CC (300–400 mesh) with gradient elution of PE/acetone (15:1–2:1) and then preparative HPLC (19% to 39% CH_3_CN in H_2_O, 35 min) to yield compound **1** (1.8 mg) and compound **2** (2.5 mg). Fr. 2G6 (680.0 mg) was chromatographed over silica gel eluted with DCM-MeOH (40:1–20:1) and refined further using preparative HPLC (25–45% CH_3_CN in H_2_O, 35 min) to obtain compound **5** (18.1 mg). Fr. 2G7 (2.1 g) was subjected to CC over Sephadex LH-20 gel (MeOH) to afford Frs. 2G7A–2G7D. Fr. 2G7B was separated using CC over silica gel (300–400 mesh) with a gradient of DCM-EA (8:1–1:1) and then preparative HPLC (15–35% CH_3_CN in H_2_O, 35 min) to yield compound **8** (3.4 mg). Fr. 2G7C was separated using CC over silica gel (300–400 mesh) using a gradient of DCM-MEOH (40:1–20:1) and then preparative HPLC (18–38% CH_3_CN in H_2_O, 35 min) to yield compound **4** (2.0 mg). Fr. 2G7E was separated using CC over silica gel (300–400 mesh) with a gradient of PE–acetone (6:1–2:1) and then preparative HPLC (25–45% CH_3_CN in H_2_O, 35 min) to yield compound **9** (0.8 mg). Frs. 2G8C and 2G8D were separated using a silica gel column (300–400 mesh) with a gradient of PE:acetone (10:1–2:1) to yield compounds **6** (3.9 mg) and **7** (7.8 mg), respectively.

Divaricanolide A (**1**): white powder; ${\left[\alpha \right]}_{D}^{20}$ − 36 (*c* 0.1, MeOH); ECD (MeOH) (*Δε*) 195 (+3.8) nm, 191.5 (−3.8) nm; IR(KBr) *ν*_max_ 3440, 2921, 2852, 1715, 1626, 1440, 1371, 1324, 1240, 1176, 1022, 962 cm^−1^; ^1^H and ^13^C NMR, see Table 1 and Table 2; HR-ESIMS *m*/*z* 375.1858 [M + Na]^+^ (calcd. for C_19_H_28_O_6_Na, 375.1784).

Divaricanolide B (**2**): white powder; ${\left[\alpha \right]}_{D}^{20}$ + 60 (*c* 0.1, MeOH); ECD (MeOH) (*Δε*) 208.8 (+1.7) nm, 203 (−3.1) nm, 201.5 (+6.0) nm, 197 (−5.4) nm, 195 (+2.1) nm, 191.5 (−4.9) nm, 190 (+4.6) nm; IR(KBr) *ν*_max_ 3440, 2921, 2852, 1715, 1626, 1440, 1371, 1240, 1176, 1022 cm^−1^; ^1^H and ^13^C NMR data, see Table 1 and Table 2; HR-ESIMS *m*/*z* 375.1874 [M + Na]^+^ (calcd. for C_19_H_28_O_6_Na, 375.1784).

Divaricanolide C (**3**): colorless oil; ${\left[\alpha \right]}_{D}^{20}$ − 48 (*c* 0.1, MeOH); ECD (MeOH) (*Δε*) 192 (−12.2) nm; IR(KBr) ν_max_ 3421, 2924, 2852, 1663, 1432, 1382, 1213, 1116, 1060 cm^−1^; ^1^H and ^13^C NMR data, see Table 1 and Table 2; HR-ESIMS *m*/*z* 375.1858 [M + Na]^+^ (calcd. for C_19_H_28_O_6_Na, 375.1784).

Divaricanolide D (**4**): colorless oil; ${\left[\alpha \right]}_{D}^{20}$ − 28 (*c* 0.1, MeOH); ECD (MeOH) (*Δε*) 203.5 (−12.2) nm; IR(KBr) *ν*_max_ 3447, 2920, 1763, 1384, 1144 cm^−1^; ^1^H and ^13^C NMR data, see Table 1 and Table 2; HR-ESIMS *m*/*z* 249.1507 [M + H ]^+^ (calcd. for C_15_H_21_O_4_, 249.1491).

Divaricanolide E (**5**): colorless oil; ${\left[\alpha \right]}_{D}^{20}$ − 88 (*c* 0.1, MeOH); ECD (MeOH) (*Δε*) 228.5 (−9.4) nm, 195.5 (+0.6) nm; IR(KBr) *ν*_max_ 3447, 2924, 1773, 1383, 1185, 1109, 994 cm^−1^; ^1^H and ^13^C NMR data, see Table 1 and Table 2; HR-ESIMS *m*/*z* 273.1463 [M + Na]^+^ (calcd. for C_15_H_22_O_3_Na, 273.1464).

Divaricanolide F (**6**): colorless oil; ${\left[\alpha \right]}_{D}^{20}$ + 68 (*c* 0.1, MeOH); ECD (MeOH) (*Δε*) 197 (−8.2) nm; IR(KBr) *ν*_max_ 3355, 2927, 1642, 1449, 1376, 1015, 889 cm^−1^; ^1^H and ^13^C NMR data, see Table 1 and Table 3; HR-ESIMS *m*/*z* 219.1744 [M + H − H_2_O]^+^ (calcd. for C_15_H_23_O, 219.1749).

Divaricanolide G (**7**): colorless oil; ${\left[\alpha \right]}_{D}^{20}$ + 68 (*c* 0.1, MeOH); ECD (MeOH) (*Δε*) 200 (−19.8) nm, 220.5 (+1.5) nm; IR(KBr) *ν*_max_ 3385, 3079, 2927, 1644, 1449, 1373, 1078, 1032, 977, 891 cm^−1^; ^1^H and ^13^C NMR data, see Table 1 and Table 3; HR-ESIMS *m*/*z* 219.1744 [M + H − H_2_O]^+^ (calcd. for C_15_H_23_O, 219.1749).

Divaricanolide H (**8**): colorless oil; ${\left[\alpha \right]}_{D}^{20}$ − 91 (*c* 0.1, MeOH); ECD (MeOH) (*Δε*) 191.5 (+10.8) nm, 198 (−14.8) nm; IR(KBr) *ν*_max_ 3373, 2926, 2854, 1644, 1449, 1372, 1118, 1078, 1032, 977, 950, 891 cm^−1^; ^1^H and ^13^C NMR data, see Table 1 and Table 3; HR-ESIMS *m*/*z* 219.1744 [M + H]^+^ (calcd. for C_15_H_23_O, 219.1749).

Divaricanolide I (**9**): colorless oil; ${\left[\alpha \right]}_{D}^{20}$ + 2.8 (*c* 0.1, MeOH); ECD (MeOH) (*Δε*) 213.5 (+4.6) nm, 194.5 (−4.2) nm; IR(KBr) *ν*_max_ 2925, 1772, 1716, 1373, 1260 1145, 1077, 1041, 1026, 1002, 929, 959 cm^−1^; ^1^H and ^13^C NMR data, see Table 1 and Table 3; HR-ESIMS *m*/*z* 323.1490 [M + H]^+^ (calcd. for C_17_H_23_O_6_, 323.1489).

### 3.4. Computational Section

DFT NMR was performed using the Gaussian 16 program [14]. Conformational searching was conducted using Conflex 8.0 software with the MMFF force field within an energy window of 5.0 kcal/mol [14]. Conformers with the Boltzmann population above 0.1% were reoptimized at the B3LYP/6- 311G(d) level in vacuo, and then their NMR data were calculated at the level of mPW1PW91/6-311G(d, p) with the PCM solvent mode for chloroform [15]. A possible configuration was specified using DP4+ probability [16] 

### 3.5. Cell Culture

RAW 264.7 macrophages were purchased from American Type Cell Collection (Manassas, VA, USA) and cultured in Dulbecco’s Modified Eagle Medium (DMEM) containing 10% fetal bovine serum (Gibco, Carlsbad, CA, USA) and 1% penicillin–streptomycin (Gibco, Carlsbad, CA, USA) in a humidified incubator with 5% CO_2_ at 37 °C.

### 3.6. Cell Viability

The cell viability was evaluated using an MTT colorimetric assay [41]. RAW 264.7 macrophages were inoculated onto 96-well plates (at a concentration of 1 × 10^4^ cells per well) and allowed to adhere to the bottom of the plates and incubated in an incubator for 24 h. Then, the cells were treated with or without compounds at 10 μM and incubated for 18 h, and then the cells with DMEM medium containing 1 mg/mL MTT (Sigma-Aldrich, St. Louis, MO, USA) were incubated for 4 h. After that, DMSO was added to solubilize formazan precipitates. The optical density (OD) at 540 nm was measured using a SpectraMax M5 microplate reader (Molecular Devices, Sunnyvale, CA, USA). The calculation equation for relative cell viability is as follows: cell viability (%) = (As − A0)/(Ac − A0) × 100%, where As, A0, and Ac are the absorptions of test sample, blank control, and negative control (DMSO).

### 3.7. Measurement of Nitric Oxide (NO) Production

RAW 264.7 macrophages were inoculated onto 96-well plates (at a concentration of 1 × 10^4^ cells per well) and allowed to adhere to the bottom of the plates and incubated in an incubator for 24 h. The cells were then treated with different concentrations of compounds or vehicle (DMSO) for 1 h, followed by stimulation with 1 μg/mL LPS. DMSO was used as vehicle, with the final concentration of DMSO being maintained at 0.1% of all cultures. After 18 h incubation, the supernatant was collected to determine NO content using Griess reagent (Sigma, St. Louis, MO, USA), as described previously. The absorbance at 490 nm was measured using a SpectraMax M5 microplate reader (Molecular Devices, Sunnyvale, CA, USA) [42].

### 3.8. Statistical Analysis

All data were expressed as mean ± SEM based on at least three independent experiments and analyzed using GraphPad Prism 6 (GraphPad Software, San Diego, CA, USA). One-way ANOVA was used for statistical comparison, and *p*-values less than 0.05 were considered statistically significant.

## 4. Conclusions

In summary, for the first time, a phytochemical investigation of *A. divaricate* was carried out, leading to the characterization of 39 sesquiterpenes, including 9 new ones. The structures of the isolated compounds involve germacrane-, eudesmane-, and bisabolane-type sesquiterpenes, which is consistent with the chemical constituents isolated from other *Artemisia* plants. The structure elucidation of the new compounds, especially the stereochemistry, were greatly supported by DFT NMR calculations. As a matter of fact, due to the existence of the flexible 10-membered ring of the germacrane-type sesquiterpenes, the NOESY correlations could not be used to convince the relative configurations. DFT NMR calculation could provide a powerful tool to figure out the relative configuration. Given the biogenetic relationship, absolute configuration could be further proposed. In the anti-inflammatory activity assay, compounds **2** and **8** showed a significant inhibitory effect on NO production in LPS-stimulated RAW 264.7 macrophages, with IC_50_ values of 5.35 ± 0.75 and 7.68 ± 0.54 µM, respectively. Our findings provide the first understanding of the chemical constituents of the medicinal plant *A. divaricate* and enrich the structural diversity of the sesquiterpenes of the *Artemisia* plants.

## Figures and Tables

**Table 1:** ^13^C NMR data of compounds **1**–**9** (*δ* in ppm, measured in CDCl_3_).

No.	1	2	3	4	5	6	7	8	9
1	75.6	73.3	78.5	41.2	40.9	67.7	76.2	76.2	37.3
2	32.8	32.3	31.8	24.8	24.8	29.8	27.8	27.8	23.7
3	122.1	120.1	34.9	123.4	132.3	22.7	38.0	38.0	77.7
4	134.5	134.7	144.6	127.2	127.3	150.0	74.0	73.7	144.5
5	52.0	45.3	54.8	45.4	45.3	74.6	138.0	138.0	41.9
6	71.3	71.5	68.5	77.4	76.7	31.2	130.9	130.9	81.8
7	57.2	57.6	54.8	55.8	58.8	42.2	52.0	52.0	49.7
8	70.0	70.3	69.6	132.5	125.0	28.7	31.6	31.6	73.9
9	40.2	39.3	41.9	147.1	145.1	128.4	30.5	30.5	77.1
10	38.9	38.7	41.1	73.0	72.9	136.0	150.3	151.4	69.8
11	138.6	138.6	138.5	139.6	42.2	147.1	148.0	148.0	138.7
12	167.0	166.9	166.9	170.6	178.1	23.5	21.5	21.5	168.8
13	128.9	128.8	128.2	121.1	12.6	110.5	109.3	109.3	120.6
14	24.2	24.8	12.4	23.3	23.3	16.5	110.6	110.5	20.6
15	11.5	14.3	109.1	17.8	17.8	113.0	29.3	29.3	118.2
OAc	170.3 21.2	170.5 21.2	170.3 21.2						173.0 20.6
1′	61.2	61.3	61.1						
2′	14.3	17.6	14.3						

**Table 2:** ^1^H NMR data of compounds **1**–**5** (*δ* in ppm, *J* in Hz, measured in CDCl_3_).

No	1	2	3	4	5
1	3.61 (dd, 10.1, 7.1)	3.40–3.36 (m)	3.47 (dd, 11.6, 4.7)	1.72 (2H, m)	1.74–1.68 (2H, m)
2	2.36–2.45 (m)	2.46 (overlapped)	1.88 (m)	2.27 (m)	2.25 (m)
	1.90 (overlapped)	2.02 (ddd, 10.5, 4.5, 2.2)	1.58 (overlapped)	2.17 (m)	2.17–2.11 (m)
3	5.34 (dd, 4.2, 2.3)	5.30 (dd, 4.5, 2.2)	2.36 (ddd, 13.6, 6.4, 3.7) 2.09 (ddd, 14.9, 13.6, 5.3)	5.18 (dd, 8.2, 8.1)	5.10 (dd, 11.1, 2.1)
5	1.97 (overlapped)	2.26 (d, 10.9)	1.83 (d, 10.4)	2.77 (dd, 12.2, 2.9) 2.61 (dd, 12.2, 12.2)	2.73 (dd, 12.1, 2.9) 2.49 (dd, 12.1, 9.9)
6	4.03 (dd, 11.4, 10.4)	4.07 (dd, 10.9, 10.5)	4.23 (dd, 10.4, 10.4)	3.97 (ddd, 12.2, 9.8, 2.9)	3.99 (ddd, 12.1, 9.9, 2.9)
7	2.50 (dd, 11.7, 10.4)	2.50 (dd, 11.4, 10.5)	2.62 (dd, 11.3, 10.4)	3.57 (dd, 9.8, 8.8)	2.62 (dd, 12.1, 9.5)
8	5.41 (ddd, 11.7, 11.1, 4.8)	5.43 (ddd, 11.8, 11.4, 5.2)	5.33 (ddd, 11.3, 11.2, 4.6)	5.15 (dd, 16.1, 8.8)	5.16 (dd, 16.0, 9.5)
9	2.32 (dd, 12.2, 4.8) 1.19 (dd, 12.2, 11.1)	1.88 (dd, 12.2, 11.8) 1.65 (dd, 12.2, 5.2)	2.34–2.31 (m) 1.29 (dd, 11.2, 4.9)	5.71 (d, 16.1)	5.60 (d, 16.0)
11					2.43 (dq, 12.1, 6.9)
12					
13	6.35 (s) 5.73 (s)	6.34 (d, 1.2) 5.72 (d, 1.2)	6.33 (d, 1.1) 5.71 (d, 1.1)	6.17 (d, 3.3) 5.46 (d, 3.3)	1.19 (3H, d, 6.9)
14	0.94 (3H. s)	0.94 (3H, s)	0.88 (3H, s)	1.39 (3H, s)	1.37 (3H, s)
15	1.86 (3H, s)	1.91 (3H, br s)	5.03 (d, 1.6) 4.76 (d, 1.6)	1.50 (3H, overlapped)	1.60 (3H, s)
OAc	1.95 (3H, s)	1.95 (3H, s)	1.95 (3H, s)		
1′	4.25 (2H, q, 7.1)	4.24 (2H, q, 7.1)	4.25 (2H, q, 7.1)		
2′	1.33 (3H, t, 7.1)	1.32 (3H, t, 7.1)	1.32 (3H, t, 7.1)		

**Table 3:** ^1^H NMR data of compounds **6**–**9** (*δ* in ppm, *J* in Hz, measured in CDCl_3_).

No.	6	7	8	9
1	4.91 (dd, 11.5, 5.3)	4.10 (dd, 7.9, 3.3)	4.11 (dd, 7.8, 3.2)	1.73–1.67 (m) 1.66 (ddd, 10.8, 5.3, 2.9)
2	2.16 (m)	1.91 (m)	1.91 (dd, 7.5, 3.2)	2.06 (2H, m)
	1.81–1.75 (m)	1.78 (overlapped)	1.80–1.76 (m)	
3	2.09 (m) 1.90 (ddd, 17.3, 13.6, 4.0)	1.69 (overlapped) 1.59 (ddd, 12.0, 7.7, 3.8)	1.60 (m) 1.57 (m)	4.47 (dd, 4.8, 2.3)
5	4.10 (dd, 11.0, 3.1)	5.51 (d, 16.0)	5.51 (d, 16, 0)	2.97 (br s) 2.51 (dd, 12.8, 10.8)
6	2.04 (ddd, 14.3, 11.0, 3.3) 1.74 (m)	5.10 (dd, 16.0, 10.1)	5.11 (dd, 16.0, 10.1)	4.16 (ddd, 10.8, 9.2, 1.7)
7	2.26 (m)	2.59 (ddd, 11.8, 10.1, 5.0)	2.60 (ddd, 11.3, 10.1, 5.0)	3.59 (dd, 9.2, 8.9)
8	2.66 (ddd, 12.8, 12.6, 11.8) 1.83 (overlap)	1.96 (m) 1.80 (m)	2.00–1.93 (m) 1.84–1.80 (m)	5.32 (dd, 8.9, 2.2)
9	5.25 (d, 11.8)	2.38–2.28 (m) 1.70 (overlapped)	2.34 (m) 1.70 (overlapped)	3.00 (d, 2.2)
11				
12	1.85 (3H, s)	1.72 (3H, s)	1.73 (3H, s)	
13	4.89 (s) 4.64 (s)	4.72 (2H, s)	4.73 (2H, s)	6.18 (d, 3.2) 5.40 (d, 3.2)
14	1.71 (3H, s)	5.16 (s) 4.99 (s)	5.00 (d, 1.9) 5.17 (d, 1.9)	1.23 (3H, s)
15	5.17 (s) 5.01 (s)	1.30 (3H, s)	1.31 (3H, s)	5.39 (d, 2.0) 5.16 (d, 2.0)
OAc				2.21 (3H, s)

## Data Availability

All data generated or analyzed during this study are included in this published article.

## Supplementary Materials

The following supporting information can be downloaded at: https://www.mdpi.com/article/10.3390/molecules28104254/s1, Structures of known compounds **10**–**38**; 1D and 2D NMR data, IR spectra, and HRESIMS spectra of new compounds (**1**–**9**); structures of possible isomers of compounds **1**, **2**, and **4**–**9**; DP4+ probability statistics of compounds **1**, **2**, and **4**–**9**.

## Sample Availability

Samples of the compounds **1**–**9** are available from the authors.

## References

[1] Newman D.J., Cragg G.M. (2020). Natural Products as Sources of New Drugs over the Nearly Four Decades from 01/1981 to 09/2019. J. Nat. Prod..

[2] Ivanescu B., Miron A., Corciova A. (2015). Sesquiterpene Lactones from *Artemisia* Genus: Biological Activities and Methods of Analysis. J. Anal. Methods Chem..

[3] Martínez M.J.A., Olmo L., Ticona L.A., Benito P.B. (2012). The *Artemisia* L. Genus: A Review of Bioactive Sesquiterpene Lactones. Stud. Nat. Prod. Chem..

[4] Schmidt T.J., Khalid S.A., Romanha A.J., Alves T.M.A., Biavatti M.W., Brun R., Da C.F.B., De Castro S.L., Ferreira V.F., De Lacerda M.V.G. (2012). The potential of secondary metabolites from plants as drugs or leads against protozoan neglected diseases—Part I. Curr. Med. Chem..

[5] Ke Z., Chen X.Q., Zheng M.B., Jian Y., Tang P.F. (2020). Cytotoxic sesquiterpene lactones from *Artemisia myriantha*. Phytochem. Lett..

[6] Shu W., Jian S., Ke Z., Chen X.Q., Zheng M.B., Jian Y., Tang P.F. (2014). Sesquiter-penes from *Artemisia argyi*: Absolute Configurations and Biological Activities. Eur. J. Org. Chem..

[7] Reinhardt J.K., Klemd A.M., Danton O., De Mieri M., Smieško M., Huber R., Bürgi T., Gründemann C., Hamburger M. (2019). Sesquiterpene Lactones from *Artemisia argyi*: Absolute Configuration and Immunosuppressant Activity. J. Nat. Prod..

[8] Bai M., Chen J., Xu W., Dong S., Liu Q., Lin B., Huang X., Yao G., Song S. (2020). Elephantopinolide A-P, germacrane-type sesquiter-pene lactones from *Elephantopus scaber* induce apoptosis, autophagy and G2/M phase arrest in hepatocellular carcinoma cells. Eur. J. Med. Chem..

[9] Xu W., Bai M., Liu D., Qin S., Lv T., Li Q., Lin B., Song S., Huang X. (2022). MS/MS-based molecular networking accelerated discovery of germacrane-type sesquiterpene lactones from *Elephantopus scaber* L.. Phytochemistry.

[10] Perveen S., Alqahtani J., Orfali R., Aati H.Y., Al-Taweel A.M., Ibrahim T.A., Khan A., Yusufoglu H.S., Abdel-Kader M.S., Taglialatela-Scafati O. (2020). Antibacterial and Antifungal Sesquiterpenoids from Aerial Parts of *Anvillea garcinii*. Molecules.

[11] Zhu N., Tang C., Xu C., Ke C., Lin G., Jenis J., Yao S., Liu H., Ye Y. (2019). Cytotoxic Germacrane-Type Sesquiterpene Lactones from the Whole Plant of *Carpesium lipskyi*. J. Nat. Prod..

[12] Yan C., Long Q., Zhang Y., Babu G., Krishnapriya M.V., Qiu J., Song J., Rao Q., Yi P., Sun M. (2021). Germacranolide sesquiterpenes from *Carpesium cernuum* and their anti-leukemia activity. Chin. J. Nat. Med..

[13] Wu Z.Y., Raven P.H., Hong D.Y. (2011). Flora of China.

[14] (2013). Computer Program.

[15] (2017). Computer Program.

[16] Grimblat N., Zanardi M.M., Sarotti A.M. (2015). Beyond DP4: An Improved Probability for the Stereochemical Assignment of Isomeric Compounds using Quantum Chemical Calculations of NMR Shifts. J. Org. Chem..

[17] Chi J., Li B., Dai W., Liu L., Zhang M. (2016). Highly oxidized sesquiterpenes from *Artemisia austroyunnanensis*. Fitoterapia.

[18] Lee T., Lee S., Kim K.H., Oh K., Shin J., Mar W. (2013). Effects of magnolialide isolated from the leaves of *Laurus nobilis* L. (Laura-ceae) on immunoglobulin E-mediated type I hypersensitivity in vitro. J. Ethnopharmacol..

[19] Yang Y., Liu J., Li Z., Li Y., Qiu M. (2013). New eudesmenoic acid methyl esters from the seed oil of *Jatropha curcas*. Fitoterapia.

[20] Galal A.M. (2001). Microbial Transformation of Pyrethrosin. J. Nat. Prod..

[21] Zhen X., Wang X., Wang C.P., Li G.H. (2016). Chemical constituents and biological activities of *Pyrethrum cinerariifolium*. Guihaia.

[22] Xue G.M., Zhao C.G., Xue J.F., Xing G.F., Zhao Z.Z., Du K., Si Y.Y., Sun Y.J., Feng W.S. (2022). Chemical constituents from seeds of *Artemisia argyi*. Chin. Tradit. Herbal Drugs.

[23] Zhu Y., Zhang L., Zhao Y., Huang G. (2010). Unusual sesquiterpene lactones with a new carbon skeleton and new acetylenes from *Ajania przewalskii*. Food Chem..

[24] Yang H., Xie J.L., Sun H.D. (1997). Study on chemical constituents from the roots of *Saussurea lappa*. Chin. J. Chin. Mater. Med..

[25] Konstantinopoulou M., Karioti A., Skaltsas S., Skaltsa H. (2003). Sesquiterpene Lactones from *Anthemisa ltissima* and Their Anti-Helicobacter pylori Activity. J. Nat. Prod..

[26] Bethencourt-Estrella C.J., Nocchi N., López-Arencibia A., San Nicolás-Hernández D., Souto M.L., Suárez-Gómez B., Díaz-Marrero A.R., Fernández J.J., Lorenzo-Morales J., Piñero J.E. (2021). Antikinetoplastid Activity of Sesquiterpenes Isolated from the *Zoanthid Palythoa aff. clavata*. Pharmaceuticals.

[27] Lee K.D., Yang M.S., Ha T.J., Park K.M., Park K.H. (2002). Isolation and Identification of Dihydrochrysanolide and Its 1-Epimer from *Chrysanthemum coro-narium* L.. Biosci. Biotechnol. Biochem..

[28] Izbosarov M.B., Abduazimov B.K., Yusupova I.M., Tashkhodzhaev B., Abdullaev A.D.V.D. (1998). Germacranolide from *Tanacetopis mucronata*. Chem. Nat. Commun..

[29] Fraga B.M., Terrero D., Cabrera I., Reina M. (2018). Studies on the sesquiterpene lactones from *Laurus novocanariensis* lead to the clarification of the structures of 1-epi-tatridin B and its epimer tatridin B. Phytochemistry.

[30] Bai L., Liu Q., Cen Y., Huang J., Zhang X., Guo S., Zhang L., Guo T., Ho C.T., Bai N. (2018). A new sesquiterpene lactone glucoside and other constituents from *Inula salsoloides* with insecticidal activities on striped flea beetle (*Phyllotreta striolata* Fabricius). Nat. Prod. Res..

[31] Ito M.H.A.K. (1981). Regio- and Stereo-specific Allylic Oxidation of Germacrane-type Sesquiterpene Lactones with Selenium. Diox-ide and t-Butyl Hydroperoxide. J. Chem. Soc. Chemi. Commun..

[32] Cimmino A., Roscetto E., Masi M., Tuzi A., Radjai I., Gahdab C., Paolillo R., Guarino A., Catania M.R., Evidente A. (2021). Sesquiterpene Lactones from *Cotula cinerea* with Antibiotic Activity against Clinical Isolates of *Enterococcus faecalis*. Antibiotics.

[33] Triana J., Eiroa J.L., Morales M., Perez F.J., Brouard I., Marrero M.T., Estevez S., Quintana J., Estevez F., Castillo Q.A. (2013). A chemo-taxonomic study of endemic species of genus Tanacetum from the Canary Islands. Phytochemistry.

[34] Zhao M., Zhang X., Wang Y., Huang M., Duan J.A., Godecke T., Szymulanska-Ramamurthy K.M., Yin Z., Che C.T. (2014). Germacranes and m-menthane from *Illicium lanceolatum*. Molecules.

[35] Triana J., Lopez M., Rico M., Gonzalez-Platas J., Quintana J., Estevez F., Leon F., Bermejo J. (2003). Sesquiterpenoid derivatives from *Gonospermum elegans* and their cytotoxic activity for HL-60 human promyelocytic cells. J. Nat. Prod..

[36] Sanz J.F., García-Sarrión A., Marco J.A. (1991). Germacrane derivatives from *Santolina chamaecyparissus*. Phytochemistry.

[37] Liu S., Zhang J., He F., Fu W., Tang B., Bin Y., Fang M., Wu Z., Qiu Y. (2022). Anti-inflammatory sesquiterpenoids from the heartwood of *Juniperus formosana Hayata*. Fitoterapia.

[38] D’Abrosca B., De Maria P., DellaGreca M., Fiorentino A., Golino A., Izzo A., Monaco P. (2006). Amarantholidols and amarantholidosides: New nerolidol derivatives from the weed *Amaranthus retroflexus*. Tetrahedron.

[39] Watanabe S., Alexander M., Misharin A.V., Budinger G.R.S. (2019). The role of macrophages in the resolution of inflammation. J. Clin. Investig..

[40] Li D., Zhang T., Lu J., Peng C., Lin L. (2020). Natural constituents from food sources as therapeutic agents for obesity and metabolic diseases targeting adipose tissue inflammation. Crit. Rev. Food Sci..

[41] Feng Z., Zhang L., Zheng Y., Liu Q., Liu J., Feng L., Huang L., Zhang Q., Lu J., Lin L. (2017). Norditerpenoids and Dinorditerpenoids from the Seeds of *Podocarpus nagi* as Cytotoxic Agents and Autophagy Inducers. J. Nat. Prod..

[42] Feng Z., Chen J., Feng L., Chen C., Ye Y., Lin L. (2021). Polyisoprenylated benzophenone derivatives from Garcinia cambogia and their anti-inflammatory activities. Food Funct..

