# Supplementary material for: The First Phytochemical Investigation of Artemisia divaricate: Sesquiterpenes and Their Anti-Inflammatory Activity
Source: Molecules. 2023 May 22;28(10):4254. doi: 10.3390/molecules28104254 (PMC10221161; doi:10.3390/molecules28104254)
Supplement: Supplementary file 1 [file molecules-28-04254-s001.zip › molecules-2365685-supplementary.pdf]

Supplementary data for

The first phytochemical investigation of *Artemisia divaricate*: sesquiterpenes and their anti-inflammatory activity

Siqi Yan [1,2,3], Changqiang Ke [1,2], Zheling Feng [1,2], Chunping Tang [1,2,*] and Yang Ye [1,2,3,4,*]

[1] State Key Laboratory of Drug Research, Shanghai Institute of Materia Medica, Chinese Academy of Sciences, Shanghai 201203, China; s20-yansiqi@simm.ac.cn (S.Y.); kechangqiang@simm.ac.cn (C.K.); fengzheling@simm.ac.cn (Z.F.)

[2] Natural Products Chemistry Department, Shanghai Institute of Materia Medica, Chinese Academy of Sciences, Shanghai 201203, China

[3] University of Chinese Academy of Sciences, No. 19A Yuquan Road, Beijing 100049, China

[4] School of Life Science and Technology, ShanghaiTech University, Shanghai 201203, China

* Correspondence: tangcp@simm.ac.cn (C.T.); yye@simm.ac.cn (Y.Y.)

Contents

Figure S1 Structures of known compounds **10-39** 1
Figure S2 HR-ESIMS spectrum of compound **1** 2
Figure S3 ^1H NMR spectrum of compound **1** 2
Figure S4 ^{13}C NMR spectrum of compound **1** 3
Figure S5 HSQC spectrum of compound **1** 3
Figure S6 ^1H-^1H COSY spectrum of compound **1** 4
Figure S7 HMBC spectrum of compound **1** 4
Figure S8 NOESY spectrum of compound **1** 5
Figure S9 IR spectrum of compound **1** 5
Figure S10 HR-ESIMS spectrum of compound **2** 6
Figure S11 ^1H NMR spectrum of compound **2** 6
Figure S12 ^{13}C NMR spectrum of compound **2** 7
Figure S13 HSQC spectrum of compound **2** 7
Figure S14 ^1H-^1H COSY spectrum of compound **2** 8
Figure S15 HMBC spectrum of compound **2** 8
Figure S16 NOESY spectrum of compound **2** 9
Figure S17 IR spectrum of compound **2** 9
Figure S18 Possible isomers of compound **1** and **2** 10
Figure S19 DP4+ probability statistics of compound **1** 10
Figure S20 DP4+ probability statistics of compound **2** 10
Figure S21 HR-ESIMS spectrum of compound **3** 11
Figure S22 ^1H NMR spectrum of compound **3** 11
Figure S23 ^{13}C NMR spectrum of compound **3** 12
Figure S24 HSQC NMR spectrum of compound **3** 12
Figure S25 ^1H-^1H COSY spectrum of compound **3** 13
Figure S26 HMBC spectrum of compound **3** 13
Figure S27 NOESY spectrum of compound **3** 14
Figure S28 IR spectrum of compound **3** 14
Figure S29 HR-ESIMS spectrum of compound **4** 15
Figure S30 ^1H NMR spectrum of compound **4** 15
Figure S31 ^{13}C NMR spectrum of compound **4** 16
Figure S32 HSQC spectrum of compound **4** 16
Figure S33 ^1H-^1H COSY spectrum of compound **4** 17
Figure S34 HMBC spectrum of compound **4** 17
Figure S35 NOESY spectrum of compound **4** 18
Figure S36 IR spectrum of compound **4** 18

Figure S37 Possible isomers of compound **4** 19
Figure S38 DP4+ probability statistics of compound **4** 19
Figure S39 HR-ESIMS spectrum of compound **5** 20
Figure S40 ^{1}H NMR spectrum of compound **5** 20
Figure S41 ^{13}C NMR spectrum of compound **5** 21
Figure S42 HSQC spectrum of compound **5** 21
Figure S43 ^{1}H-^{1}H COSY spectrum of compound **5** 22
Figure S44 HMBC spectrum of compound **5** 22
Figure S45 NOESY spectrum of compound **5** 23
Figure S46 IR spectrum of compound **5** 23
Figure S47 Possible isomers of compound **5** 24
Figure S48 DP4+ probability statistics of compound **5** 24
Figure S49 HR-ESIMS spectrum of compound **6** 25
Figure S50 ^{1}H NMR spectrum of compound **6** 25
Figure S51 ^{13}C NMR spectrum of compound **6** 26
Figure S52 HSQC spectrum of compound **6** 26
Figure S53 ^{1}H-^{1}H COSY spectrum of compound **6** 27
Figure S54 HMBC spectrum of compound **6** 27
Figure S55 NOESY spectrum of compound **6** 28
Figure S56 IR spectrum of compound **6** 28
Figure S57 Possible isomers of compound **6** 29
Figure S58 DP4+ probability statistics of compound **6** 29
Figure S59 HR-ESIMS spectrum of compound **7** 30
Figure S60 ^{1}H NMR spectrum of compound **7** 30
Figure S61 ^{13}C NMR spectrum of compound **7** 31
Figure S62 HSQC spectrum of compound **7** 31
Figure S63 ^{1}H-^{1}H COSY spectrum of compound **7** 32
Figure S64 HMBC spectrum of compound **7** 32
Figure S65 NOESY spectrum of compound **7** 33
Figure S66 IR spectrum of compound **7** 33
Figure S67 Possible isomers of compound **7** 34
Figure S68 DP4+ probability statistics of compound **7** 34
Figure S69 HR-ESIMS spectrum of compound **8** 35
Figure S70 ^{1}H NMR spectrum of compound **8** 35
Figure S71 ^{13}C NMR spectrum of compound **8** 36
Figure S72 HSQC spectrum of compound **8** 36
Figure S73 ^{1}H-^{1}H COSY spectrum of compound **8** 37

Figure S74 HMBC spectrum of compound **8** 37
Figure S75 NOESY spectrum of compound **8** 38
Figure S76 IR spectrum of compound **8** 38
Figure S77 Possible isomers of compound **8** 39
Figure S78 DP4+ probability statistics of compound **8** 39
Figure S79 HR-ESIMS spectrum of compound **9** 40
Figure S80 ^1H NMR spectrum of compound **9** 40
Figure S81 ^{13}C NMR spectrum of compound **9** 41
Figure S82 HSQC spectrum of compound **9** 41
Figure S83 ^1H-^1H COSY spectrum of compound **9** 42
Figure S84 HMBC spectrum of compound **9** 42
Figure S85 NOESY spectrum of compound **9** 43
Figure S86 IR spectrum of compound **9** 43
Figure S87 Possible isomers of compound **9** 44
Figure S88 DP4+ probability statistics of compound **9** 44

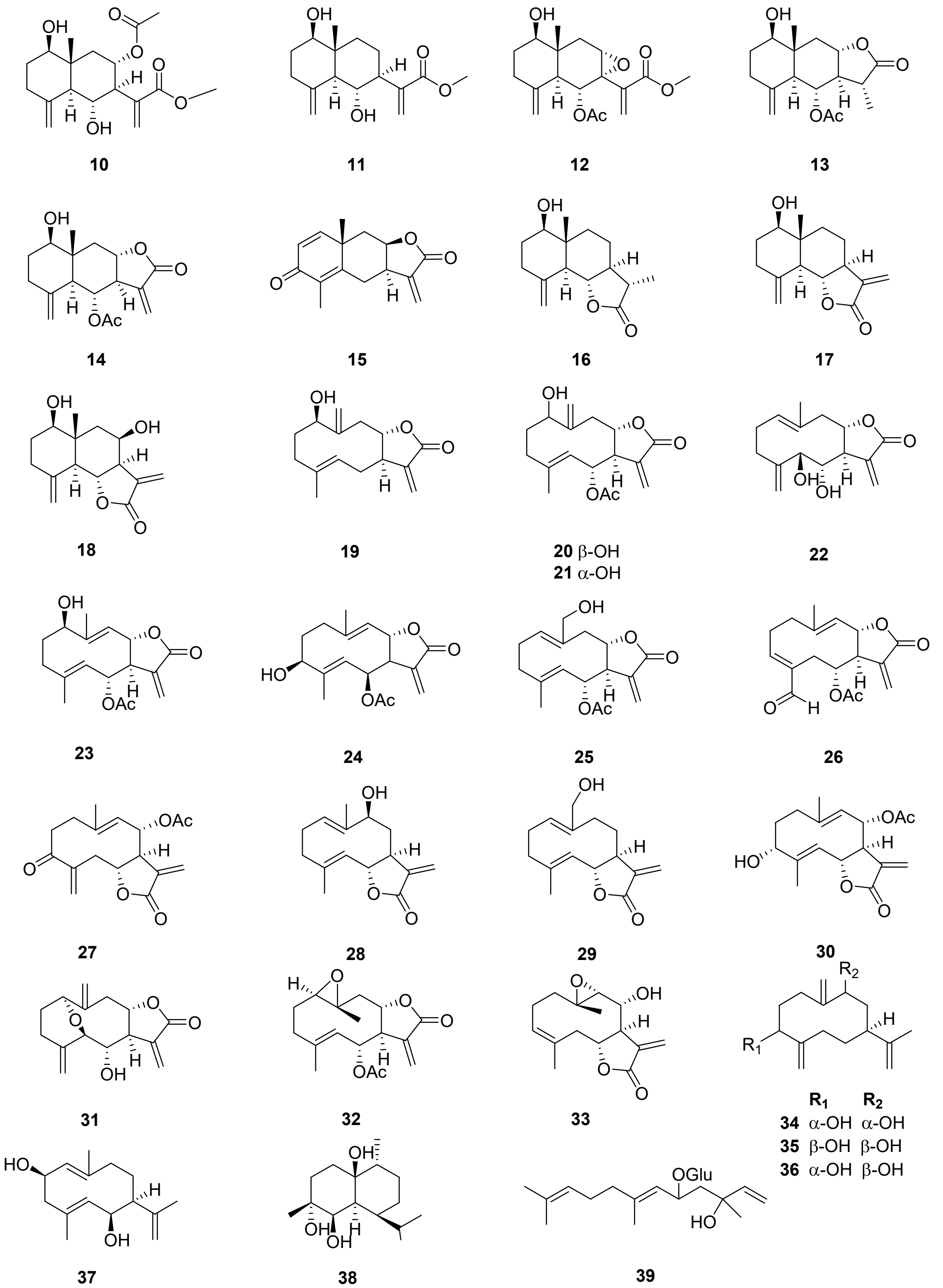

Figure S1 Structures of known compounds **10**-**39**

Elemental Composition Report
Tolerance = 20.0 PPM / DBE: min = -1.5, max = 50.0
Element prediction: Off
Number of isotope peaks used for i-FIT = 3
Monoisotopic Mass, Even Electron Ions
66 formula(e) evaluated with 2 results within limits (up to 50 best isotopic matches for each mass)
Elements Used:
C: 0-200 H: 0-60 O: 0-6 Na: 0-1

Minimum:	80.00						-1.5		
Maximum:	100.00			2.0	20.0		50.0		
Mass	RA	Calc. Mass	mDa	PPM	DBE	i-FIT	Norm	Conf(%)	Formula
375.1855	100.00	375.1784	7.1	18.9	5.5	40.1	0.164	84.84	$C_{19}H_{28}O_6Na$
		375.1808	4.7	12.5	8.5	41.9	1.887	15.16	$C_{21}H_{27}O_6$

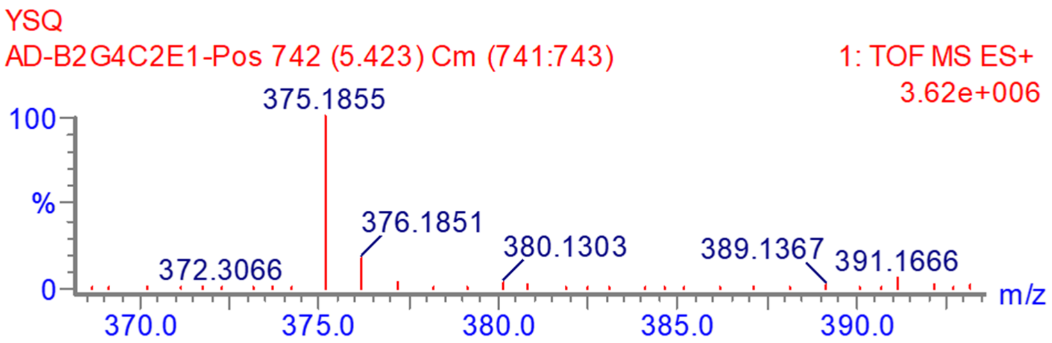

Figure S2 HR-ESIMS spectrum of compound **1**

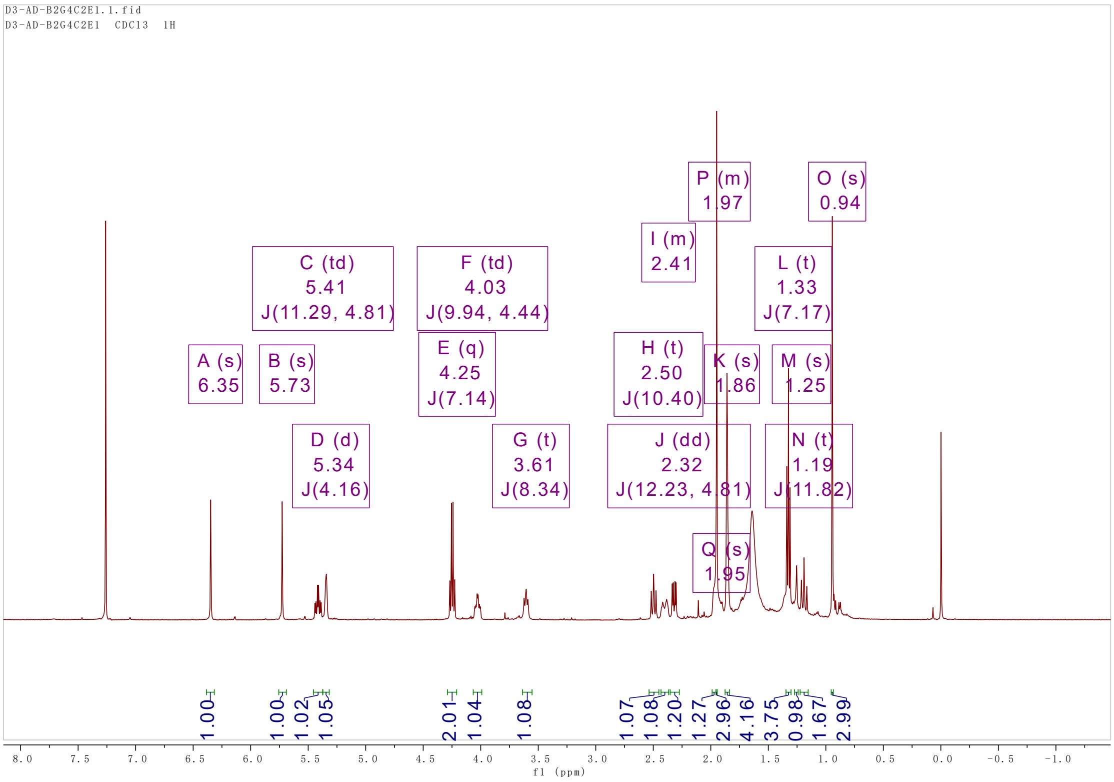

Figure S3 ^{1}H NMR spectrum of compound **1**

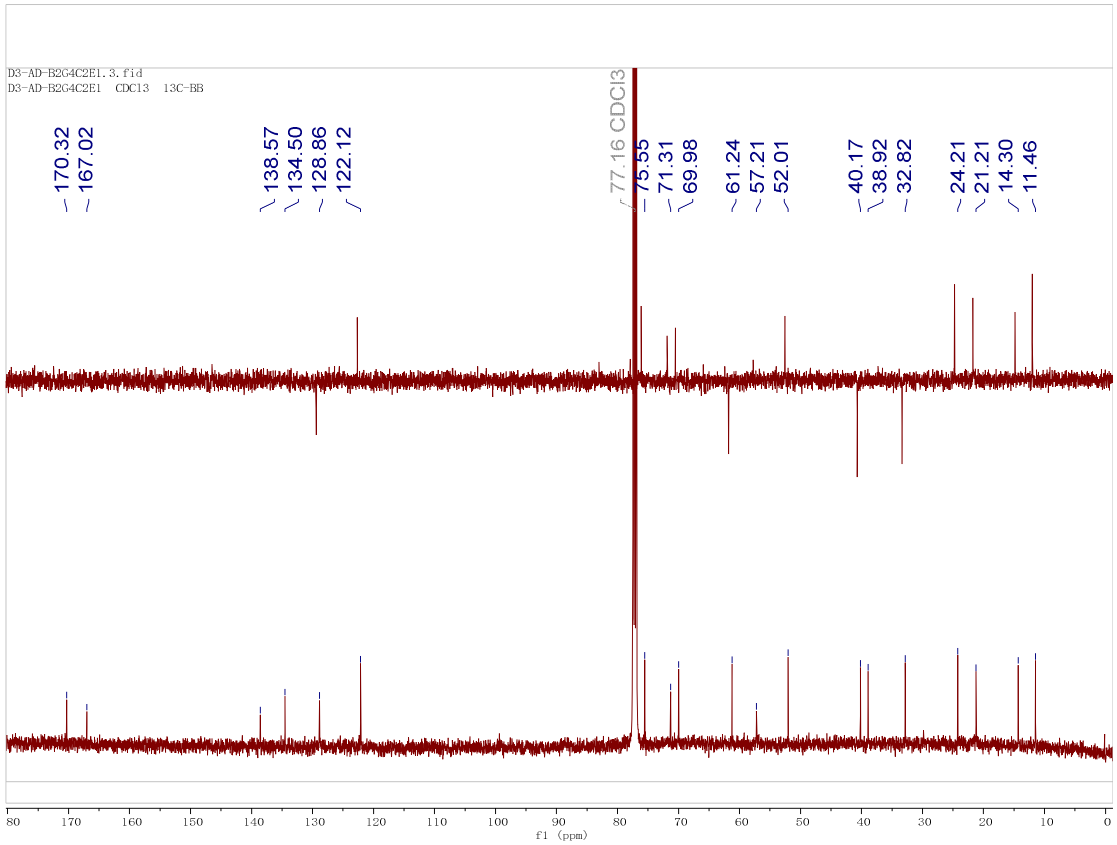

Figure S4 ^{13}C NMR spectrum of compound **1**

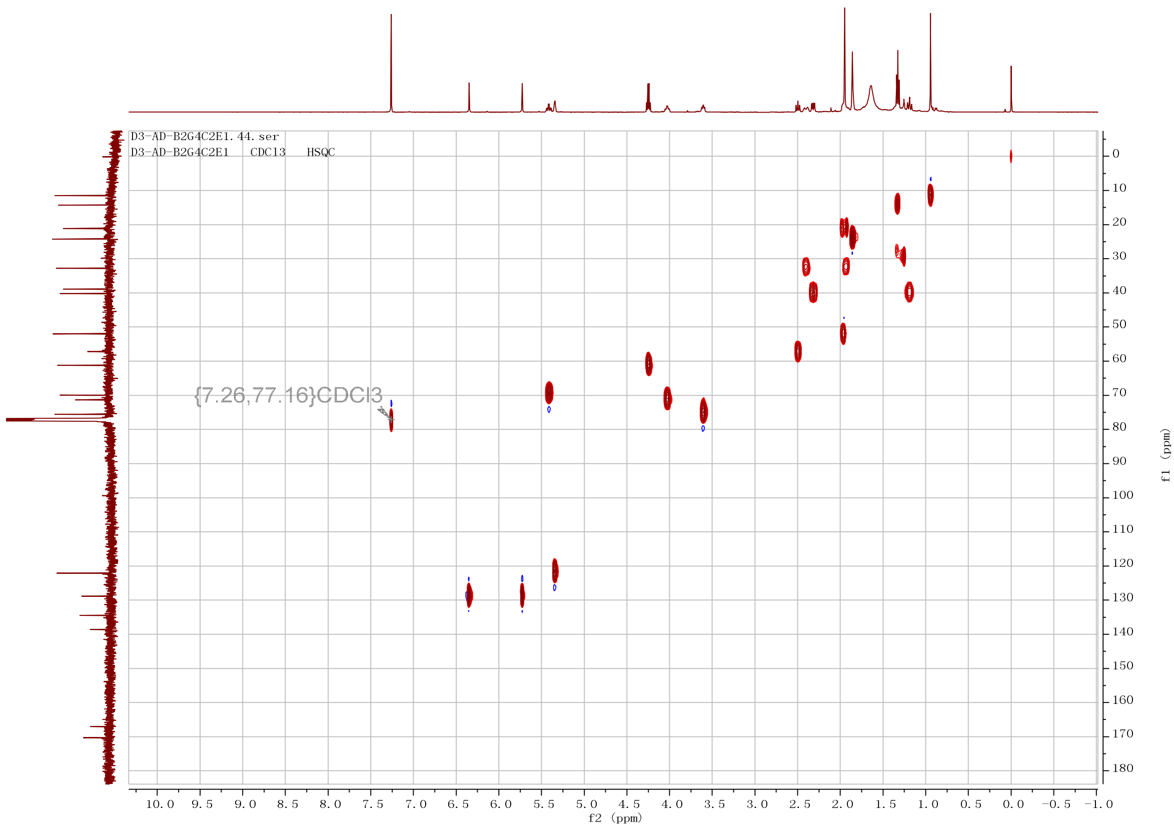

Figure S5 HSQC spectrum of compound **1**

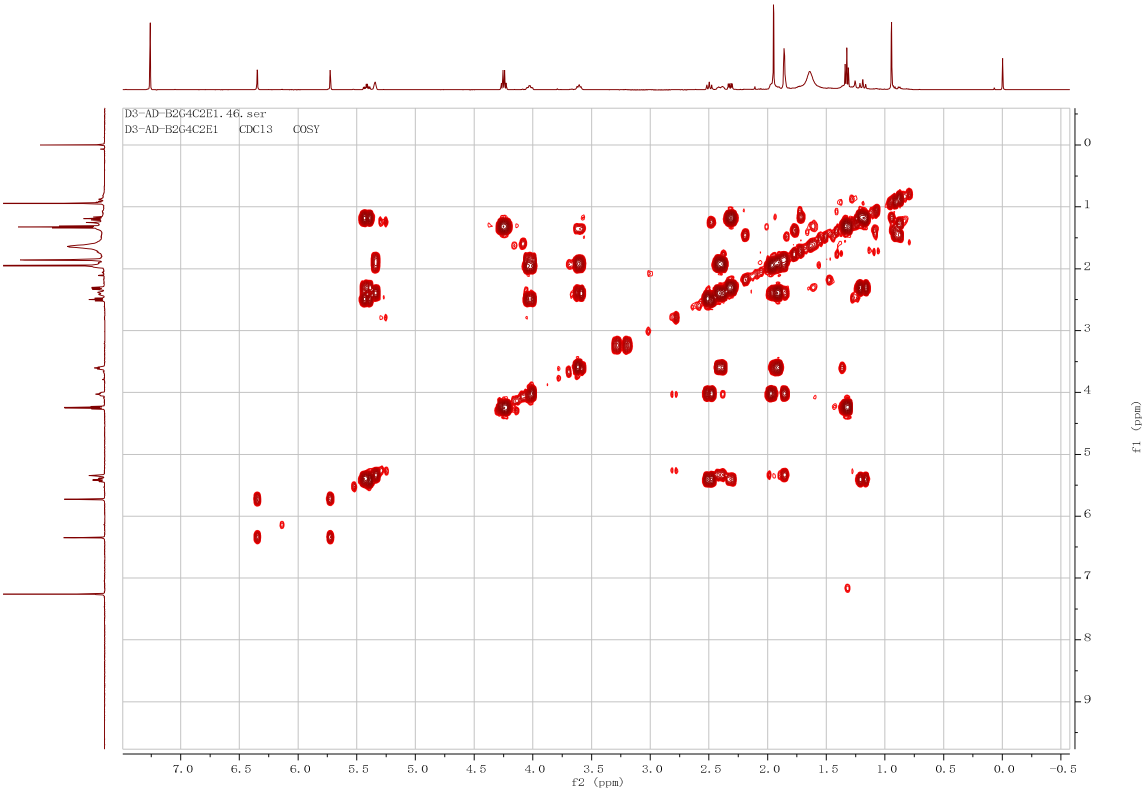

Figure S6 ¹H-¹H COSY spectrum of compound **1**

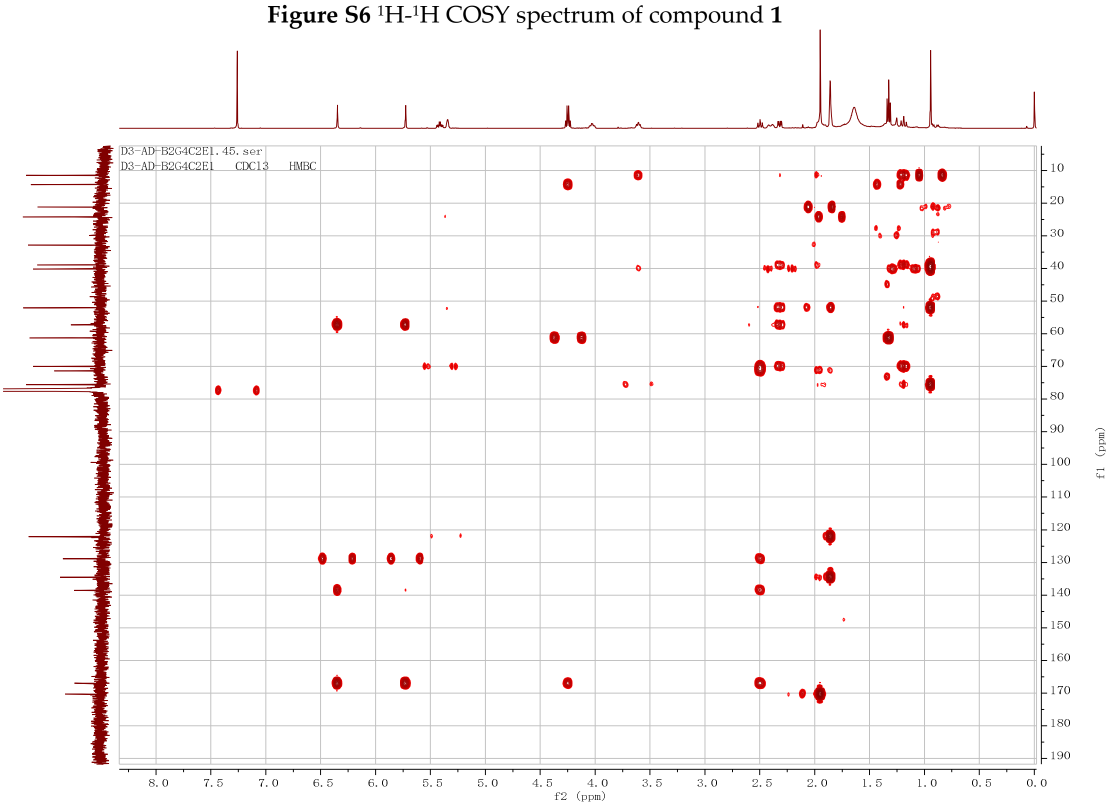

Figure S7 HMBC spectrum of compound **1**

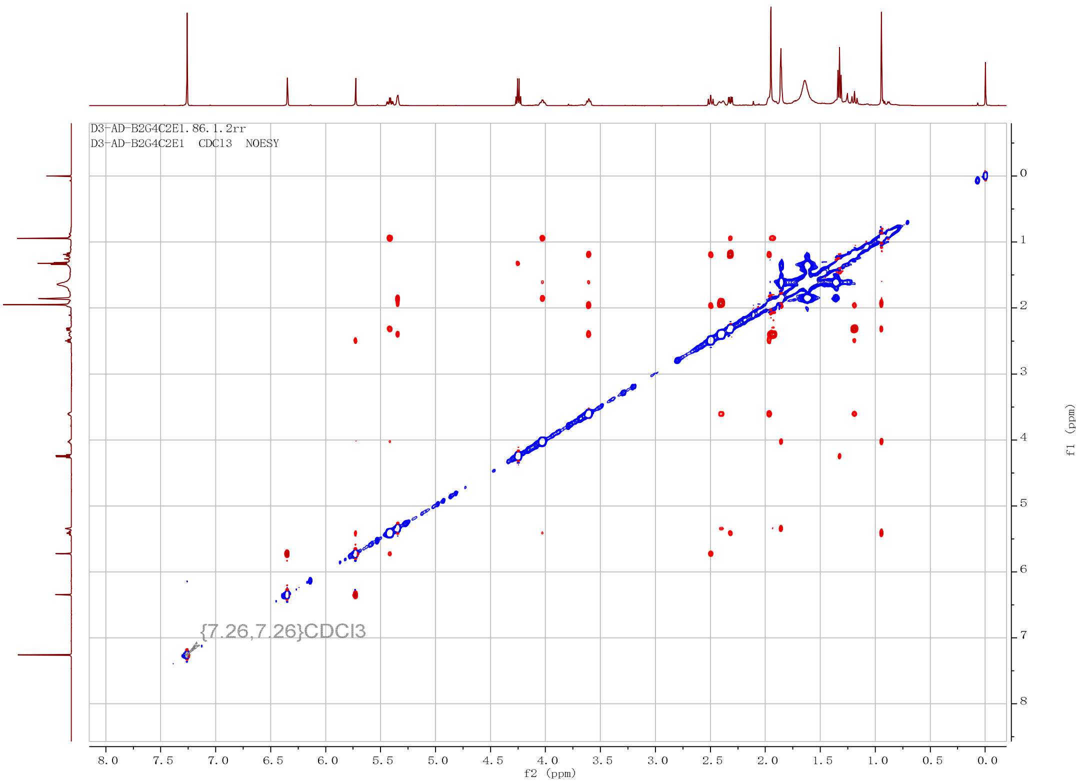

Figure S8 NOESY spectrum of compound **1**

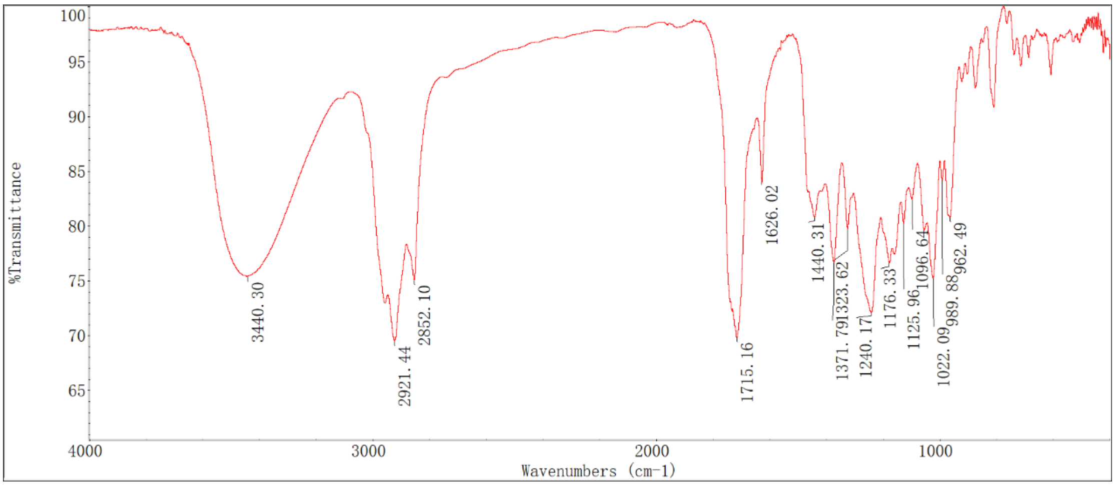

Figure S9 IR spectrum of compound **1**

Elemental Composition Report
Tolerance = 5.0 PPM / DBE: min = -1.5, max = 50.0
Element prediction: Off
Number of isotope peaks used for i-FIT = 3
Monoisotopic Mass, Even Electron Ions
66 formula(e) evaluated with 1 results within limits (up to 50 best isotopic matches for each mass)
Elements Used:
C: 0-200 H: 0-60 O: 0-6 Na: 0-1
Minimum: 80.00 -1.5
Maximum: 100.00 2.0 5.0 50.0

Mass	RA	CalcMass	mDa	PPM	DBE	i-FIT	Norm	Conf(%)	Formula
375.1783	100.0	375.1784	-0.1	-0.3	5.5	794.7	n/a	n/a	$C_{19}H_{28}O_6Na$

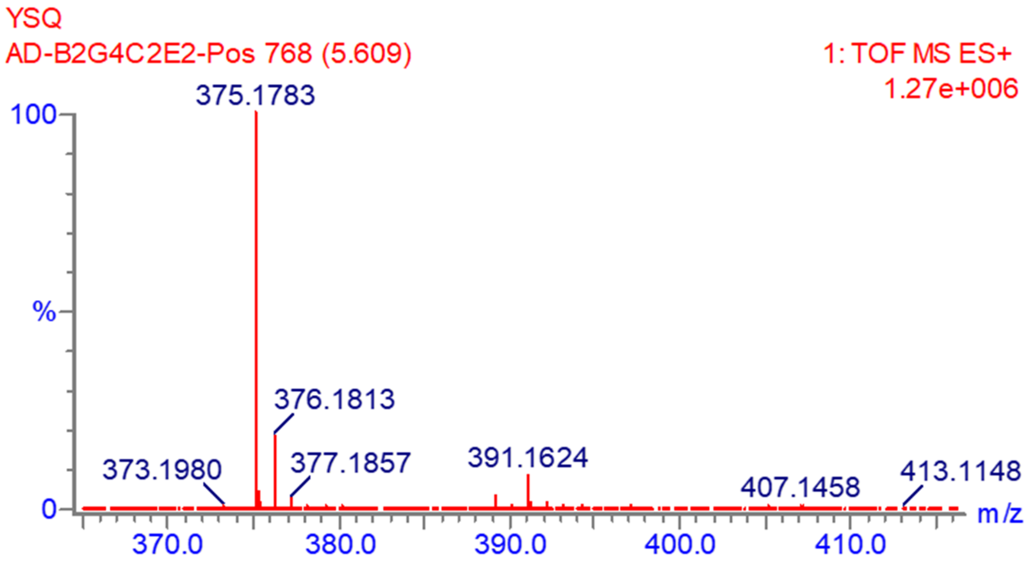

Figure S10 HR-ESIMS spectrum of compound **2**

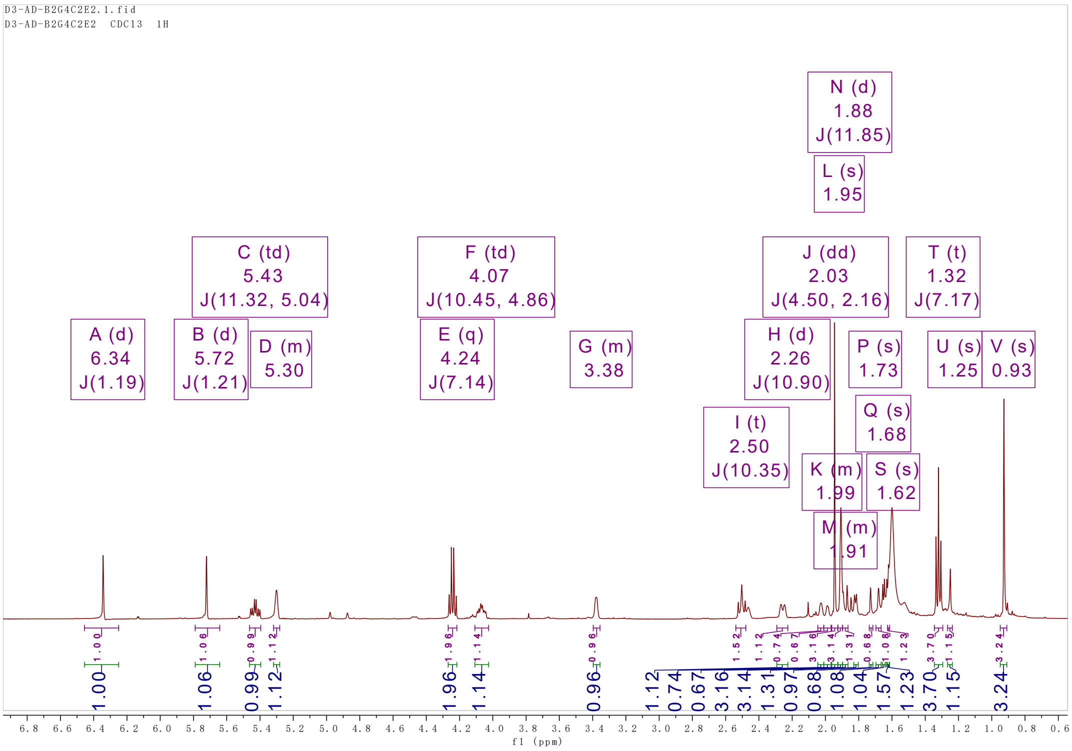

Figure S11 ^1H NMR spectrum of compound **2**

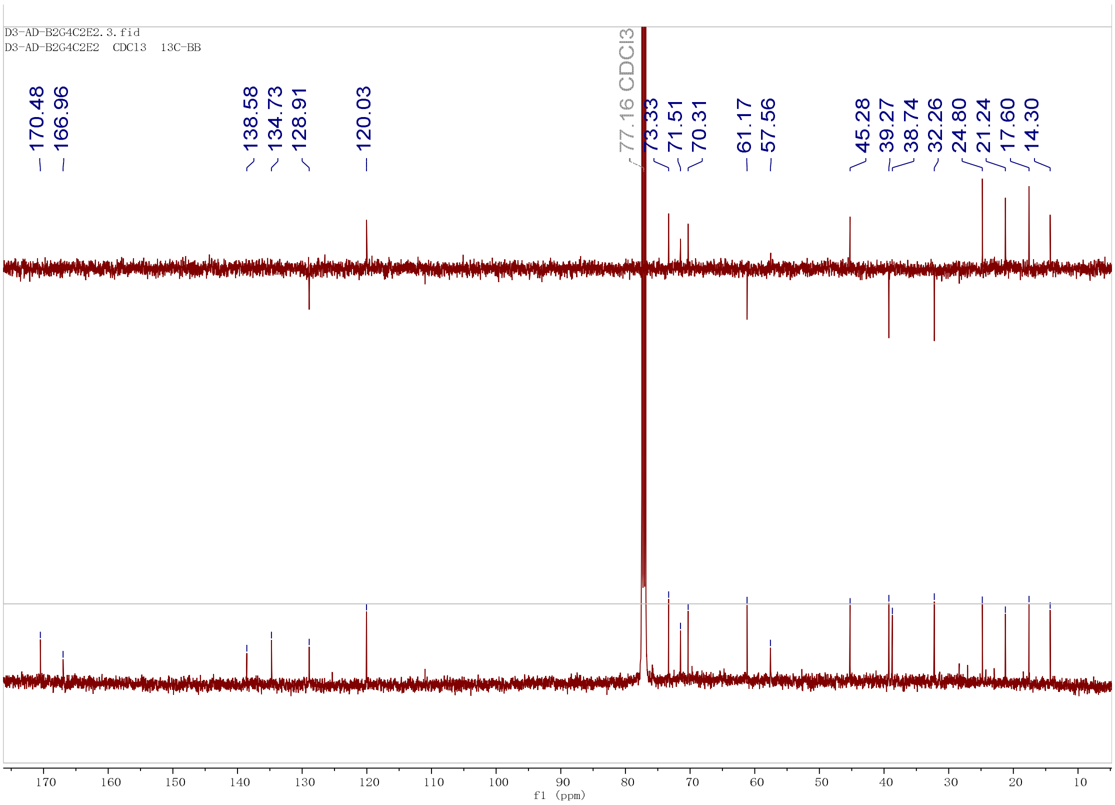

Figure S12 ^{13}C NMR spectrum of compound **2**

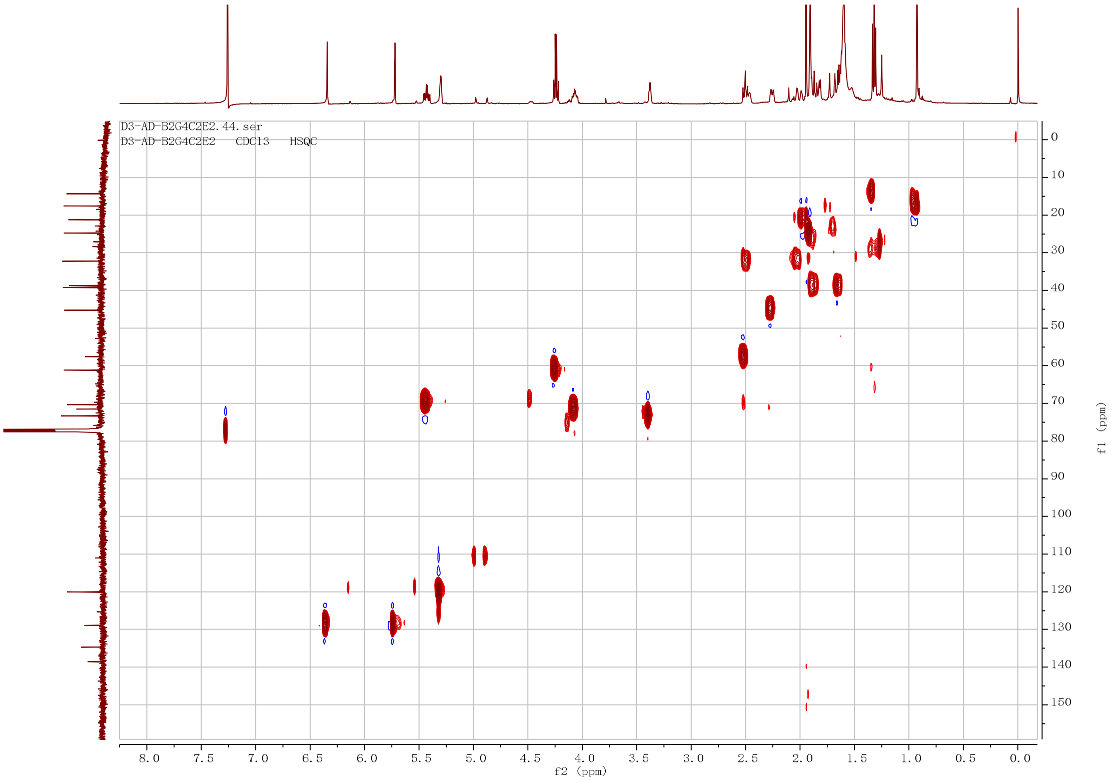

Figure S13 HSQC spectrum of compound **2**

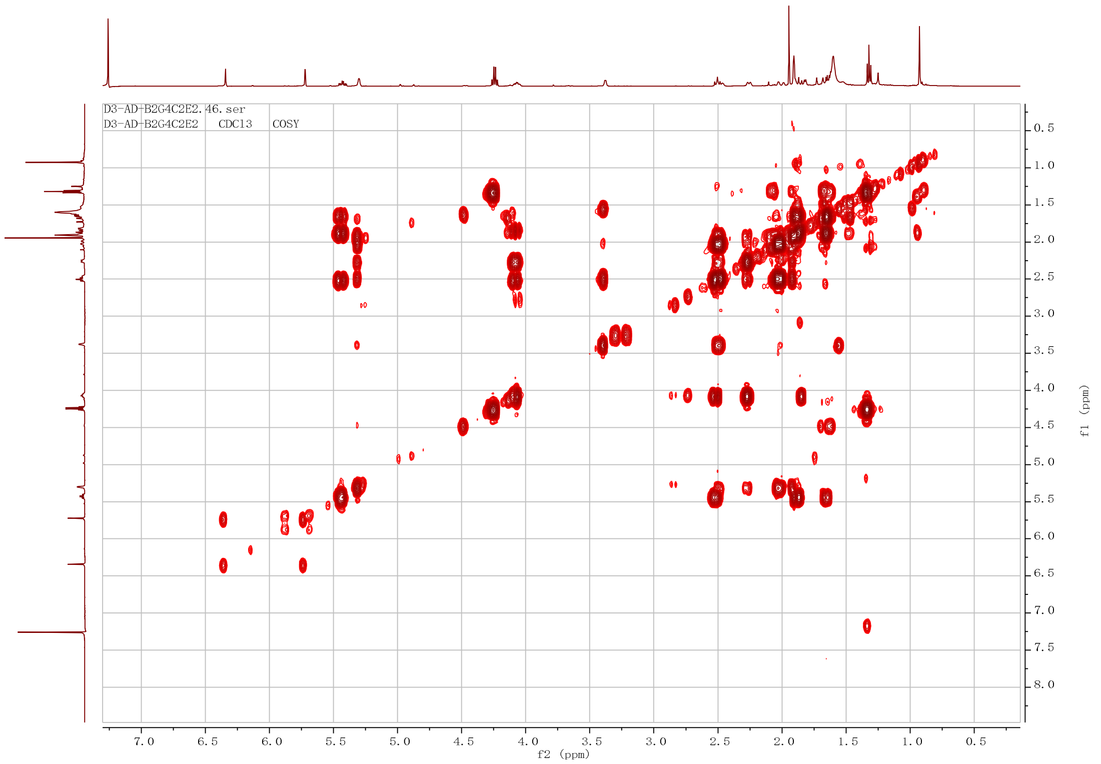

Figure S14 ^{1}H-^{1}H COSY spectrum of compound **2**

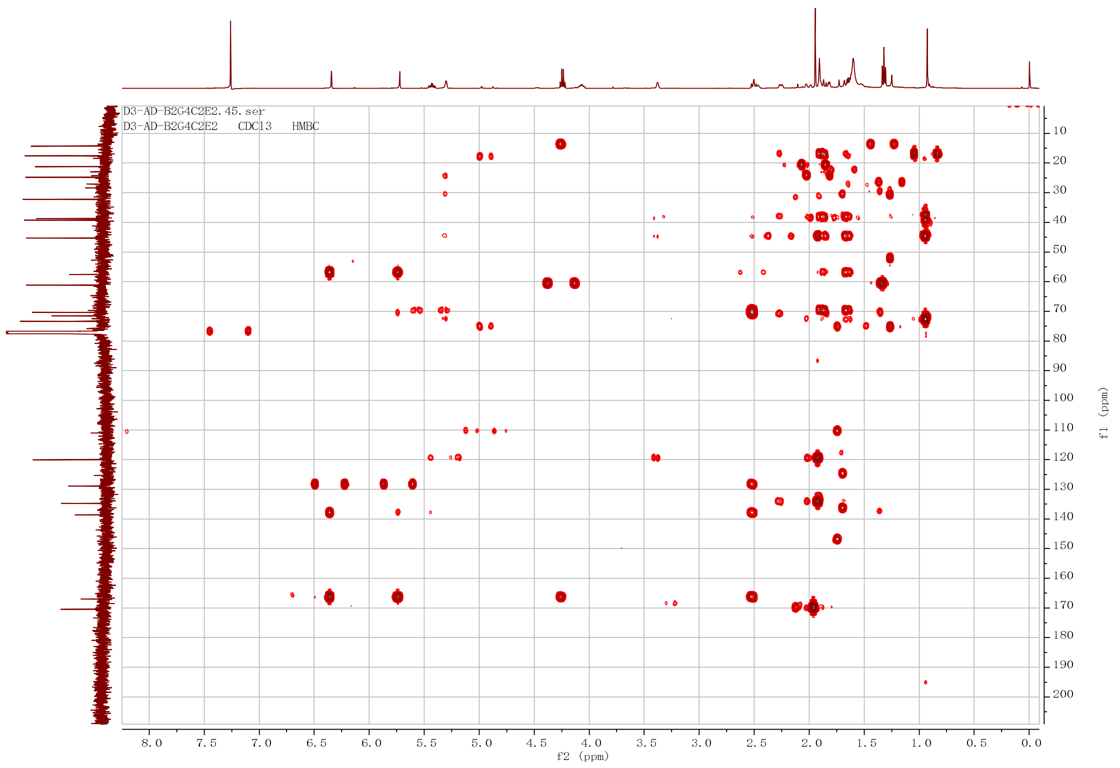

Figure S15 HMBC spectrum of compound **2**

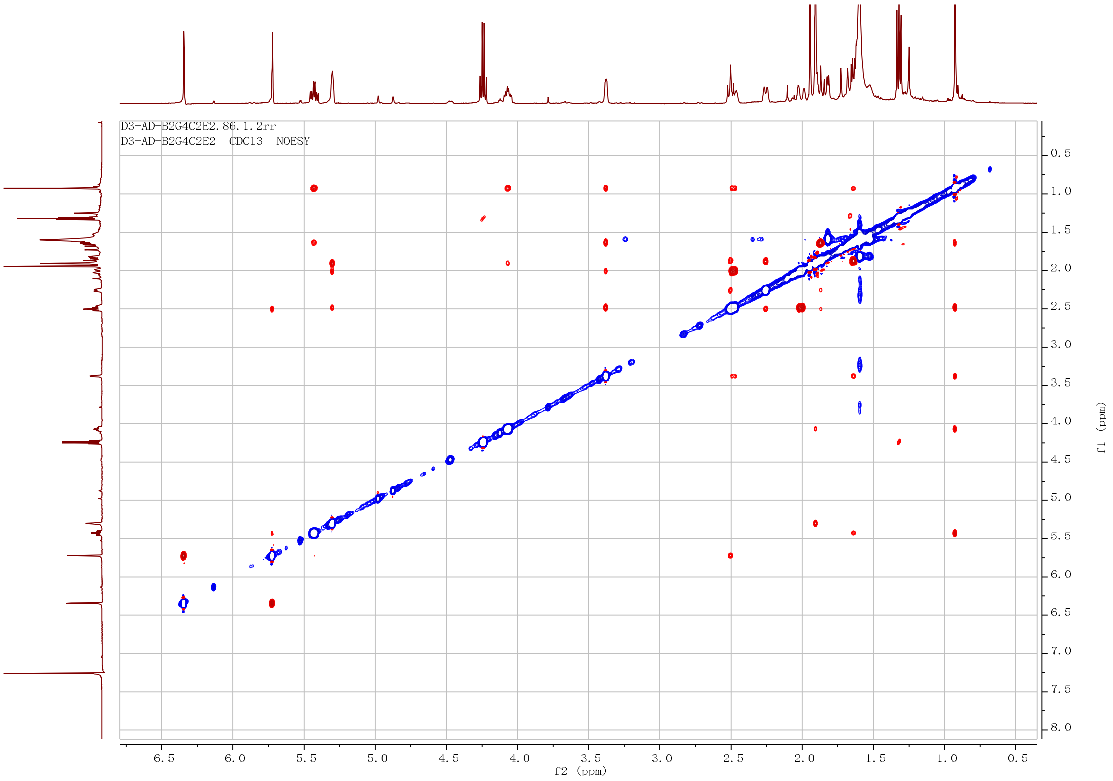

Figure S16 NOESY spectrum of compound **2**

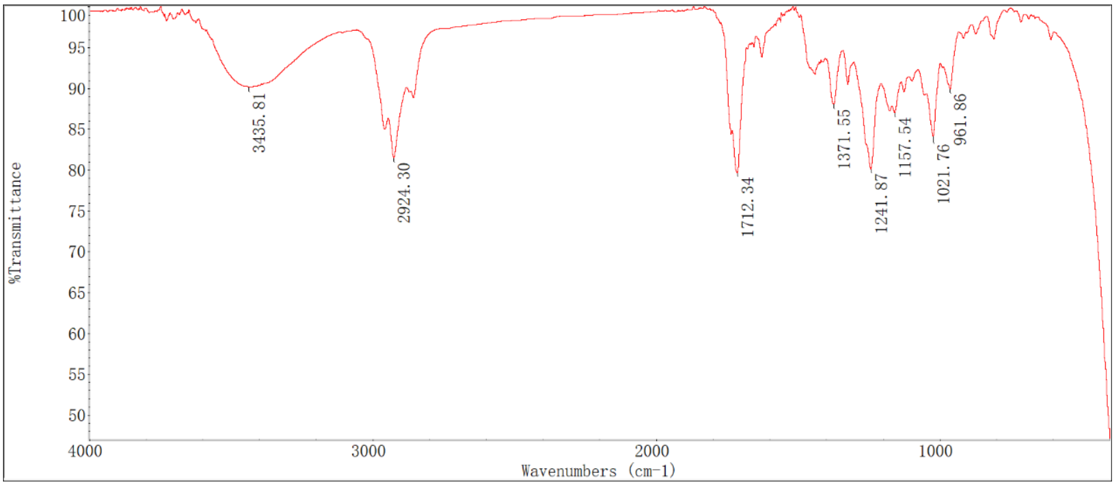

Figure S17 IR spectrum of compound **2**

truncated structures used for DFT calculation

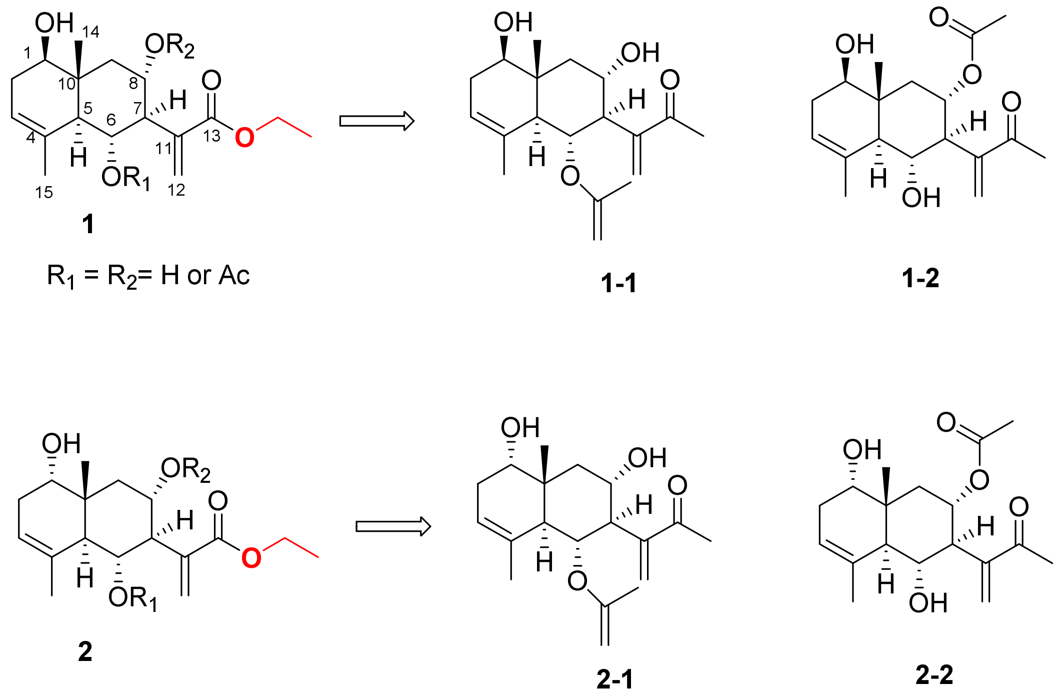

Figure S18 Possible isomers of compound **1** and **2**

Functional	Solvent?	Basis Set	
mPW1PW91	PCM	6-311G(d, p	

	Isomer 1	Isomer 2	Isomer 3	Isor
sDP4+ (H data)	0.00%	100.00%	–	
sDP4+ (C data)	73.05%	26.95%	–	
sDP4+ (all data)	0.00%	100.00%	–	
uDP4+ (H data)	0.00%	100.00%	–	
uDP4+ (C data)	91.34%	8.66%	–	
uDP4+ (all data)	0.00%	100.00%	–	
DP4+ (H data)	0.00%	100.00%	–	
DP4+ (C data)	96.62%	3.38%	–	
DP4+ (all data)	0.00%	100.00%	–	

Figure S19 DP4+ probability statistics of compound **1**

Functional	Solvent?	Basis Set	
mPW1PW91	PCM	6-311G(d, p	

	Isomer 1	Isomer 2	Isomer 3	Isor
sDP4+ (H data)	0.00%	100.00%	–	
sDP4+ (C data)	73.05%	26.95%	–	
sDP4+ (all data)	0.00%	100.00%	–	
uDP4+ (H data)	0.00%	100.00%	–	
uDP4+ (C data)	91.34%	8.66%	–	
uDP4+ (all data)	0.00%	100.00%	–	
DP4+ (H data)	0.00%	100.00%	–	
DP4+ (C data)	96.62%	3.38%	–	
DP4+ (all data)	0.00%	100.00%	–	

Figure S20 DP4+ probability statistics of compound **2**

Elemental Composition Report
Tolerance = 5.0 PPM / DBE: min = -1.5, max = 50.0
Element prediction: Off
Number of isotope peaks used for i-FIT = 3
Monoisotopic Mass, Even Electron Ions
66 formula(e) evaluated with 1 results within limits (up to 50 best isotopic matches for each mass)
Elements Used:
C: 0-200 H: 0-60 O: 0-6 Na: 0-1

Minimum:	80.00						-1.5		
Maximum:	100.00			2.0	5.0		50.0		
Mass	RA	Calc.Mass	mDa	PPM	DBE	i-FIT	Norm	Conf(%)	Formula
375.1784	100.00	375.1784	0.0	0.0	5.5	745.9	n/a	n/a	$C_{19}H_{28}O_6Na$

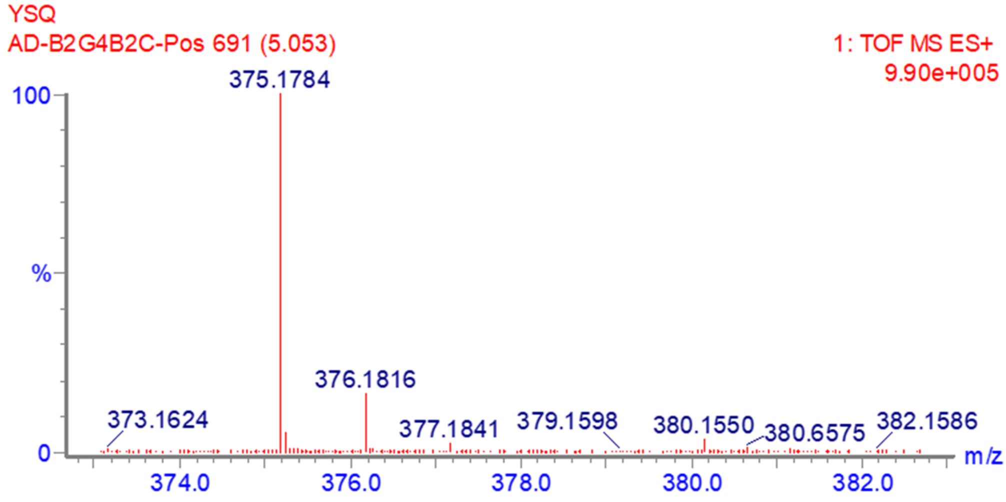

Figure S21 HR-ESIMS spectrum of compound **3**

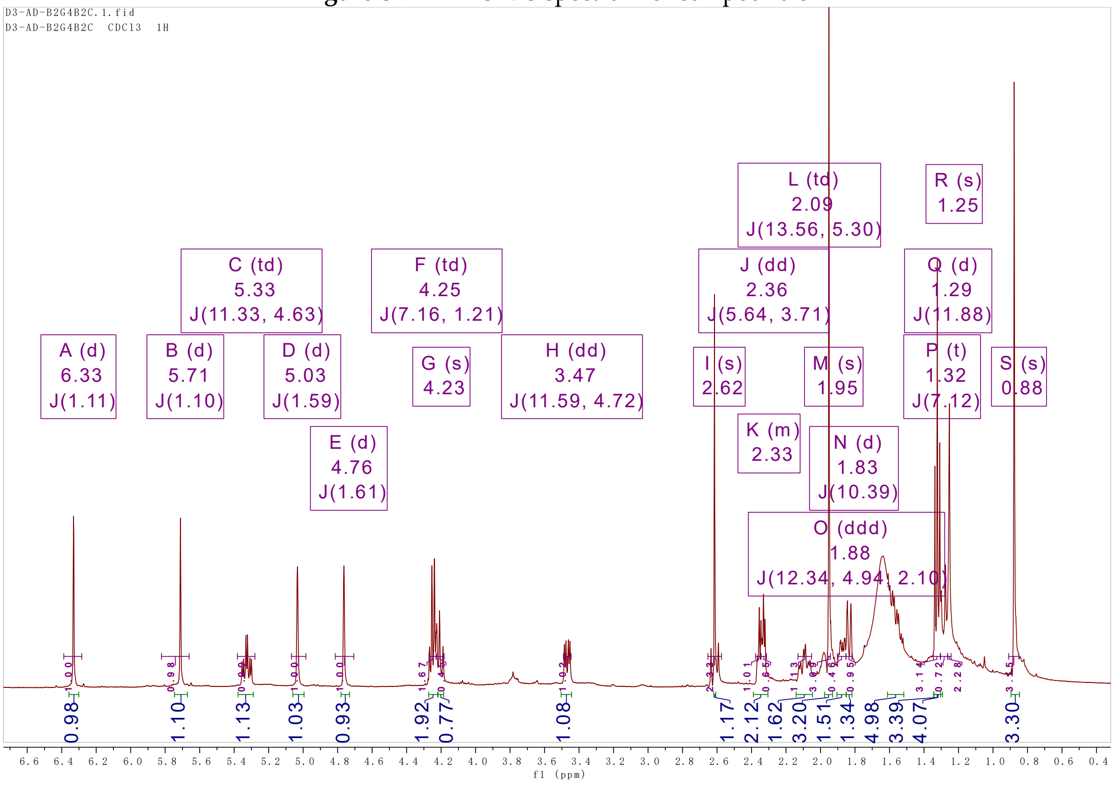

Figure S22 ^{1}H NMR spectrum of compound **3**

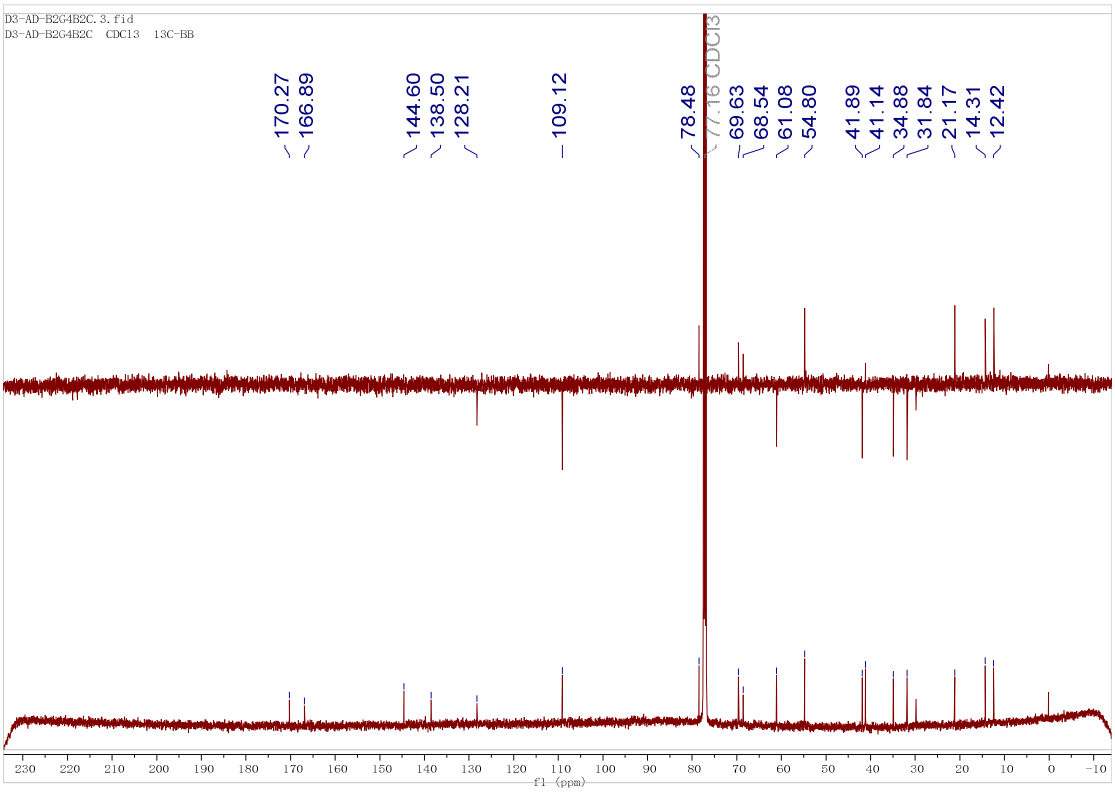

Figure S23 ^{13}C NMR spectrum of compound **3**

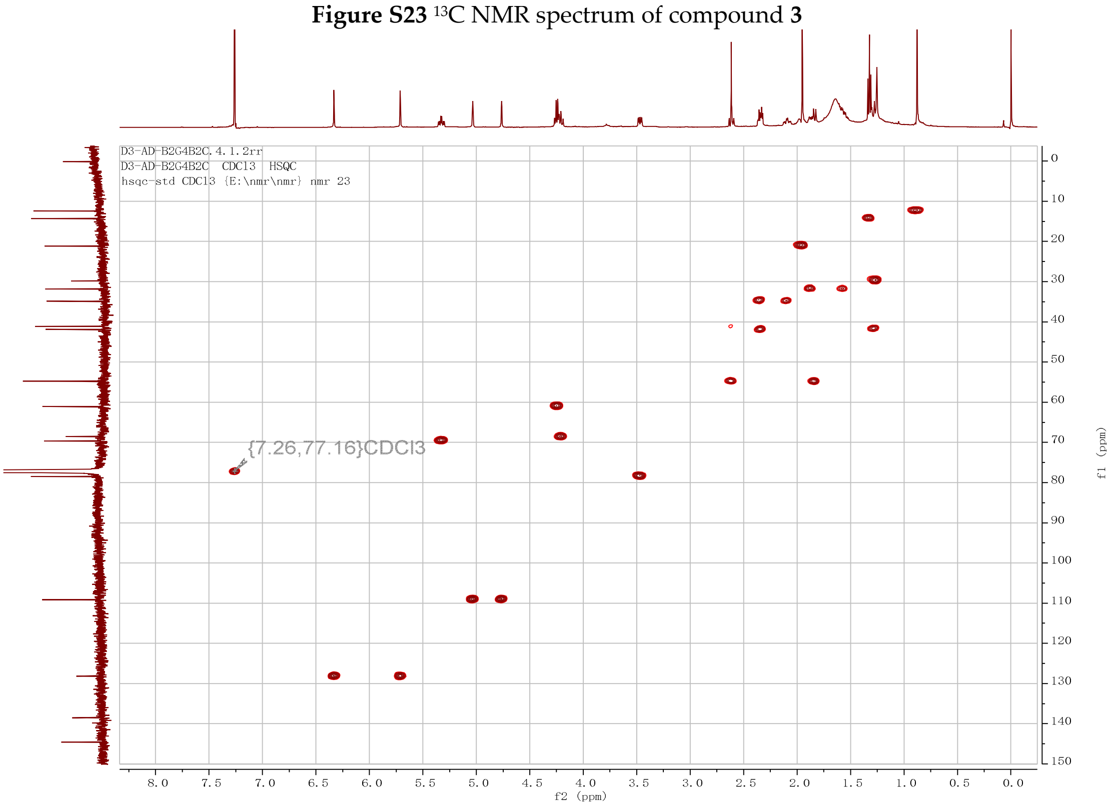

Figure S24 HSQC NMR spectrum of compound **3**

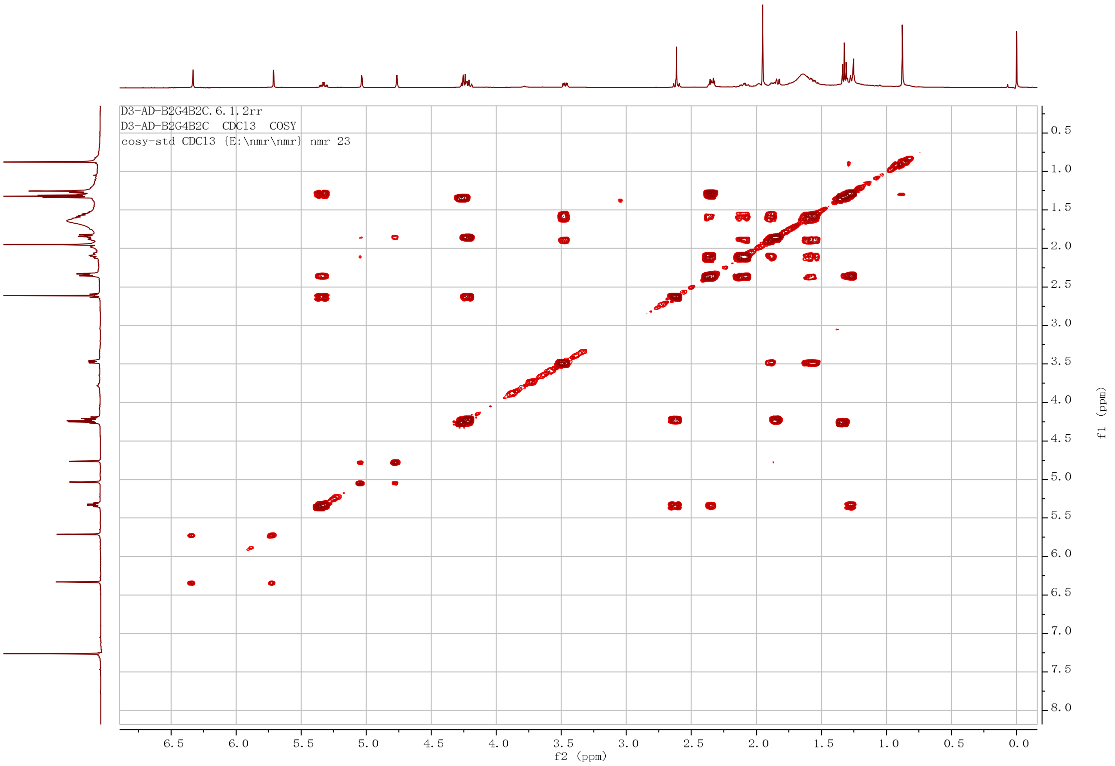

Figure S25 ^{1}H-^{1}H COSY spectrum of compound **3**

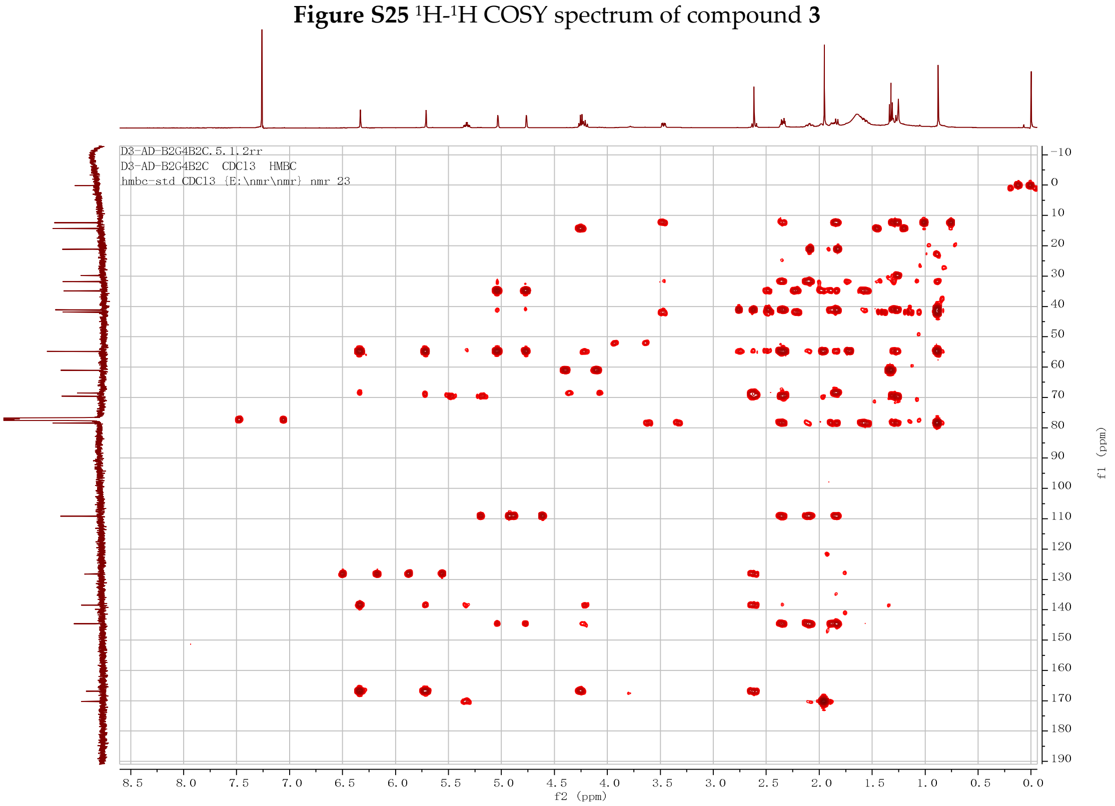

Figure S26 HMBC spectrum of compound **3**

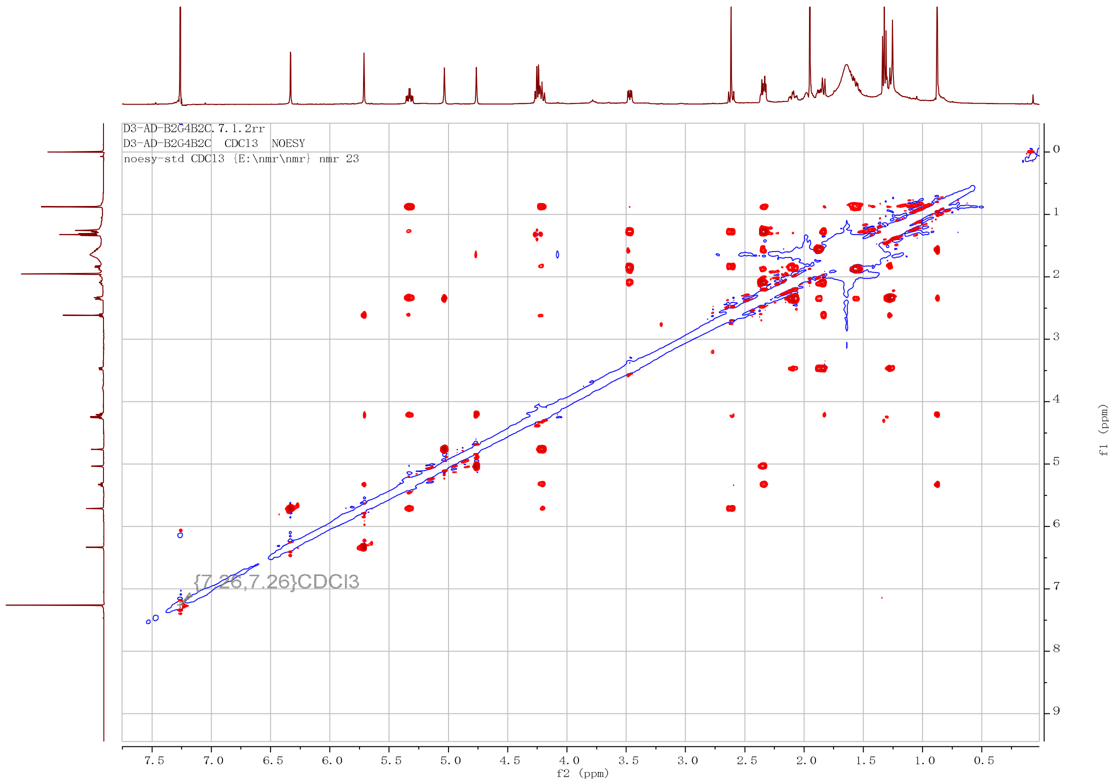

Figure S27 NOESY spectrum of compound **3**

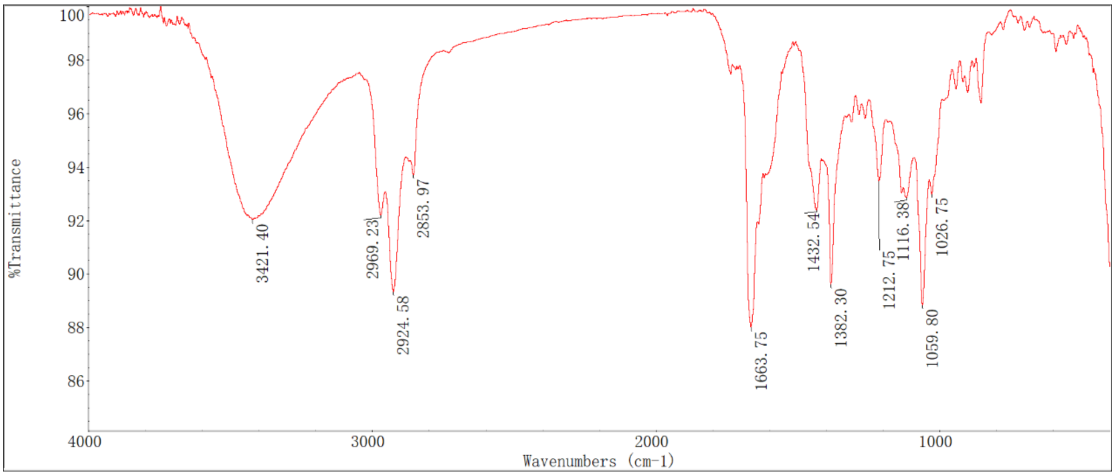

Figure S28 IR spectrum of compound **3**

Qualitative Analysis Report

**Data Filename**	ESIH202301003.d	**Sample Name**	D3-D3-AD-B2G7C2B2B
**Sample ID**		**Position**	P1-A3
**Instrument Name**	Agilent G6520 Q-TOF	**Acq Method**	20160322_MS_ESIH_POS_1min.m
**Acquired Time**	2/17/2023 10:25:17	**IRM Calibration Status**	Success
**DA Method**	small molecular data analysis method.m	**Comment**	ESIH by fangsu

User Spectra

Fragmentor Voltage	Collision Energy	Ionization Mode
175	0	ESI

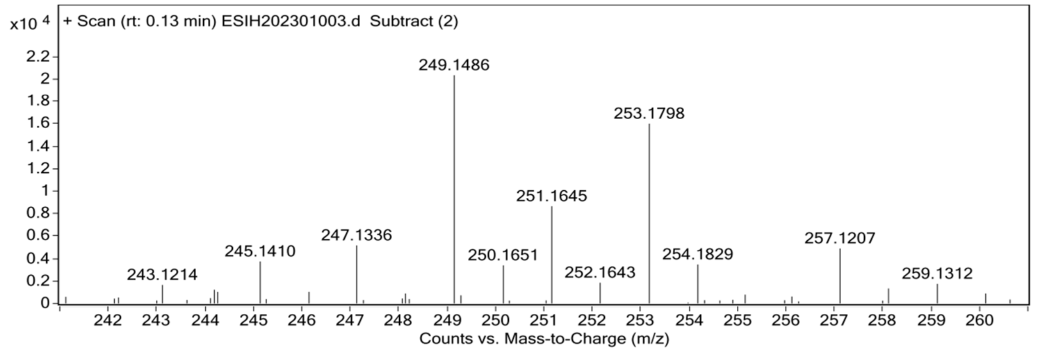

Formula Calculator Results

m/z	Calc m/z	Diff (mDa)	Diff (ppm)	Ion Formula	Ion
249.1486	249.1485	-0.07	-0.29	C15 H21 O3	(M+H)+

Figure S29 HR-ESIMS spectrum of compound **4**

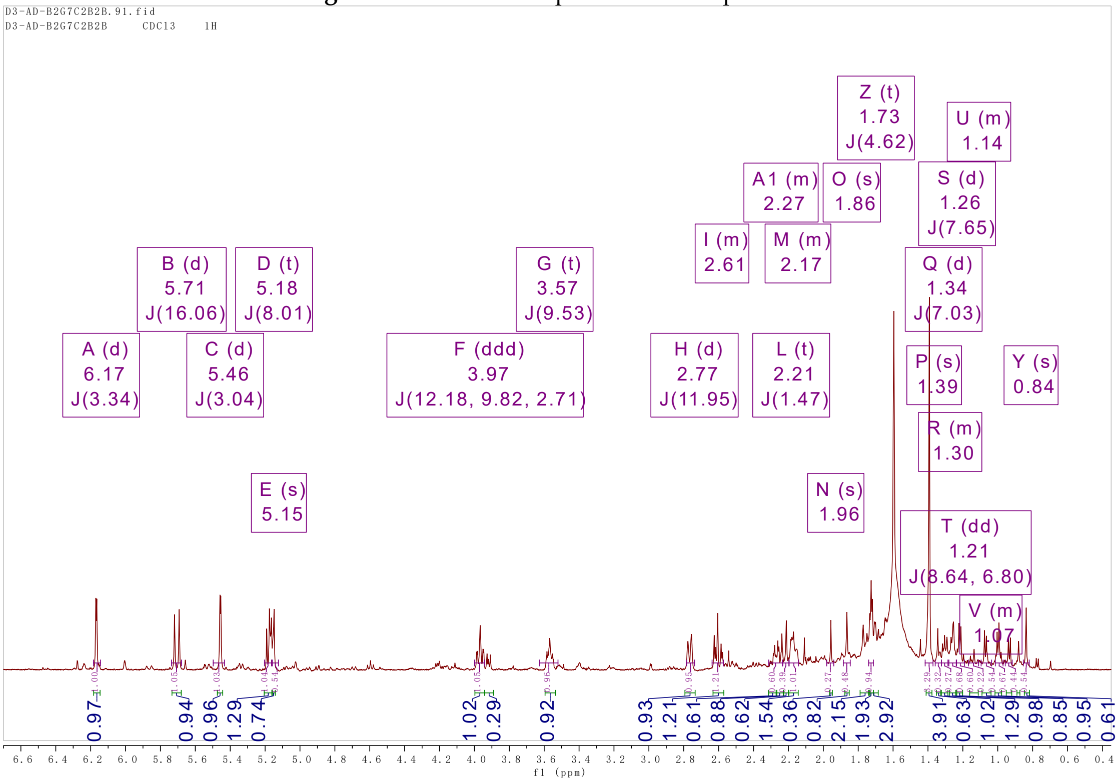

Figure S30 ^{1}H NMR spectrum of compound **4**

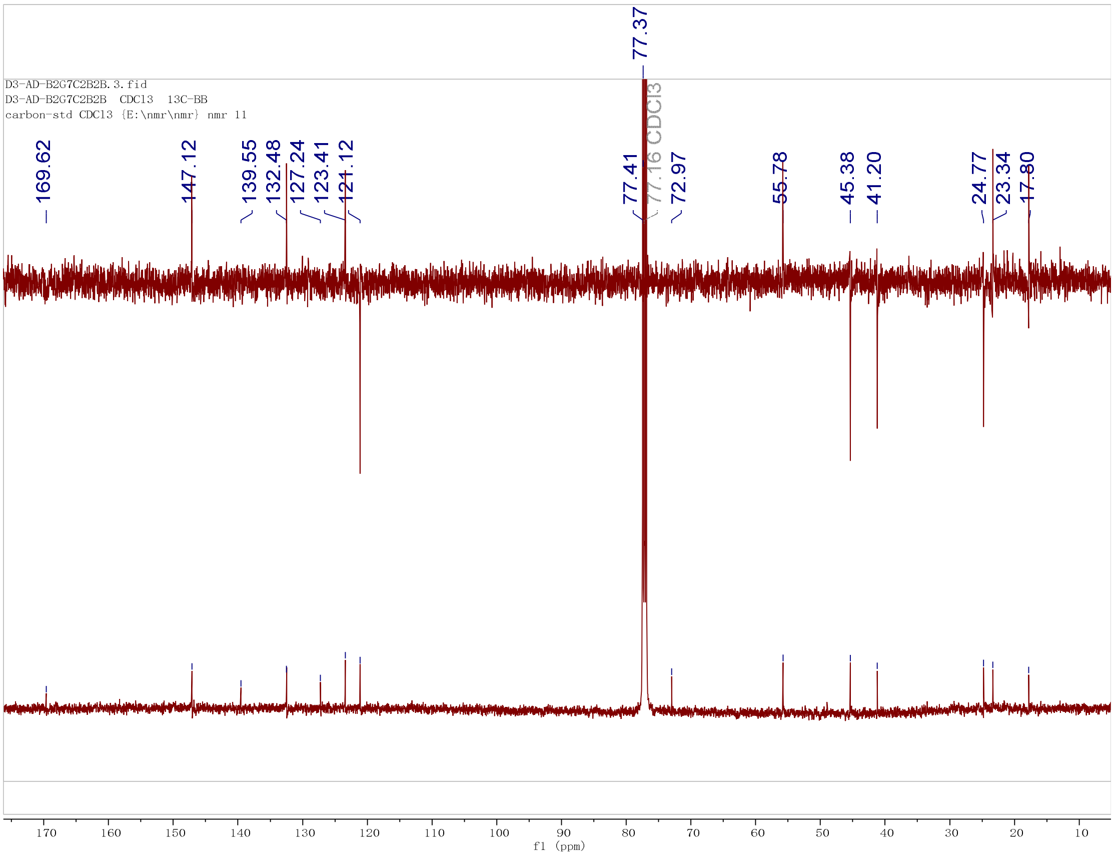

Figure S31 ^{13}C NMR spectrum of compound **4**

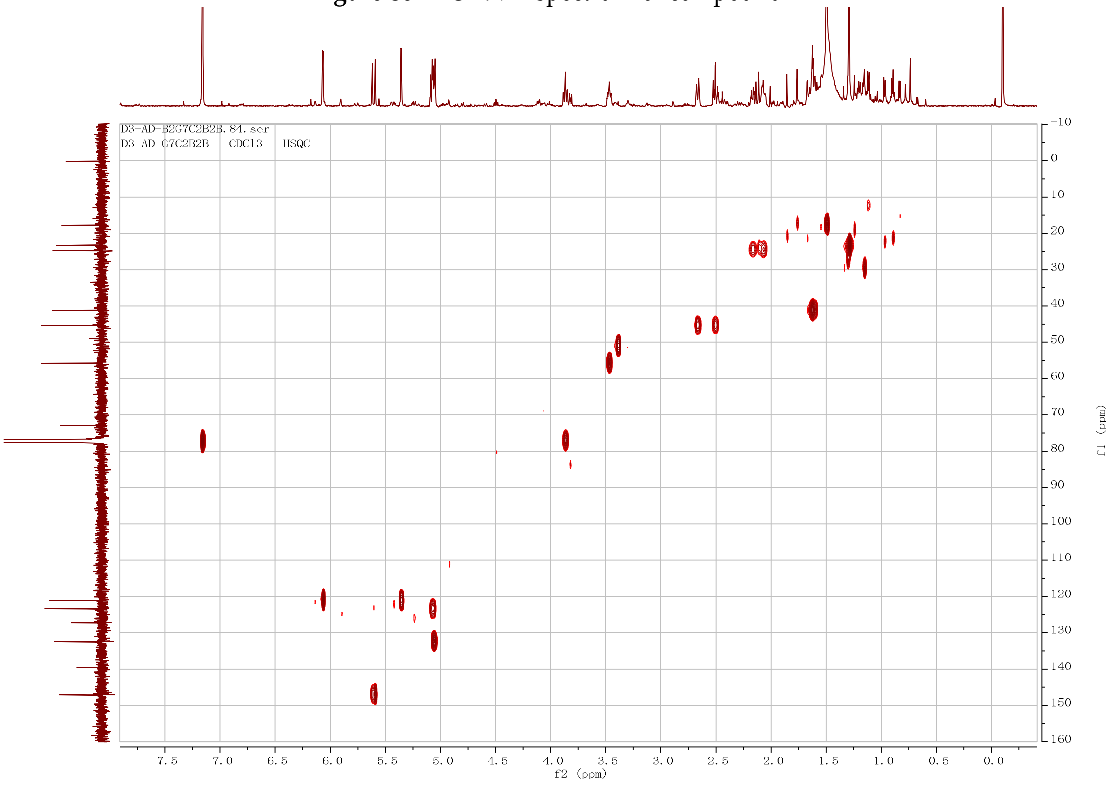

Figure S32 HSQC spectrum of compound **4**

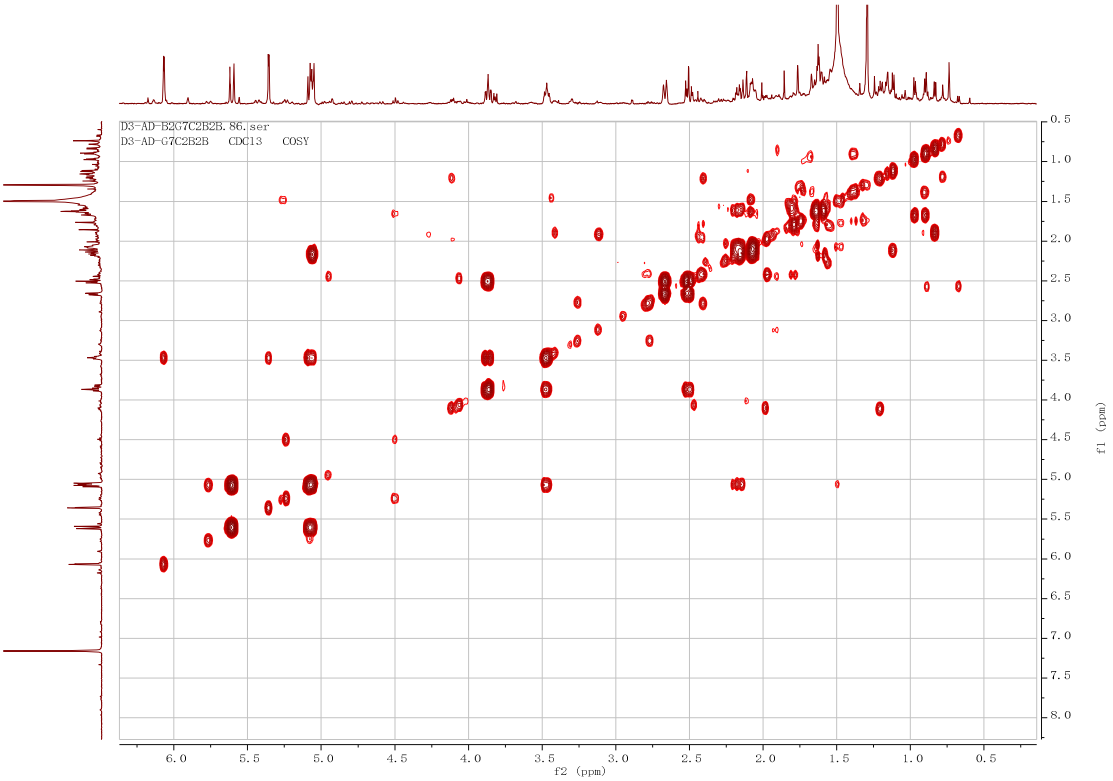

Figure S33 ^{1}H-^{1}H COSY spectrum of compound **4**

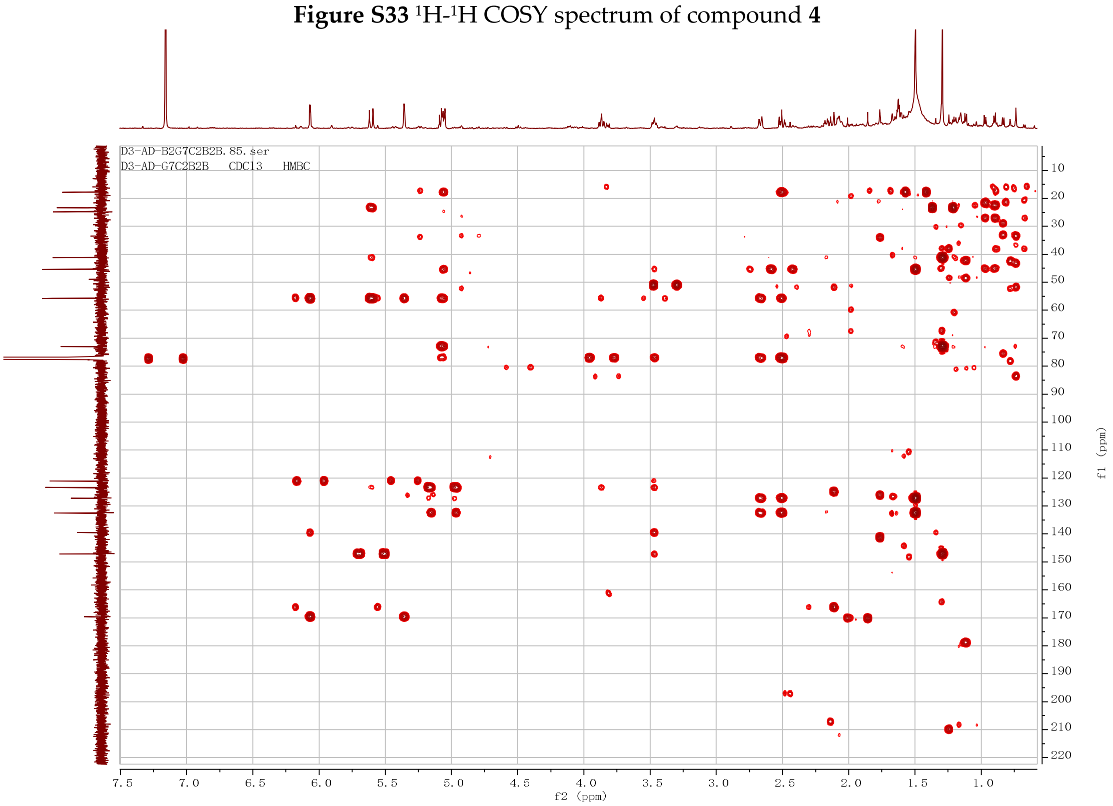

Figure S34 HMBC spectrum of compound **4**

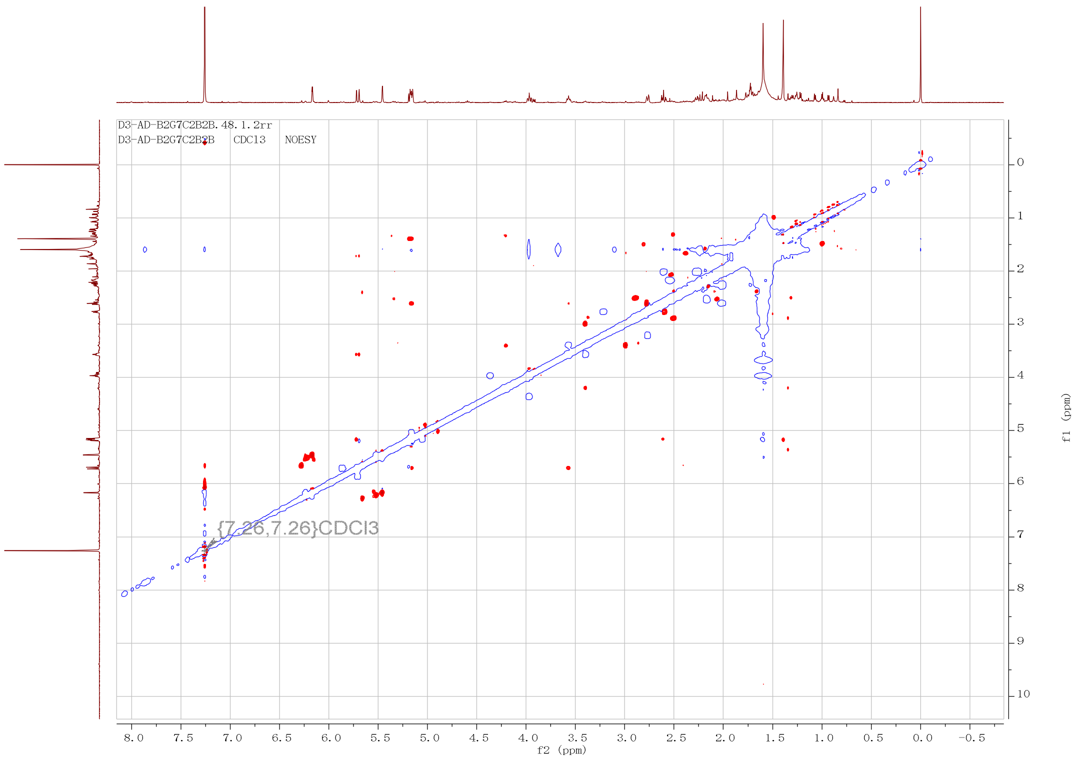

Figure S35 NOESY spectrum of compound **4**

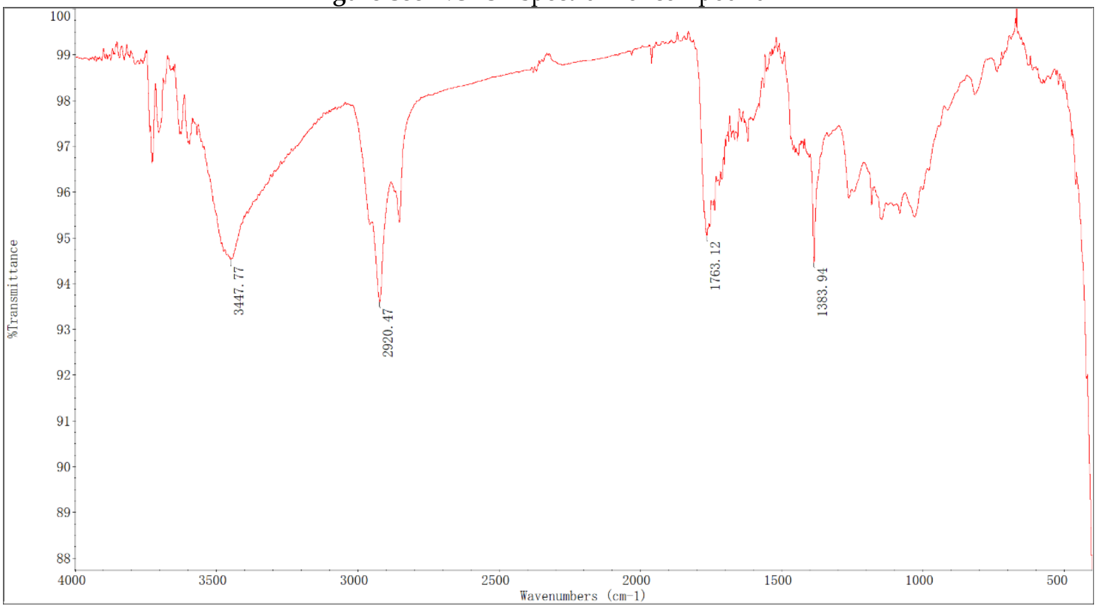

Figure S36 IR spectrum of compound **4**

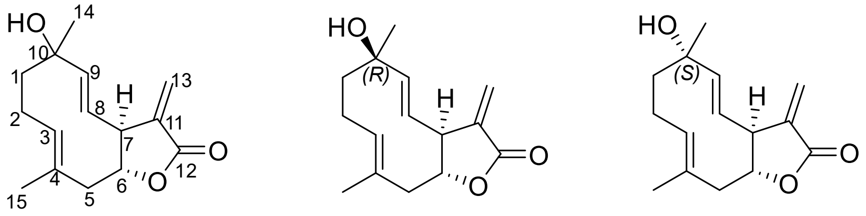

4 **10R** **10S**

Figure S37 Possible isomers of compound **4**

Functional	Solvent?	Basis Set	
mPW1PW91	PCM	6-311G(d, p)	

	Isomer 1	Isomer 2	Isomer 3	Isom
sDP4+ (H data)	1.67%	98.33%	–	–
sDP4+ (C data)	0.02%	99.98%	–	–
sDP4+ (all data)	0.00%	100.00%	–	–
uDP4+ (H data)	9.94%	90.06%	–	–
uDP4+ (C data)	0.08%	99.92%	–	–
uDP4+ (all data)	0.01%	99.99%	–	–
DP4+ (H data)	0.19%	99.81%	–	–
DP4+ (C data)	0.00%	100.00%	–	–
DP4+ (all data)	0.00%	100.00%	–	–

Figure S38 DP4+ probability statistics of compound **4**

Qualitative Analysis Report

**Data Filename**	ESIH202301002.d	**Sample Name**	D3-D3-AD-B2G6C2A1
**Sample ID**		**Position**	P1-A2
**Instrument Name**	Agilent G6520 Q-TOF	**Acq Method**	20160322_MS_ESIH_POS_1min.m
**Acquired Time**	2/17/2023 10:24:00	**IRM Calibration Status**	Success
**DA Method**	small molecular data analysis method.m	**Comment**	ESIH by fangsu

User Spectra

Fragmentor Voltage	Collision Energy	Ionization Mode
175	0	ESI

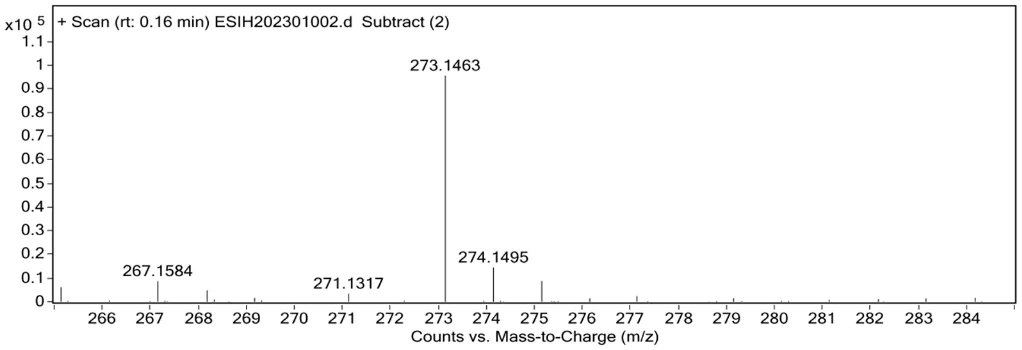

Formula Calculator Results

m/z	Calc m/z	Diff (mDa)	Diff (ppm)	Ion Formula	Ion
273.1463	273.1461	-0.17	-0.62	C15 H22 Na O3	(M+Na)+

Figure S39 HR-ESIMS spectrum of compound **5**

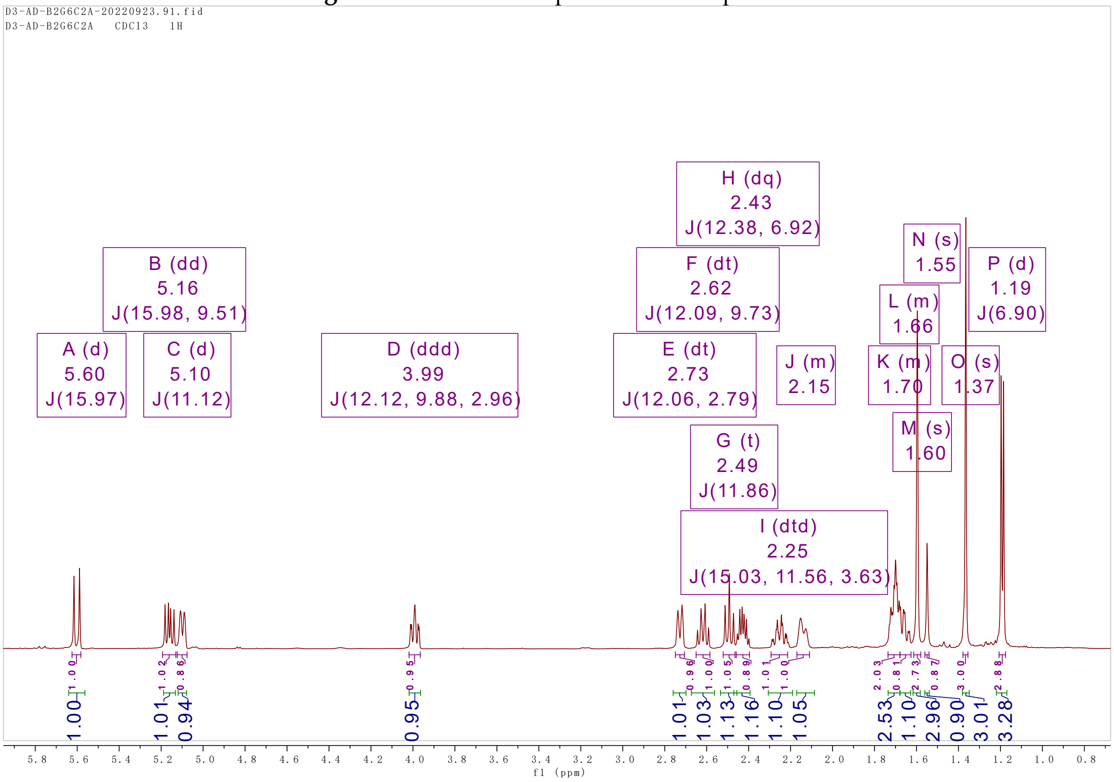

Figure S40 ^{1}H NMR spectrum of compound **5**

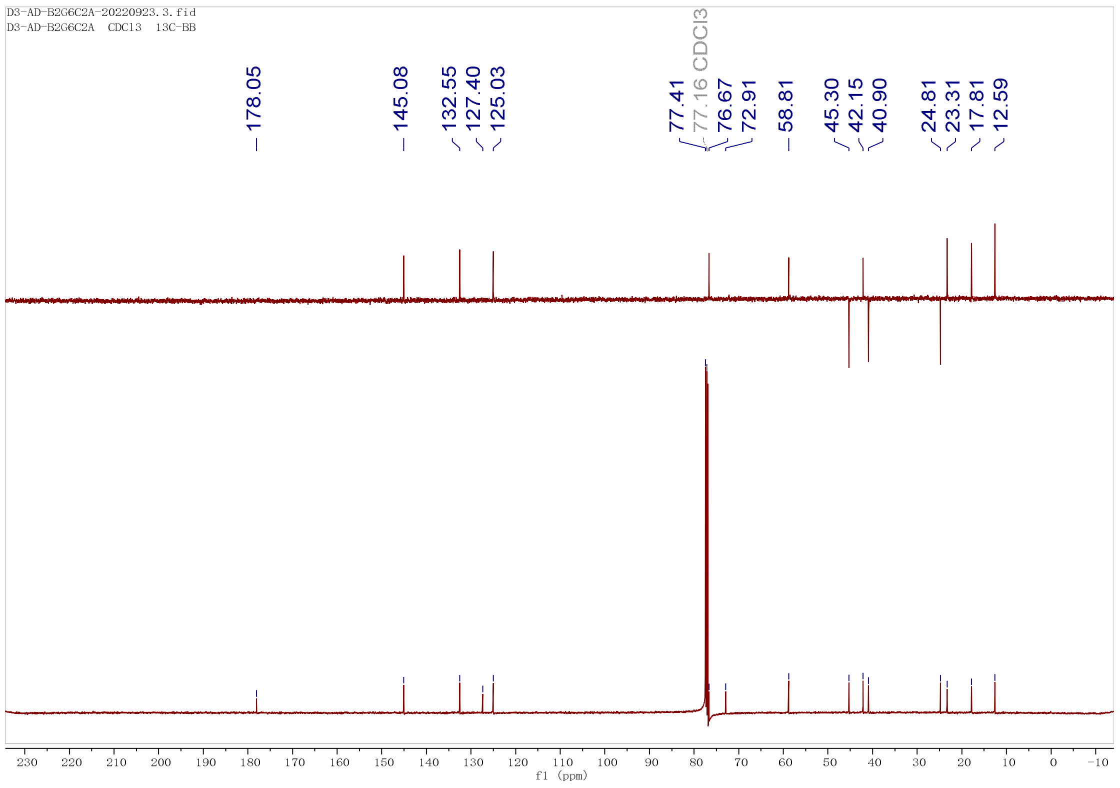

Figure S41 ^{13}C NMR spectrum of compound **5**

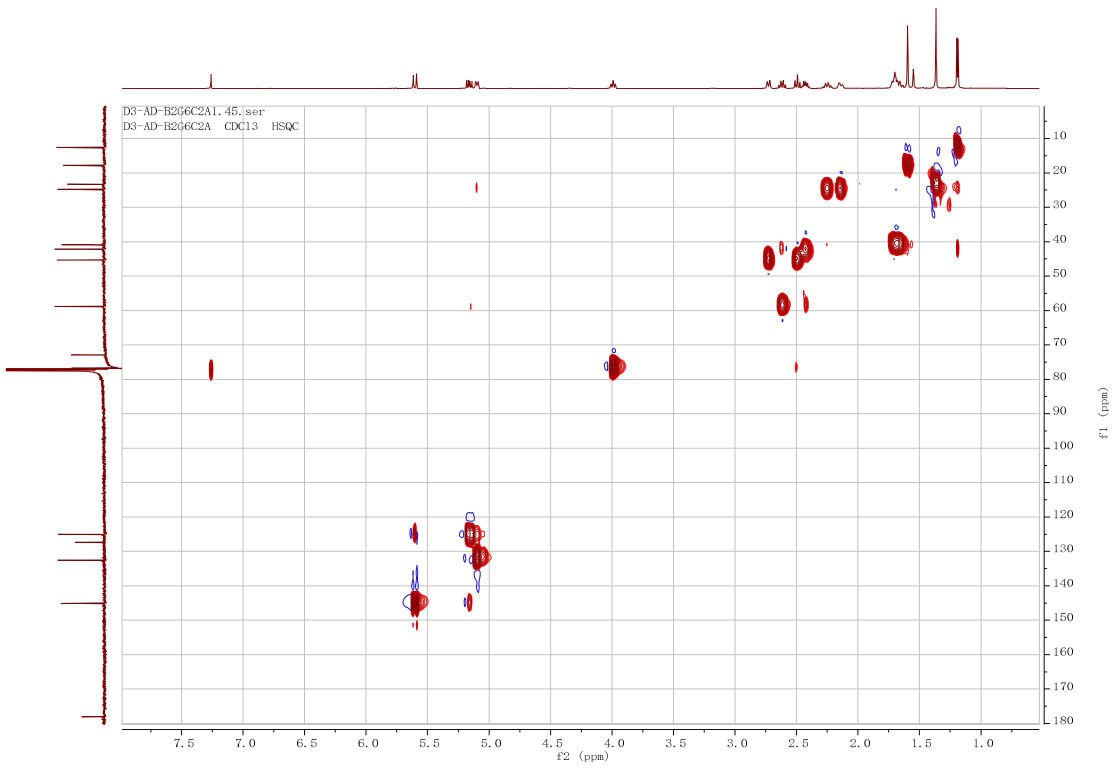

Figure S42 HSQC spectrum of compound **5**

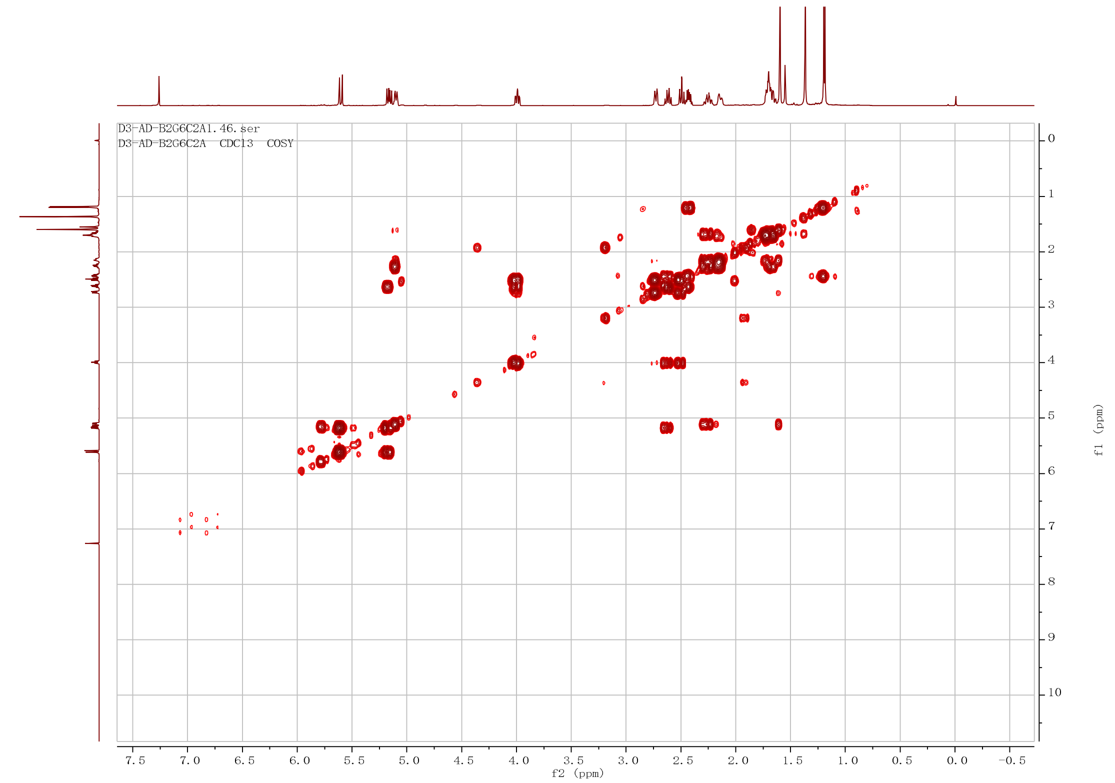

Figure S43 ^{1}H-^{1}H COSY spectrum of compound **5**

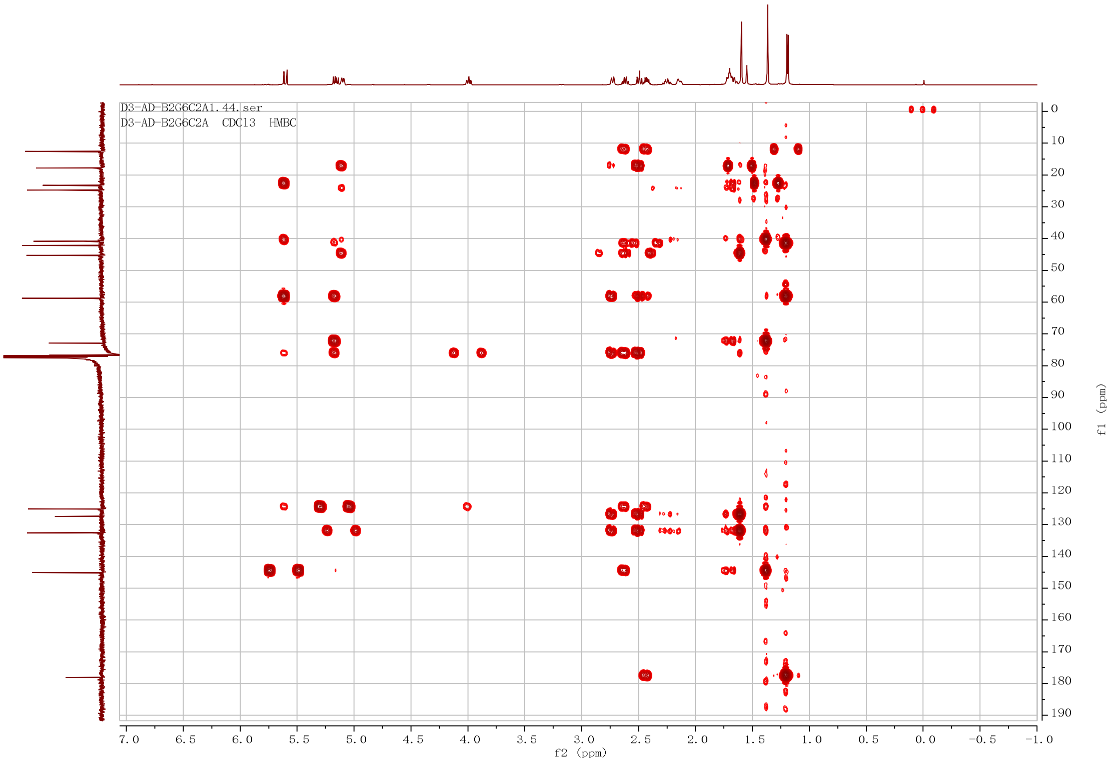

Figure S44 HMBC spectrum of compound **5**

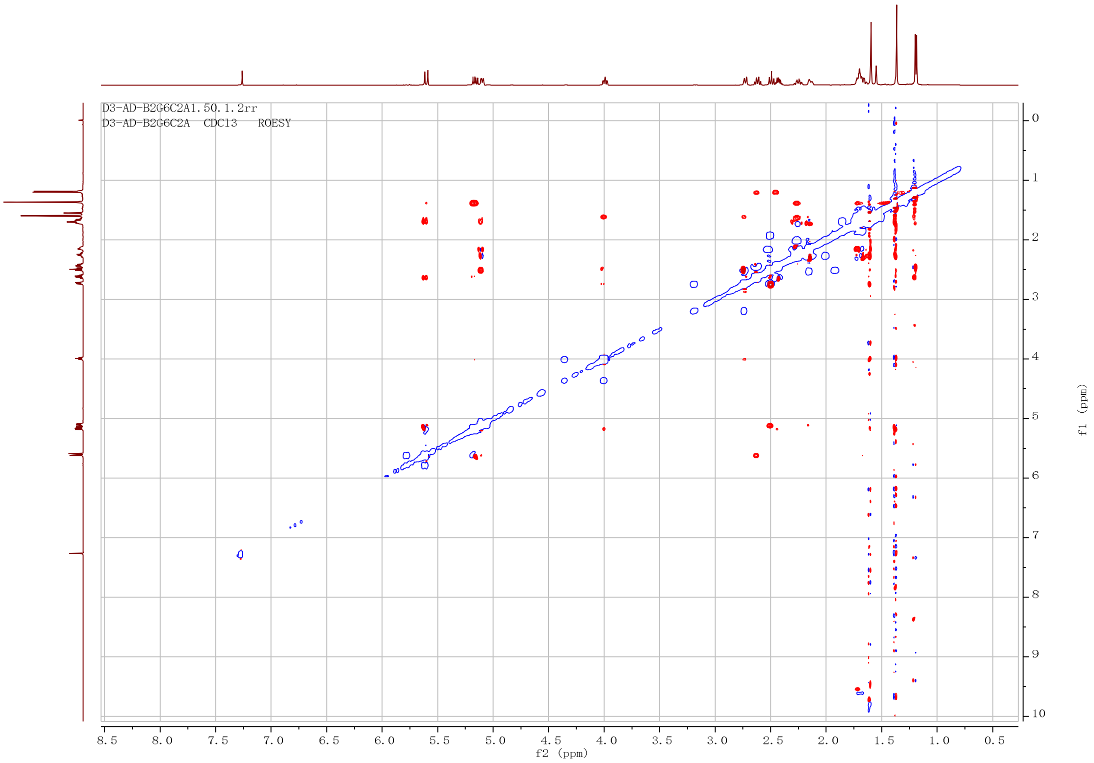

Figure S45 NOESY spectrum of compound **5**

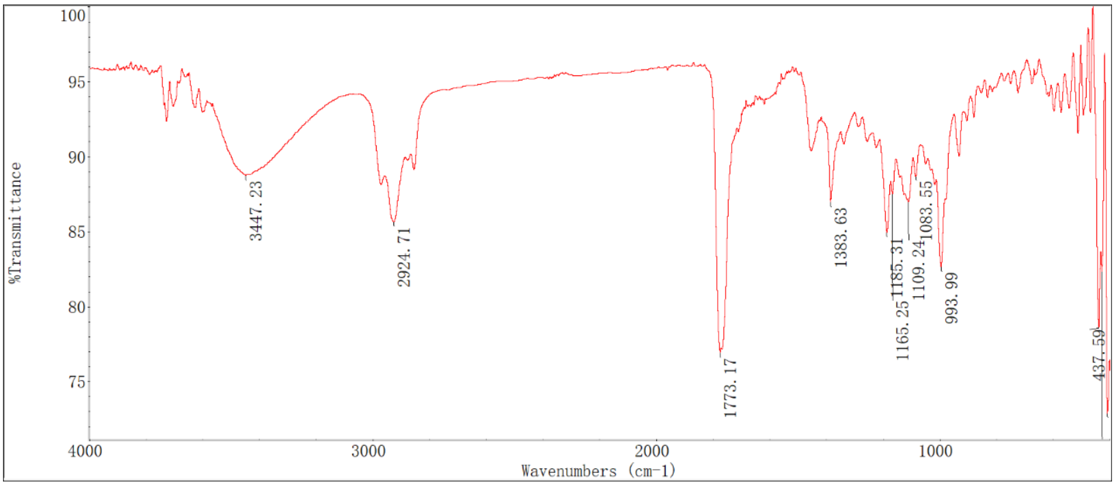

Figure S46 IR spectrum of compound **5**

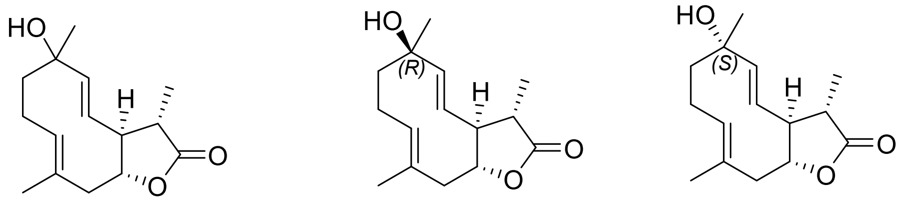

5 **10R** **10S**

Figure S47 Possible isomers of compound **5**

Functional	Solvent?	Basis Set
mPW1PW91	PCM	6-311G(d, p)

	Isomer 1	Isomer 2	Isomer 3	Isom
sDP4+ (H data)	0.85%	99.15%	-	-
sDP4+ (C data)	3.75%	96.25%	-	-
sDP4+ (all data)	0.03%	99.97%	-	-
uDP4+ (H data)	0.20%	99.80%	-	-
uDP4+ (C data)	18.59%	81.41%	-	-
uDP4+ (all data)	0.05%	99.95%	-	-
DP4+ (H data)	0.00%	100.00%	-	-
DP4+ (C data)	0.88%	99.12%	-	-
DP4+ (all data)	0.00%	100.00%	-	-

Figure S48 DP4+ probability statistics of compound **5**

Elemental Composition Report
Tolerance = 5.0 PPM / DBE: min = -1.5, max = 50.0
Element prediction: Off
Number of isotope peaks used for i-FIT = 3
Monoisotopic Mass, Even Electron Ions
41 formula(e) evaluated with 1 results within limits (up to 50 best isotopic matches for each mass)
Elements Used:
C: 0-200 H: 0-60 O: 0-6 Na: 0-1

Minimum:	80.00				-1.5				
Maximum:	100.00		2.0	5.0	50.0				
Mass	RA	Calc. Mass	mDa	PPM	DBE	i-FIT	Norm	Conf(%)	Formula
219.1744	00.00	219.1749	-0.5	-2.3	4.5	424.1	n/a	n/a	$C_{15}H_{23}O$

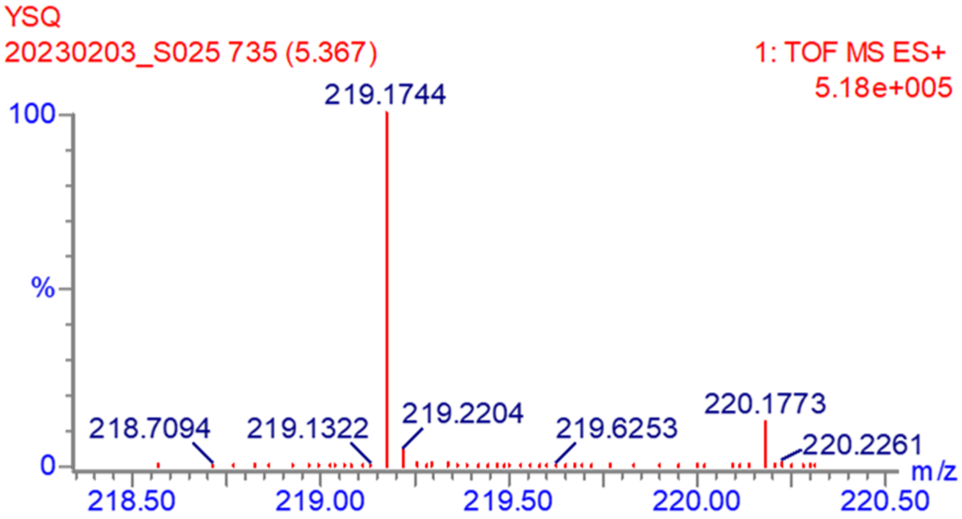

Figure S49 HR-ESIMS spectrum of compound **6**

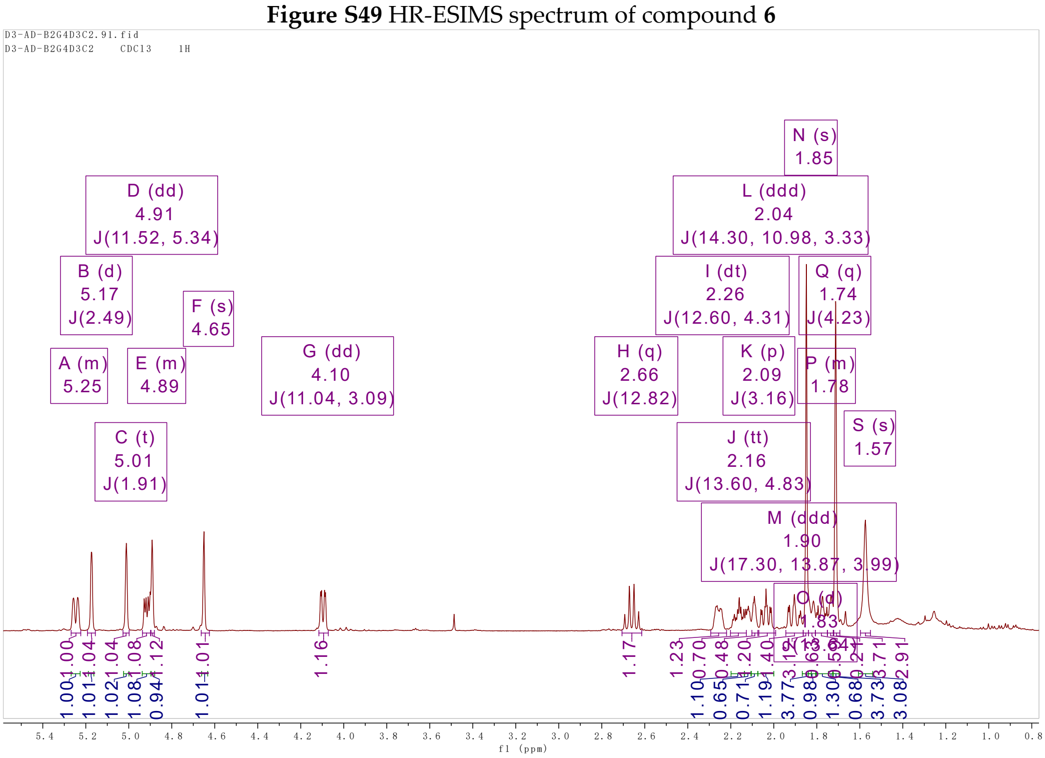

Figure S50 ^{1}H NMR spectrum of compound **6**

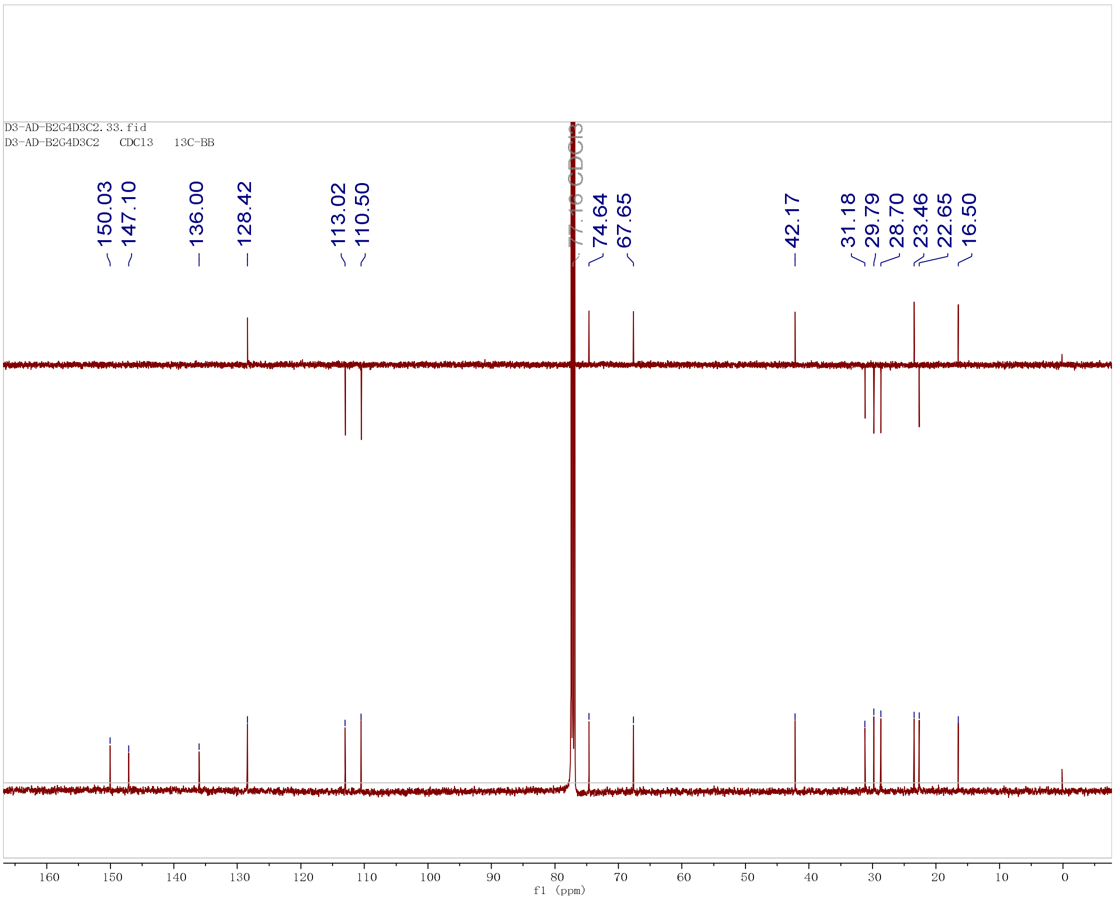

Figure S51 ^{13}C NMR spectrum of compound **6**

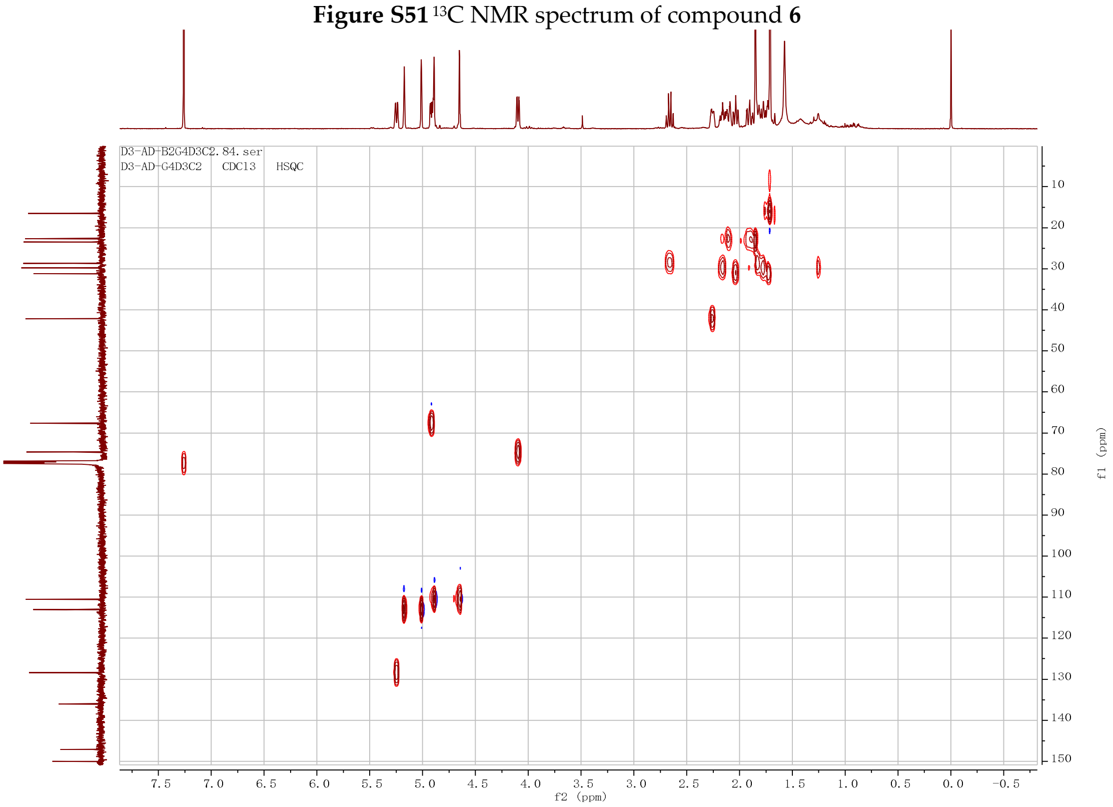

Figure S52 HSQC spectrum of compound **6**

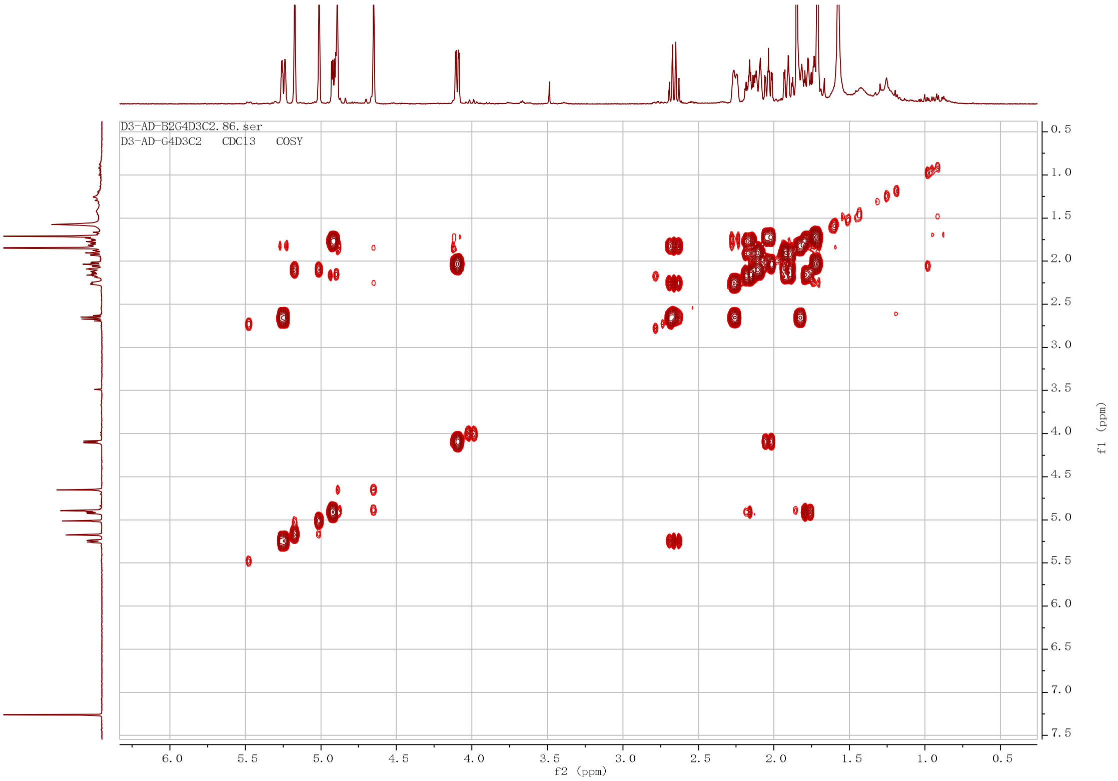

Figure S53 ^{1}H-^{1}H COSY spectrum of compound **6**

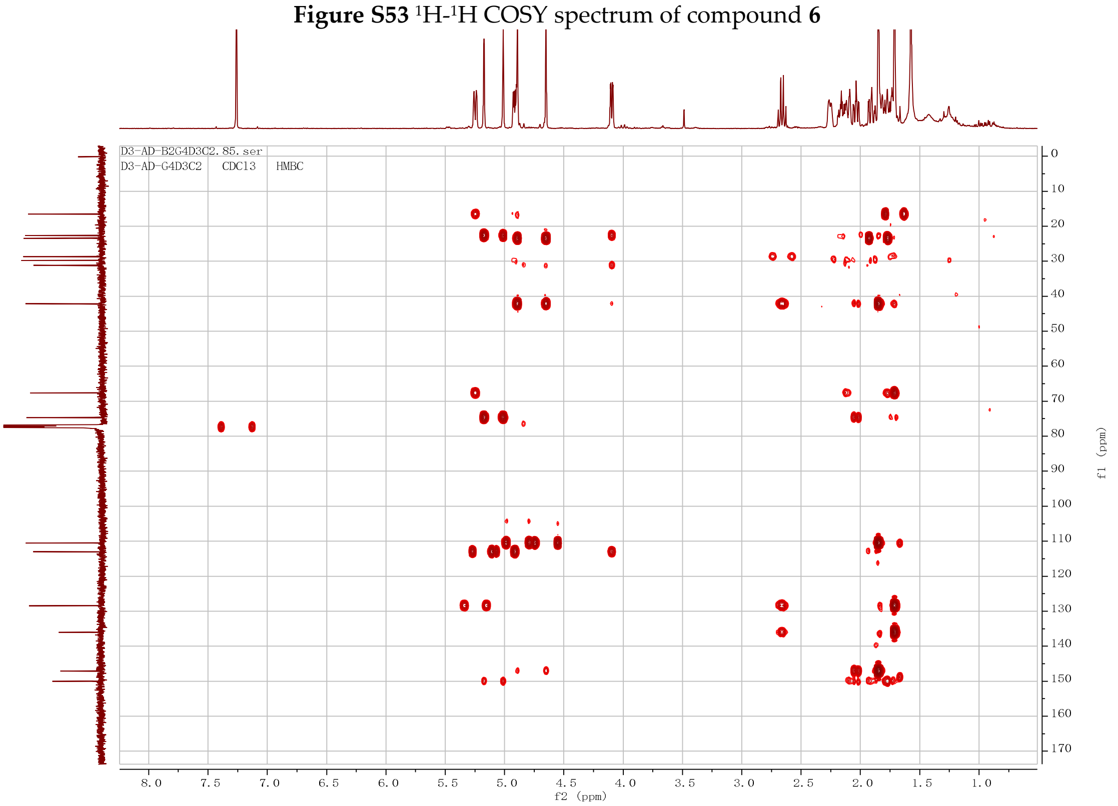

Figure S54 HMBC spectrum of compound **6**

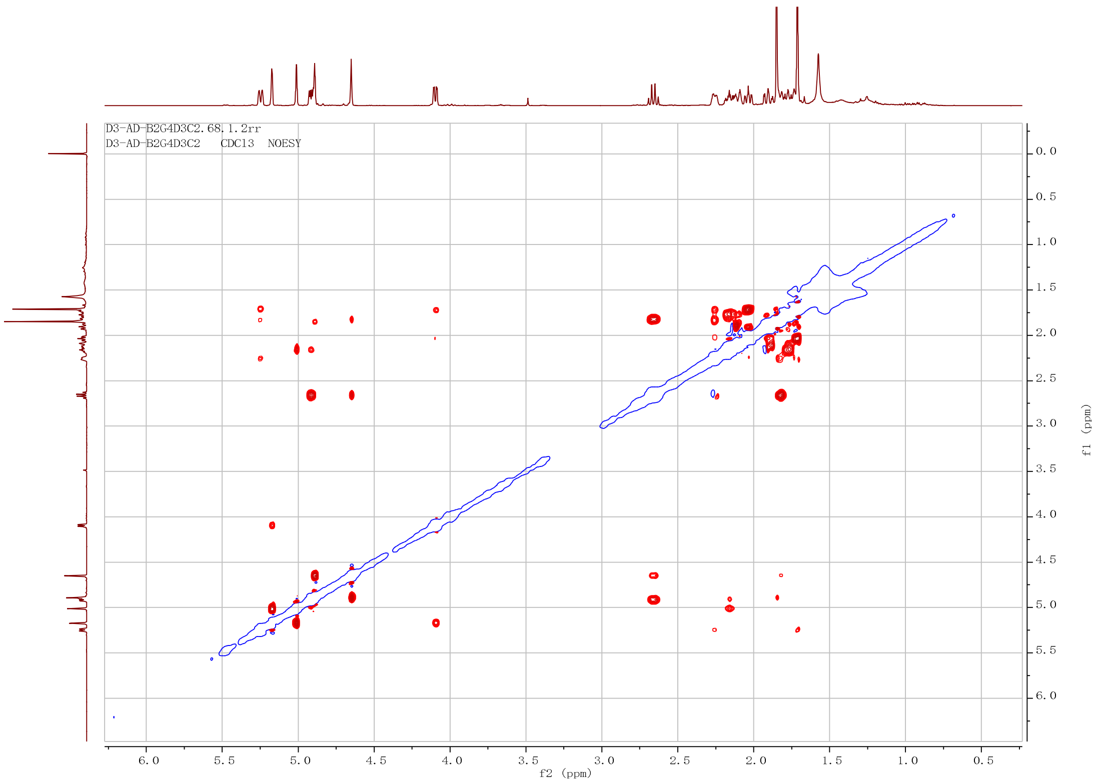

Figure S55 NOESY spectrum of compound **6**

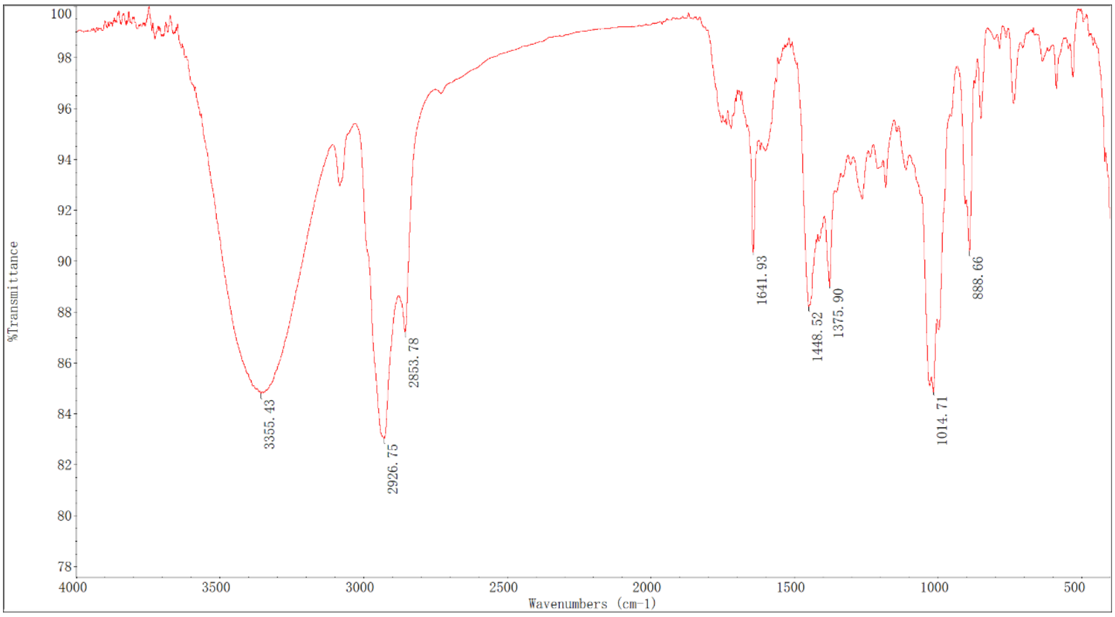

Figure S56 IR spectrum of compound **6**

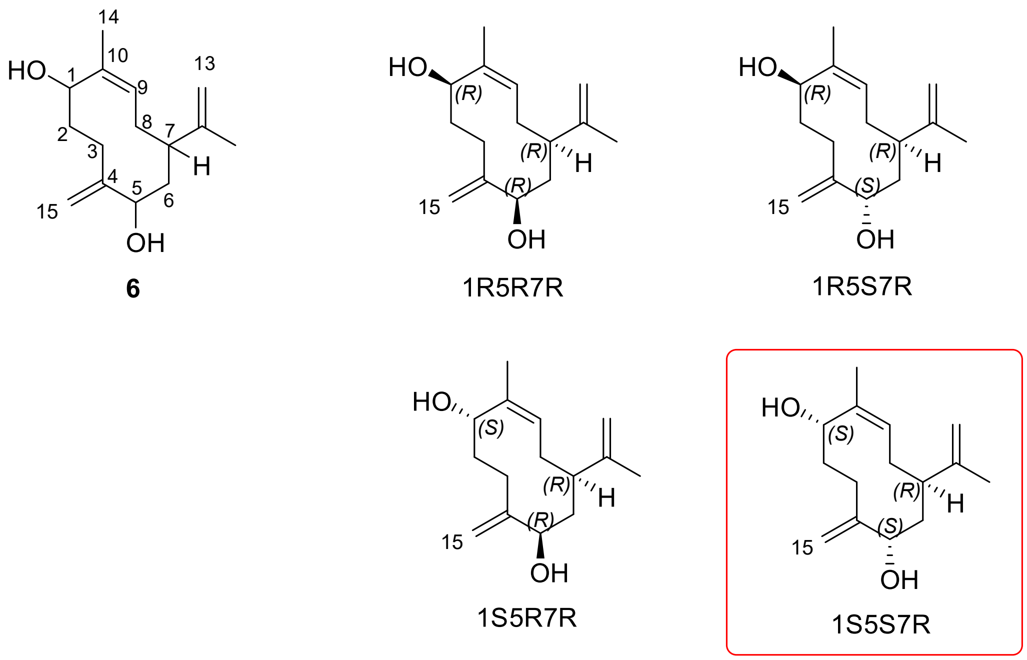

Figure S57 Possible isomers of compound **6**

Functional	Solvent?	Basis Set	
mPW1PW91	PCM	6-311G(d, p)	

	Isomer 1	Isomer 2	Isomer 3	Isomer 4
sDP4+ (H data)	1.36%	0.58%	0.00%	98.06%
sDP4+ (C data)	0.03%	0.00%	0.00%	99.97%
sDP4+ (all data)	0.00%	0.00%	0.00%	100.00%
uDP4+ (H data)	2.31%	0.00%	0.00%	97.69%
uDP4+ (C data)	0.04%	0.00%	0.00%	99.95%
uDP4+ (all data)	0.00%	0.00%	0.00%	100.00%
DP4+ (H data)	0.03%	0.00%	0.00%	99.97%
DP4+ (C data)	0.00%	0.00%	0.00%	100.00%
DP4+ (all data)	0.00%	0.00%	0.00%	100.00%

Figure S58 DP4+ probability statistics of compound **6**

Elemental Composition Report
Tolerance = 5.0 PPM / DBE: min = -1.5, max = 50.0
Element prediction: Off
Number of isotope peaks used for i-FIT = 3
Monoisotopic Mass, Even Electron Ions
22 formula(e) evaluated with 1 results within limits (up to 50 best isotopic matches for each mass)
Elements Used:
C: 0-200 H: 0-60 O: 0-6

Minimum:	80.00				-1.5				
Maximum:	100.00			2.0	5.0	50.0			
Mass	RA	Calc. Mass	mDa	PPM	DBE	i-FIT	Norm	Conf(%)	Formula
219.1743	100.00	219.1749	-0.6	-2.7	4.5	695.9	n/a	n/a	$C_{15}H_{23}O$

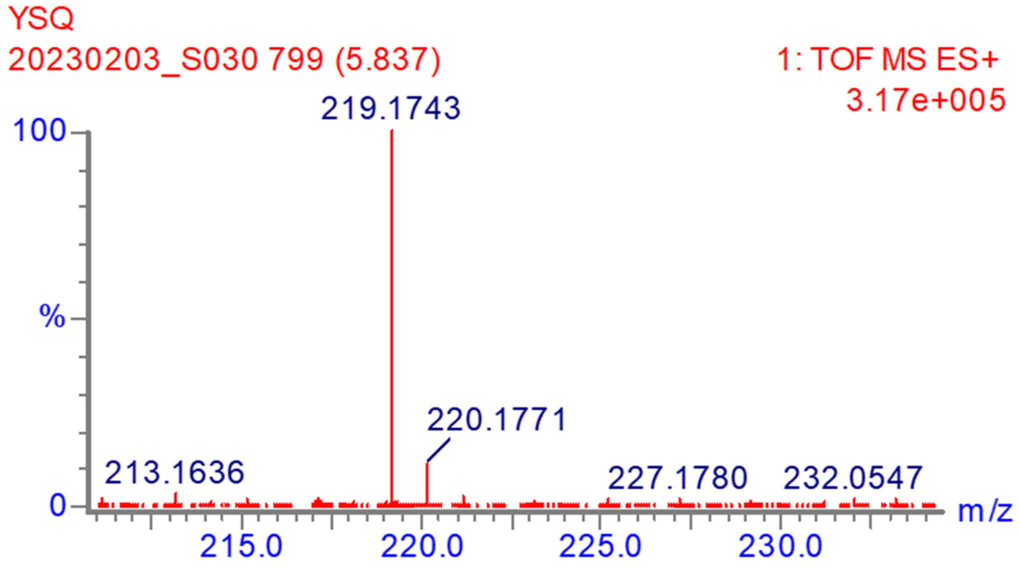

Figure S59 HR-ESIMS spectrum of compound **7**

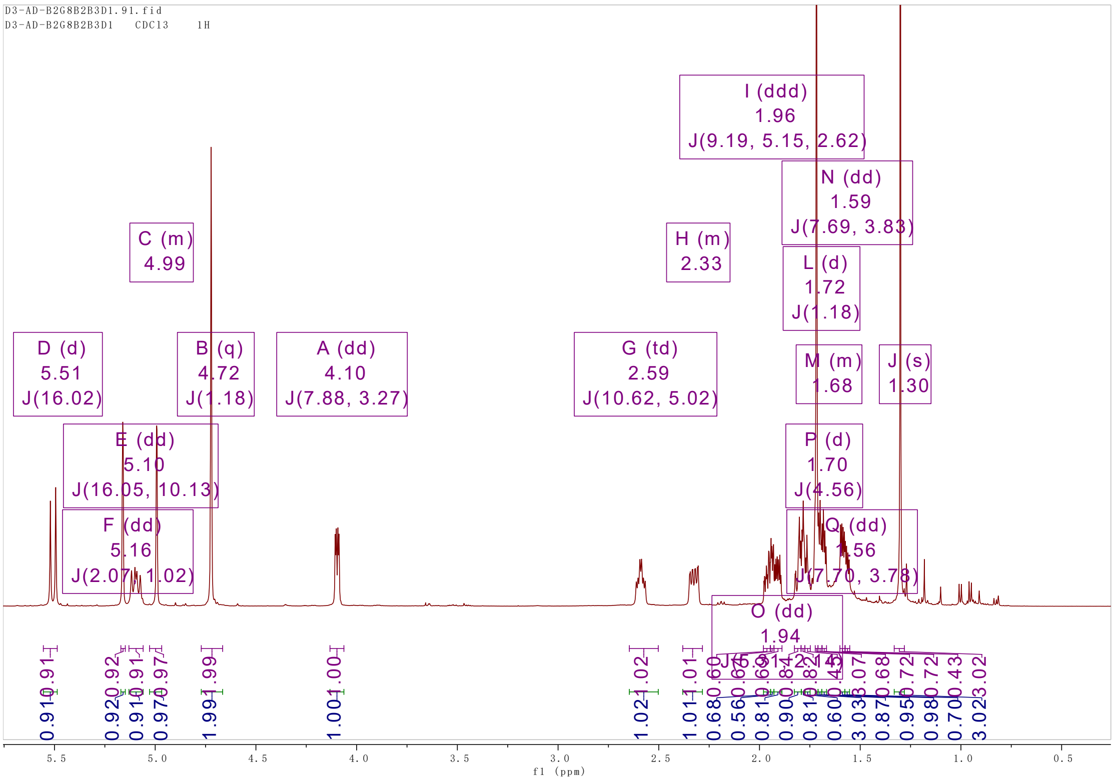

Figure S60 ^{1}H NMR spectrum of compound **7**

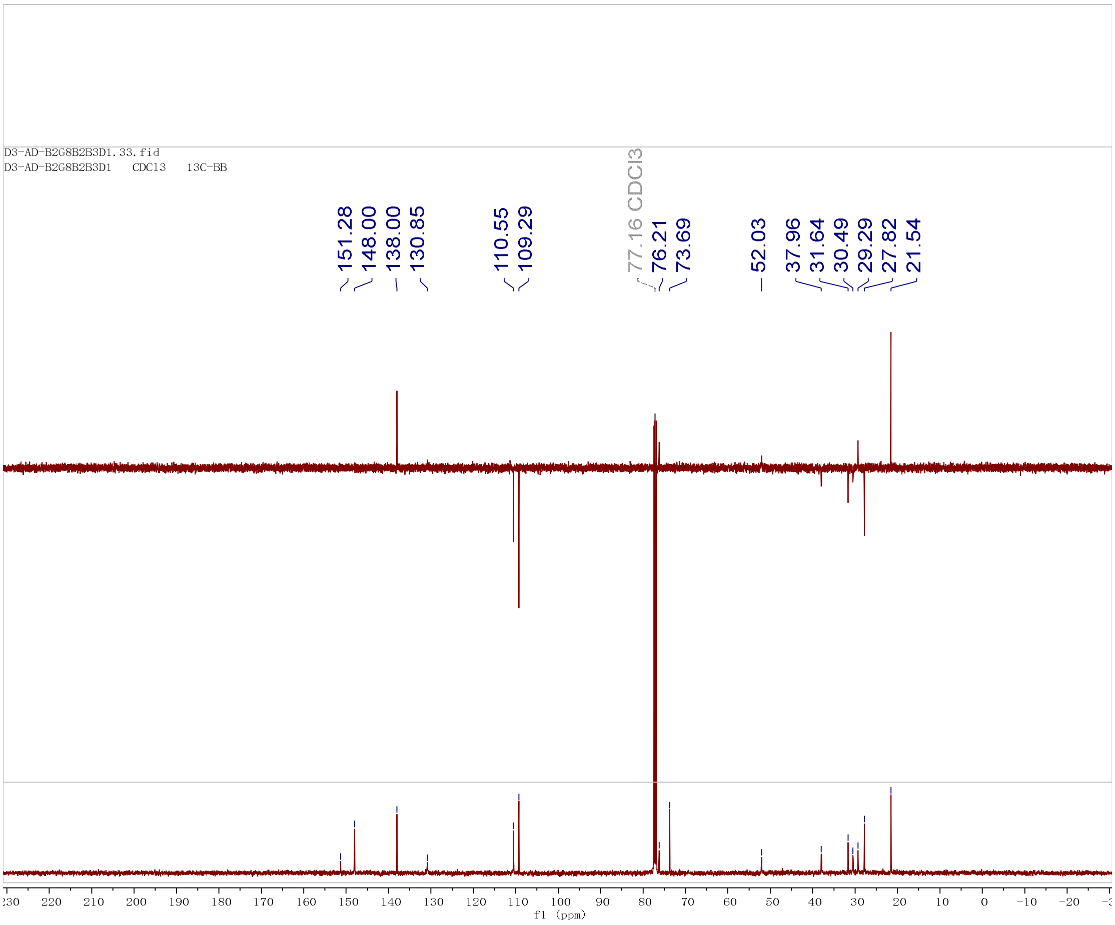

Figure S61 ^{13}C NMR spectrum of compound **7**

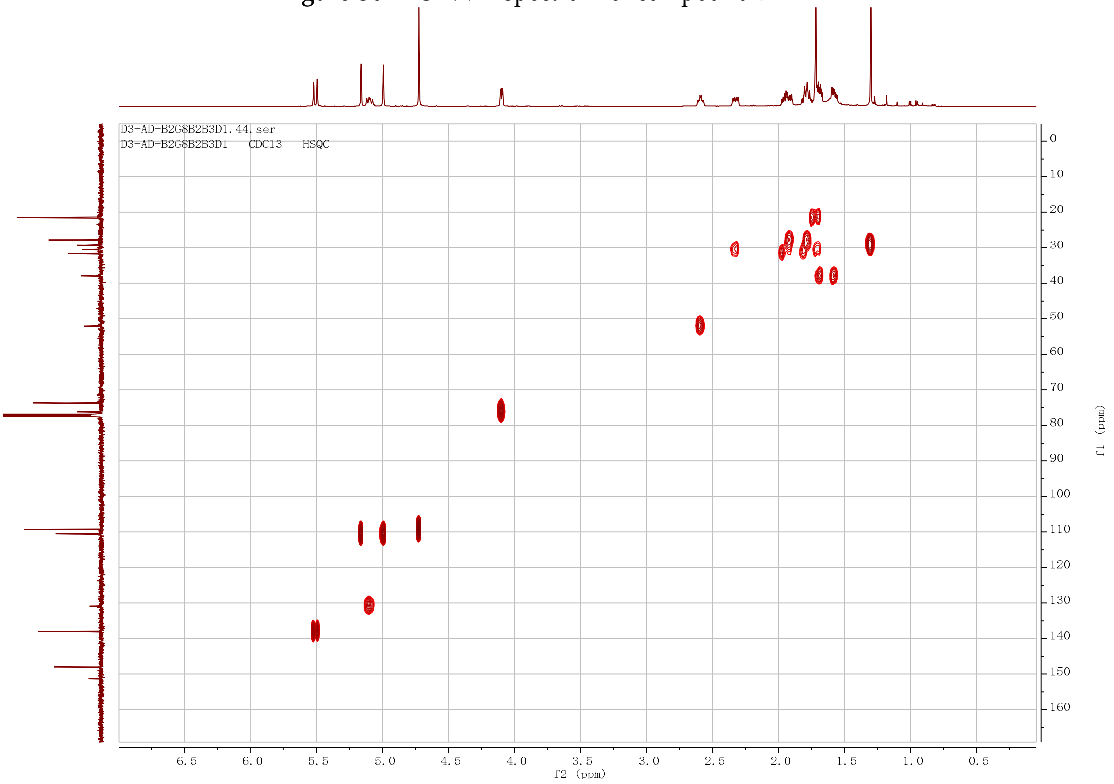

Figure S62 HSQC spectrum of compound **7**

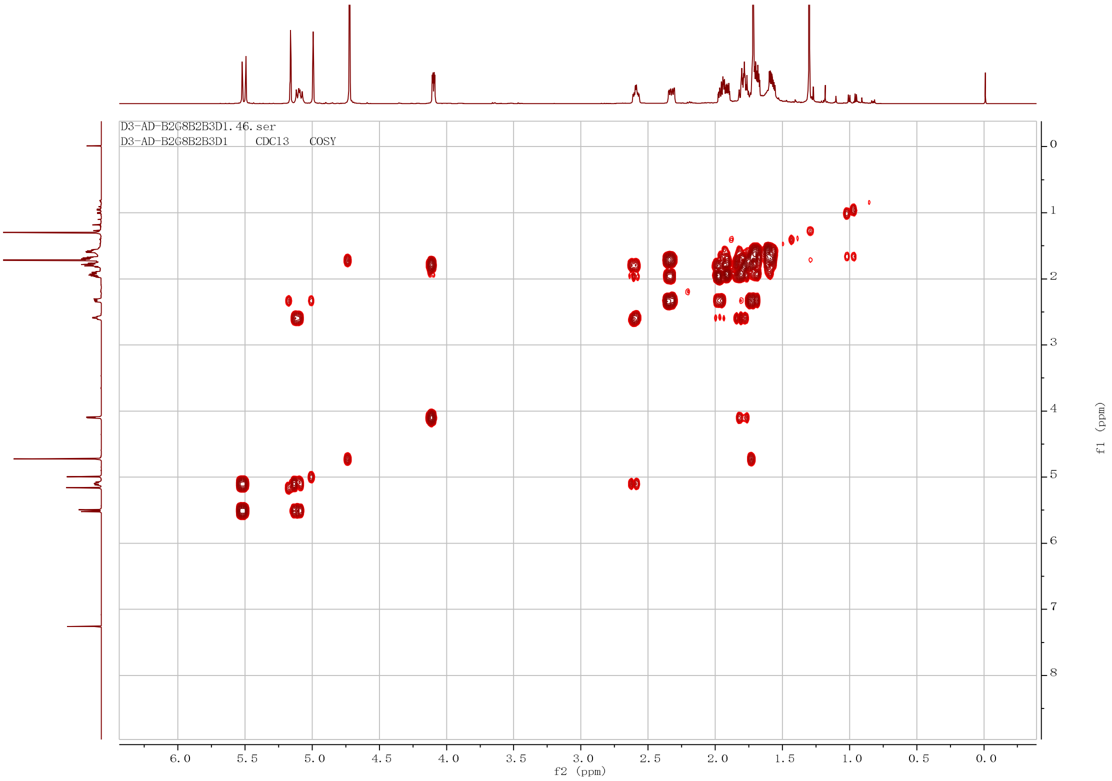

Figure S63 ¹H-¹H COSY spectrum of compound **7**

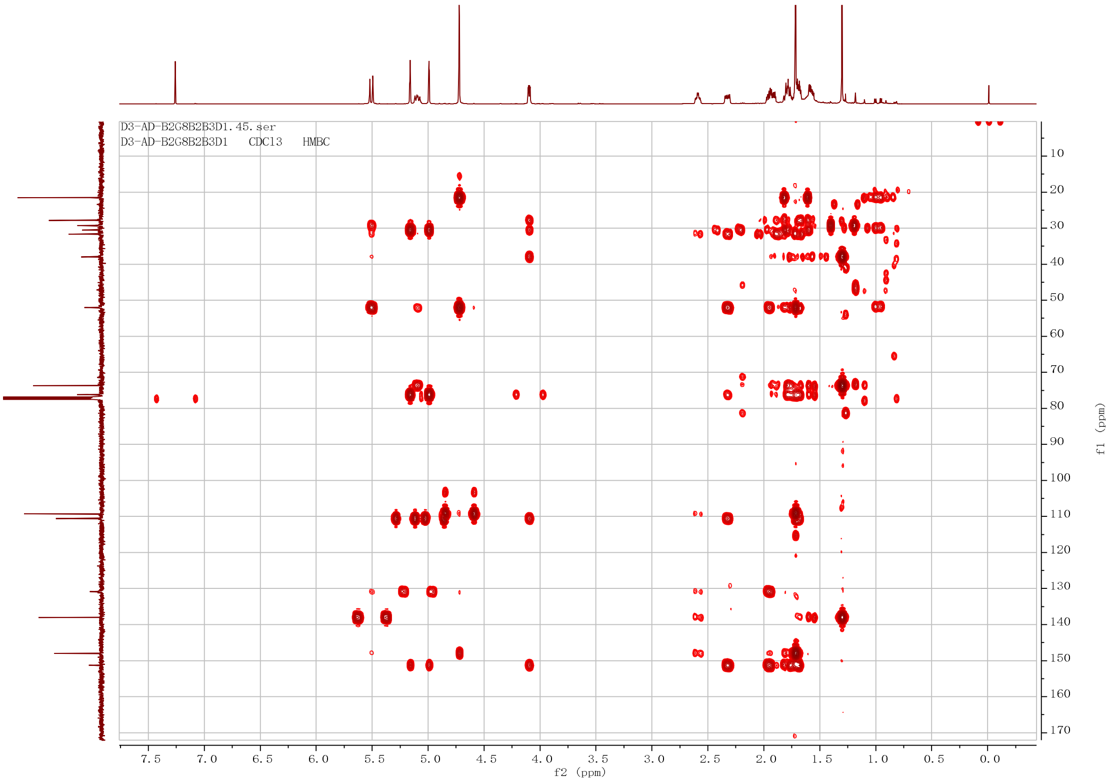

Figure S64 HMBC spectrum of compound **7**

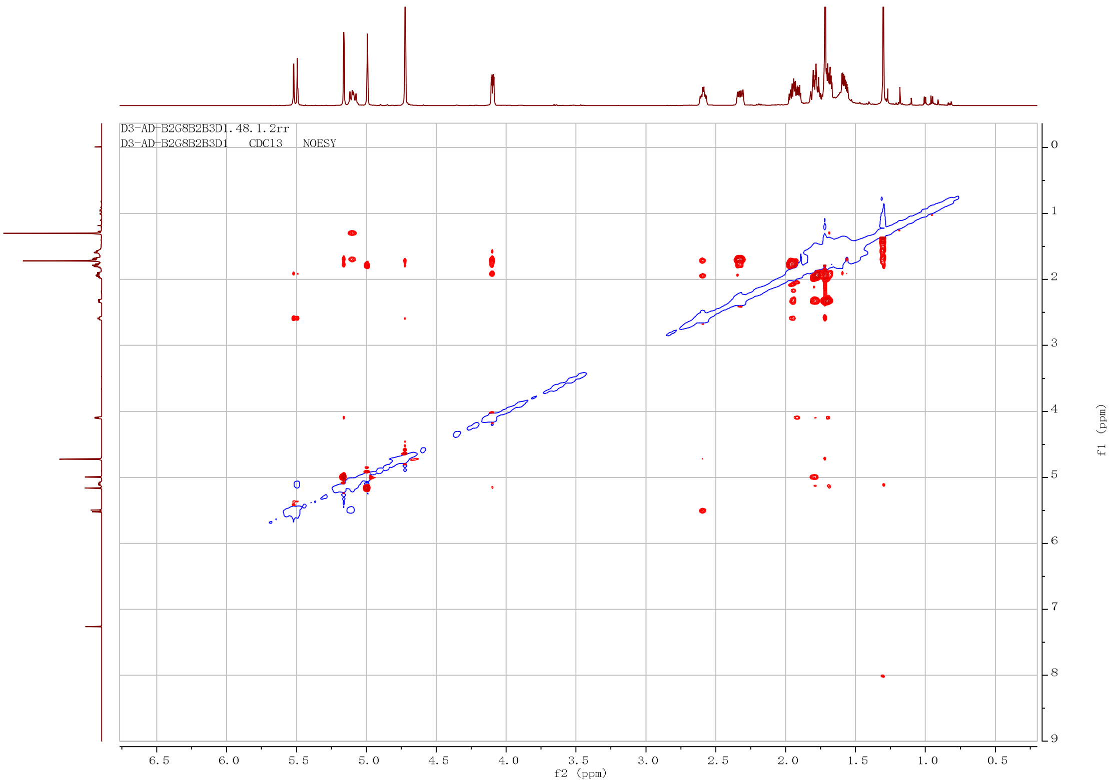

Figure S65 NOESY spectrum of compound **7**

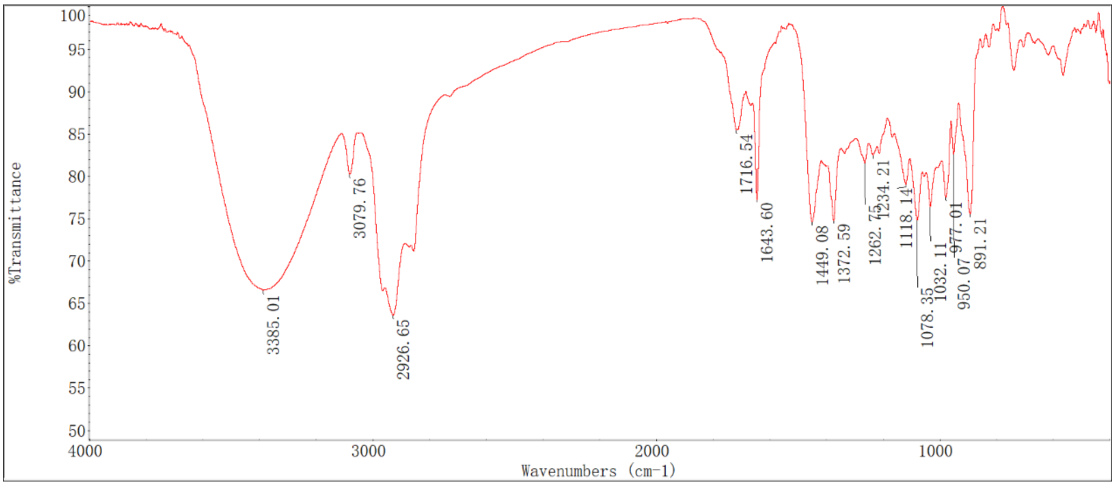

Figure S66 IR spectrum of compound **7**

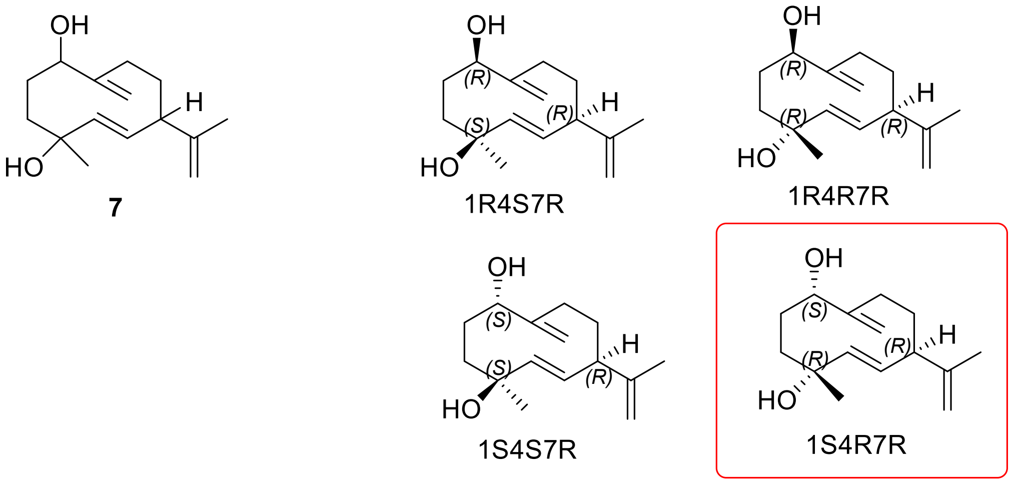

Figure S67 Possible isomers of compound **7**

Functional	Solvent?		Basis Set	
mPW1PW91	PCM		6-311G(d, p)	
	Isomer 1	Isomer 2	Isomer 3	Isomer 4
sDP4+ (H data)	56.38%	3.53%	39.68%	0.41%
sDP4+ (C data)	0.01%	0.00%	99.95%	0.05%
sDP4+ (all data)	0.01%	0.00%	99.99%	0.00%
uDP4+ (H data)	73.73%	0.16%	15.74%	10.38%
uDP4+ (C data)	0.01%	0.00%	99.99%	0.00%
uDP4+ (all data)	0.04%	0.00%	99.96%	0.00%
DP4+ (H data)	86.85%	0.01%	13.05%	0.09%
DP4+ (C data)	0.00%	0.00%	100.00%	0.00%
DP4+ (all data)	0.00%	0.00%	100.00%	0.00%

Figure S68 DP4+ probability statistics of compound **7**

Elemental Composition Report
Tolerance = 5.0 PPM / DBE: min = -1.5, max = 50.0
Element prediction: Off
Number of isotope peaks used for i-FIT = 3
Monoisotopic Mass, Even Electron Ions
22 formula(e) evaluated with 1 results within limits (up to 50 best isotopic matches for each mass)
Elements Used:
C: 0-200 H: 0-60 O: 0-6

Minimum:	80.00				-1.5				
Maximum:	100.00		2.0	5.0	50.0				
Mass	RA	Calc. Mass	mDa	PPM	DBE	i-FIT	Norm	Conf(%)	Formula
219.1745	100.00	219.1749	-0.4	-1.8	4.5	582.0	n/a	n/a	$C_{15}H_{23}O$

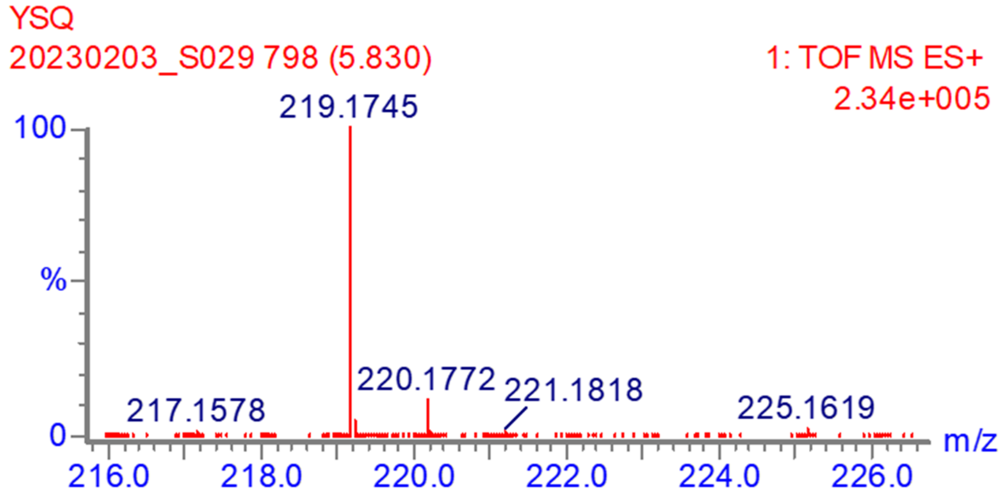

Figure S69 HR-ESIMS spectrum of compound **8**

Figure S70 ^{1}H NMR spectrum of compound **8**

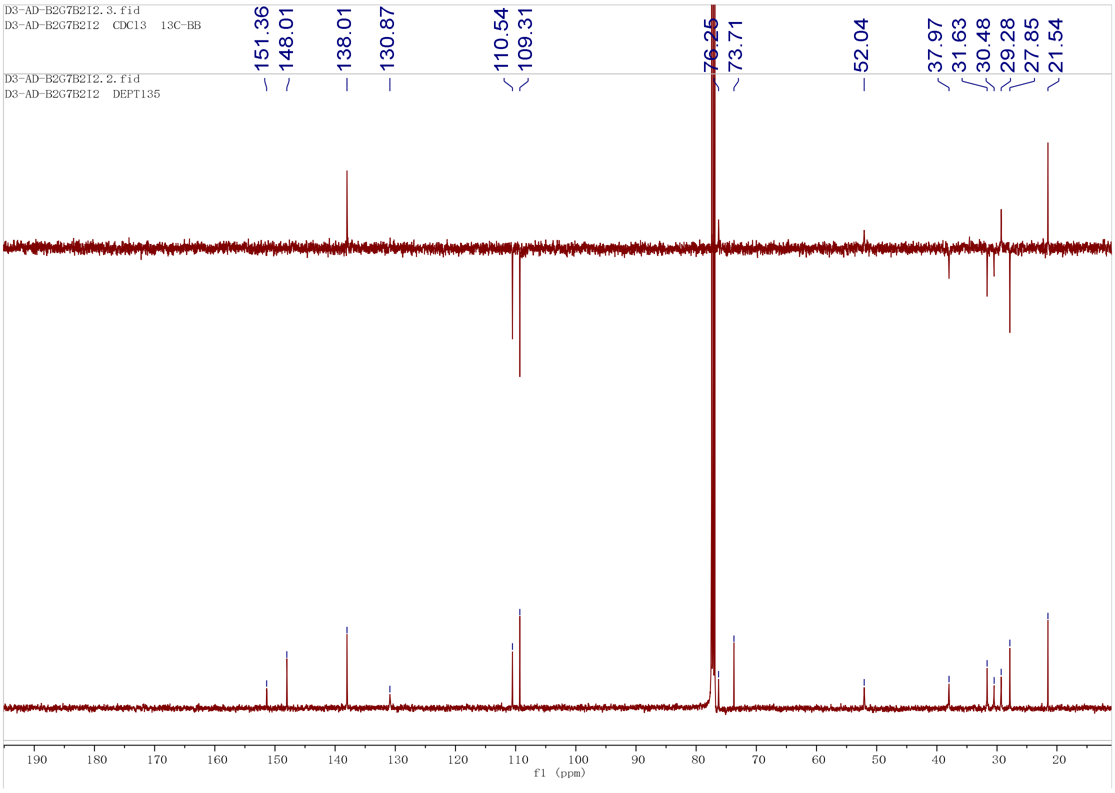

Figure S71 ^{13}C NMR spectrum of compound **8**

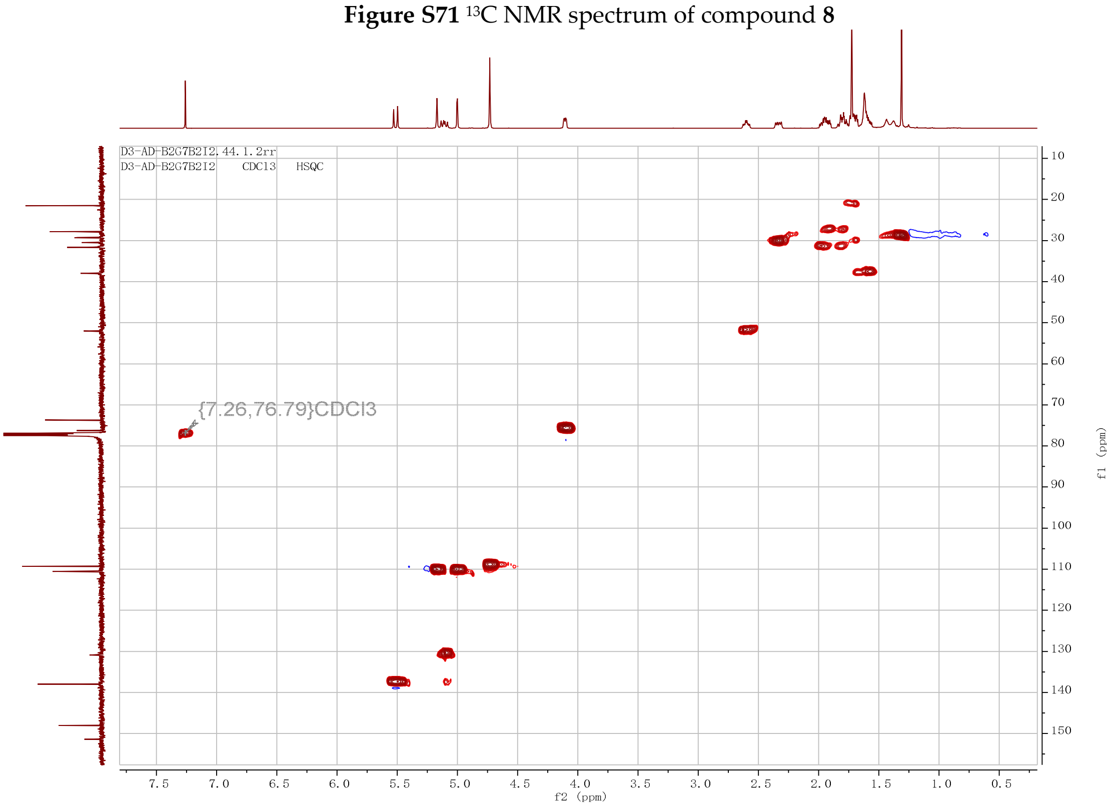

Figure S72 HSQC spectrum of compound **8**

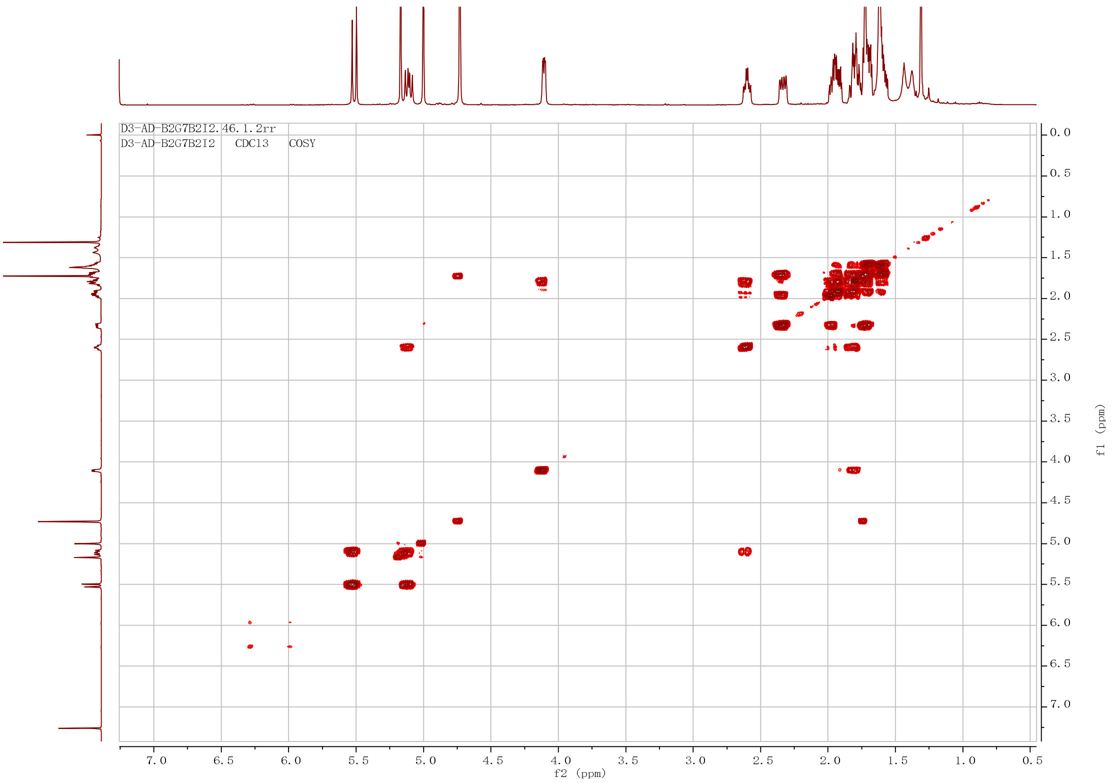

Figure S73 ^{1}H-^{1}H COSY spectrum of compound **8**

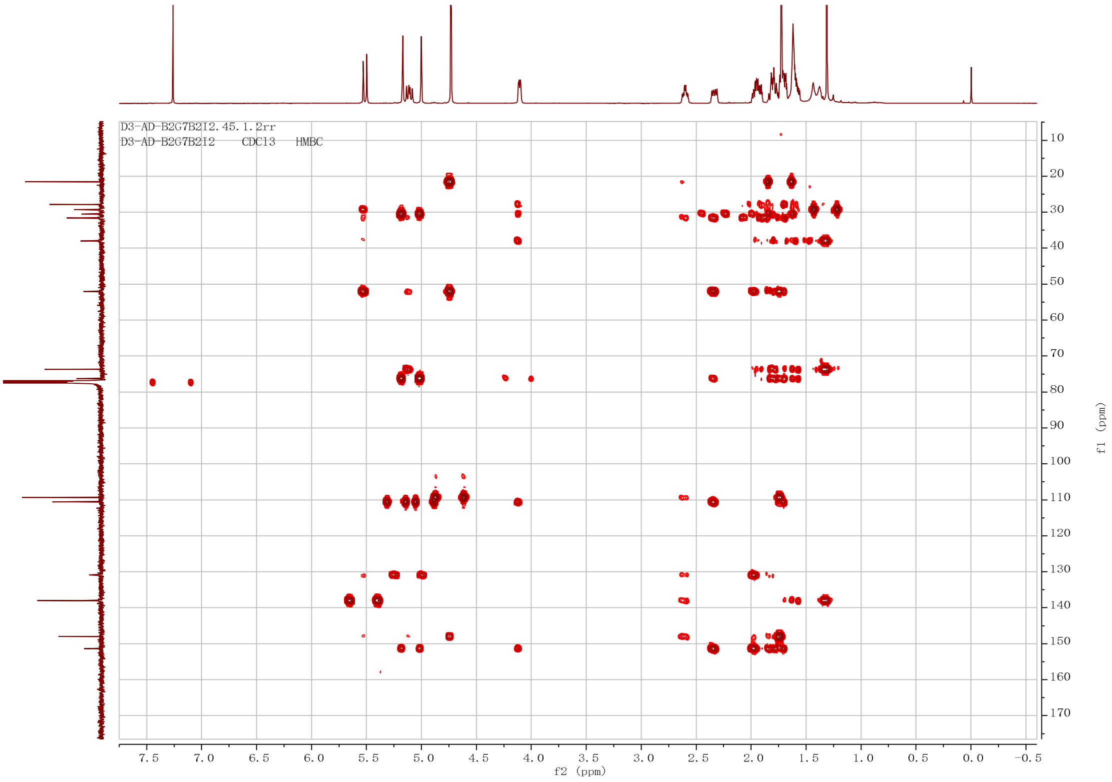

Figure S74 HMBC spectrum of compound **8**

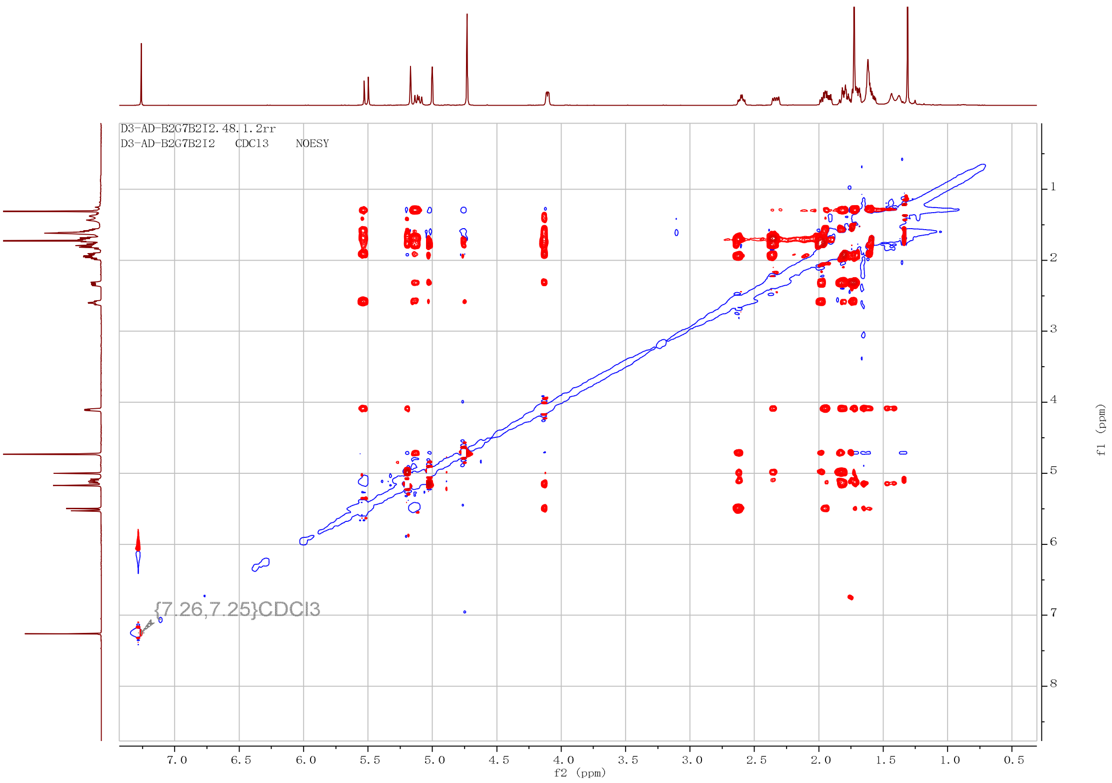

Figure S75 NOESY spectrum of compound **8**

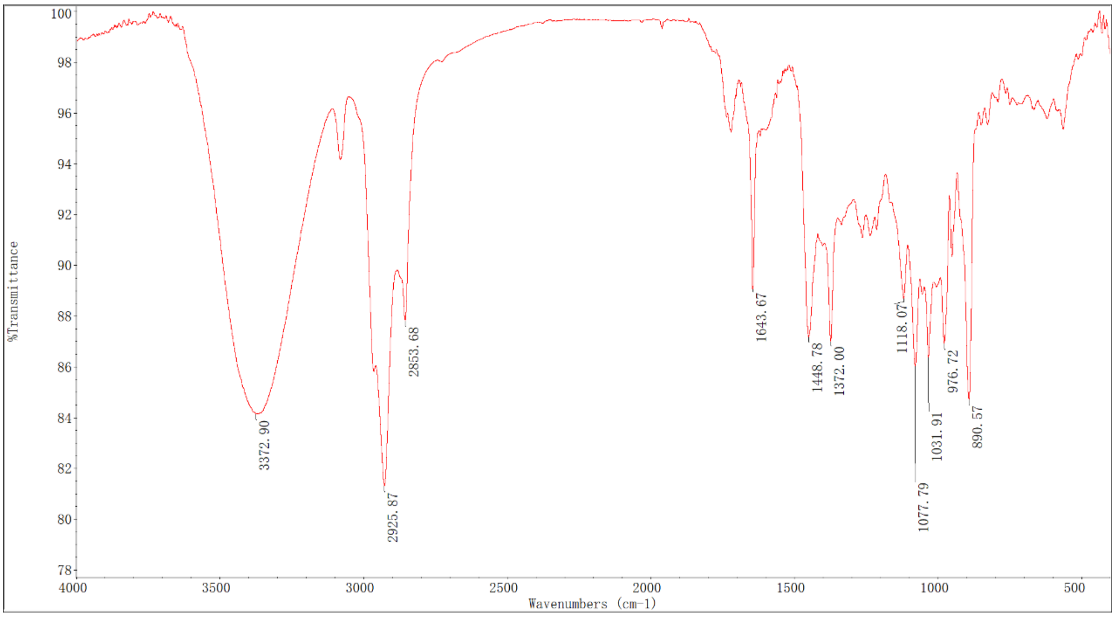

Figure S76 IR spectrum of compound **8**

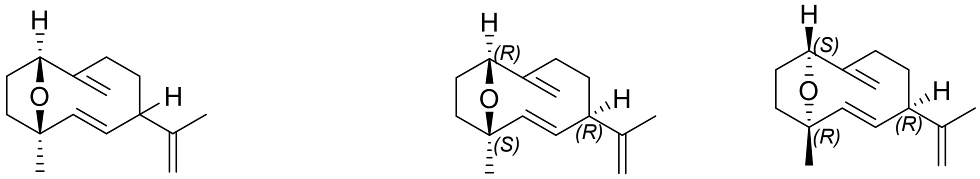

8 1R4S7R 1S4R7R

Figure S77 Possible isomers of compound **8**

Functional	Solvent?	Basis Set	
mPW1PW91	PCM	6-311G(d, p)	

	Isomer 1	Isomer 2	Isomer 3	Isome
sDP4+ (H data)	99.96%	0.04%	–	–
sDP4+ (C data)	98.89%	1.11%	–	–
sDP4+ (all data)	100.00%	0.00%	–	–
uDP4+ (H data)	96.93%	3.07%	–	–
uDP4+ (C data)	58.19%	41.81%	–	–
uDP4+ (all data)	97.77%	2.23%	–	–
DP4+ (H data)	100.00%	0.00%	–	–
DP4+ (C data)	99.20%	0.80%	–	–
DP4+ (all data)	100.00%	0.00%	–	–

Figure S78 DP4+ probability statistics of compound **8**

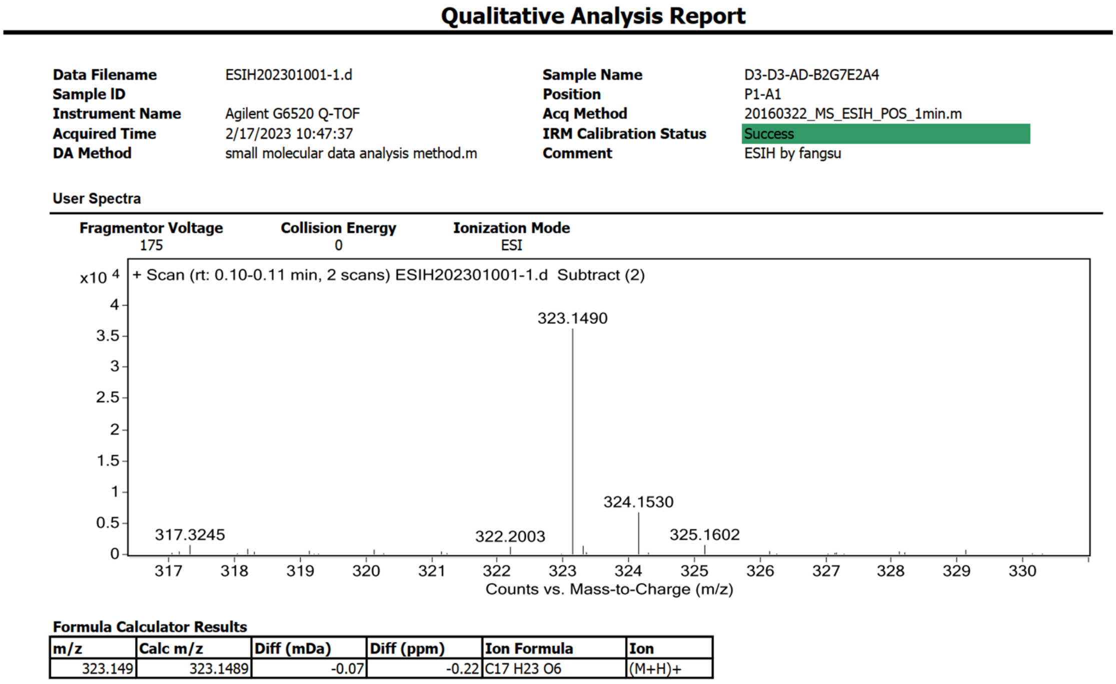

Figure S79 HR-ESIMS spectrum of compound **9**

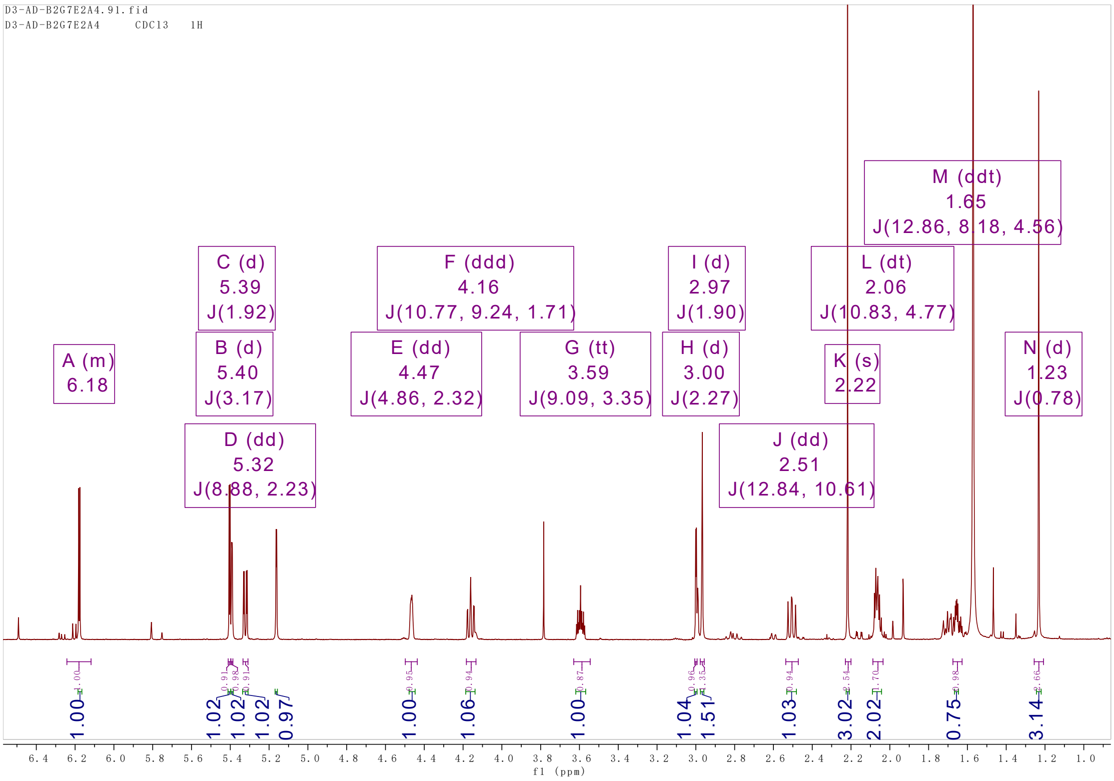

Figure S80 ^{1}H NMR spectrum of compound **9**

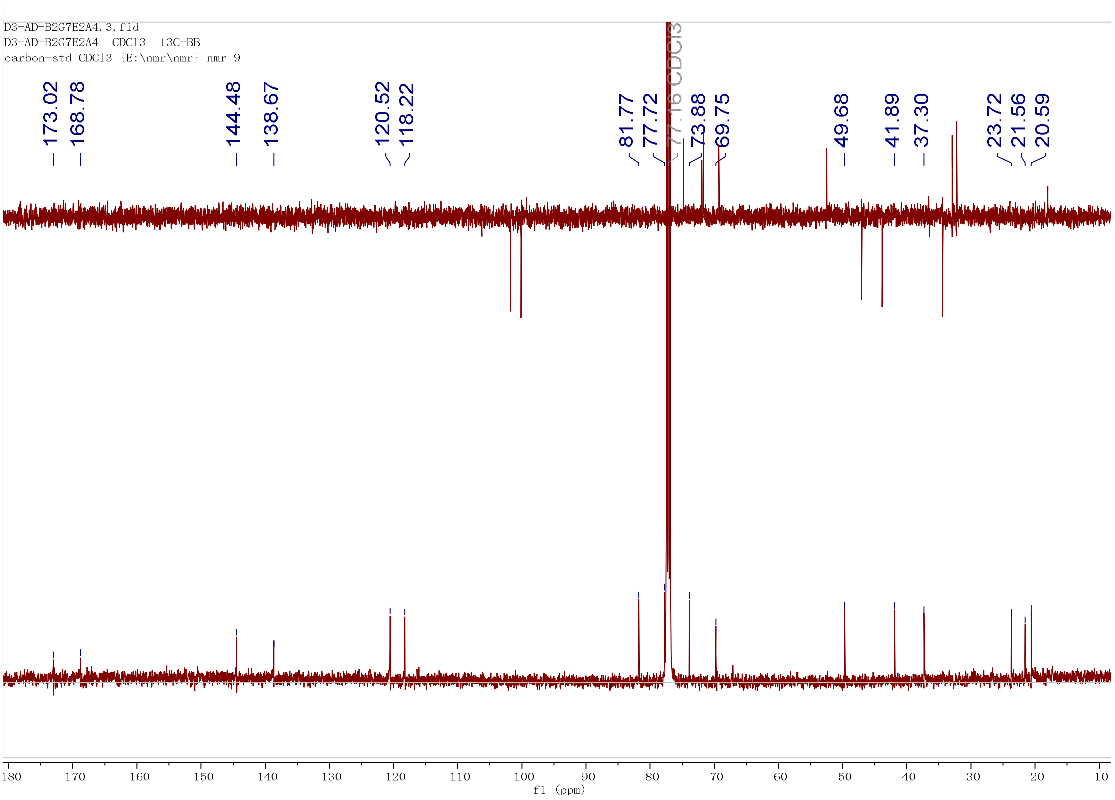

Figure S81 ^{13}C NMR spectrum of compound **9**

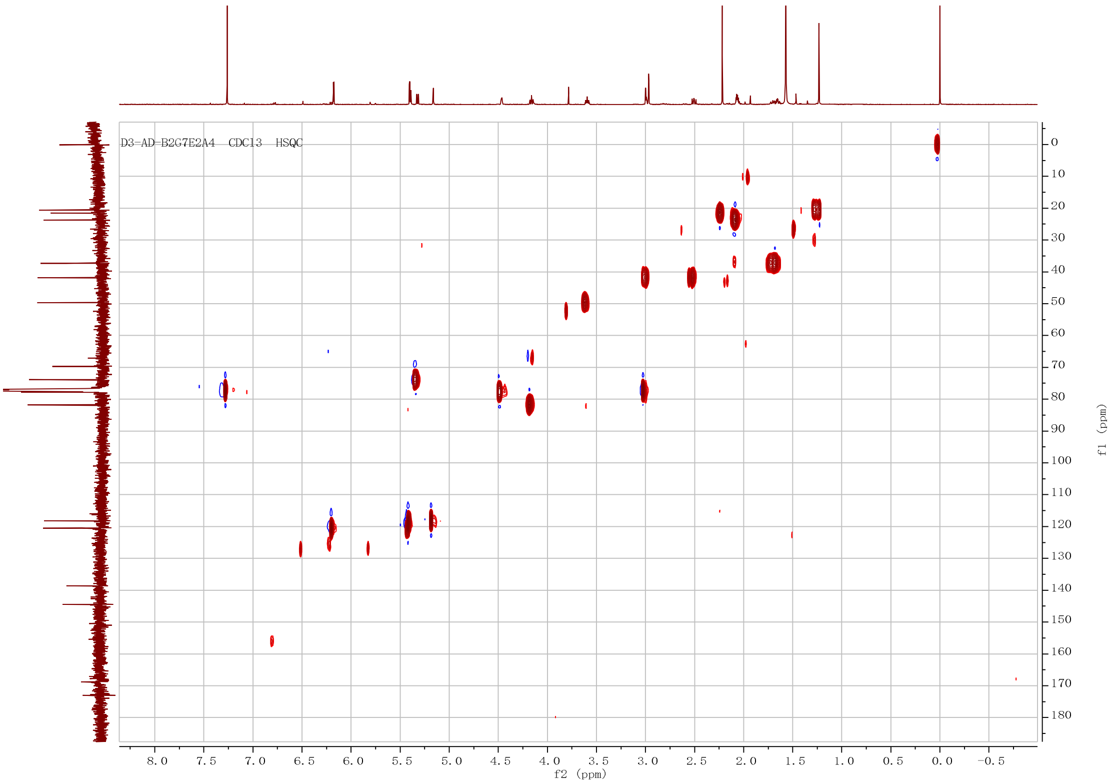

Figure S82 HSQC spectrum of compound **9**

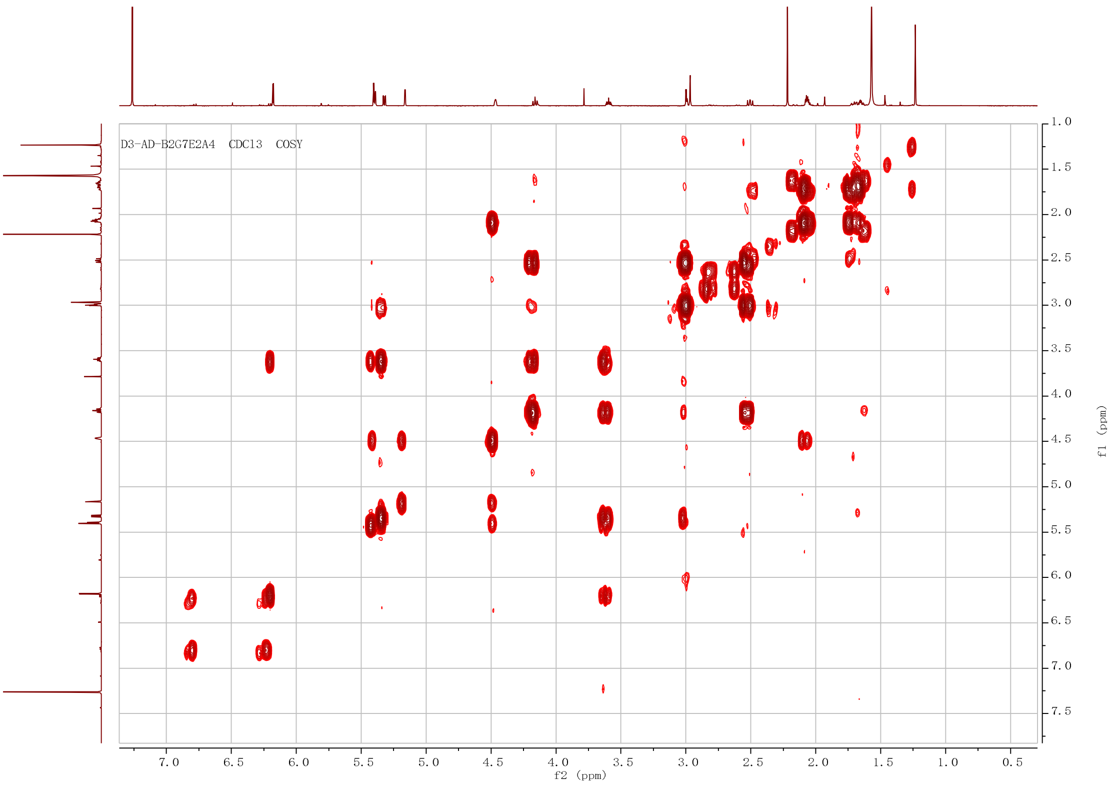

Figure S83 ^{1}H-^{1}H COSY spectrum of compound **9**

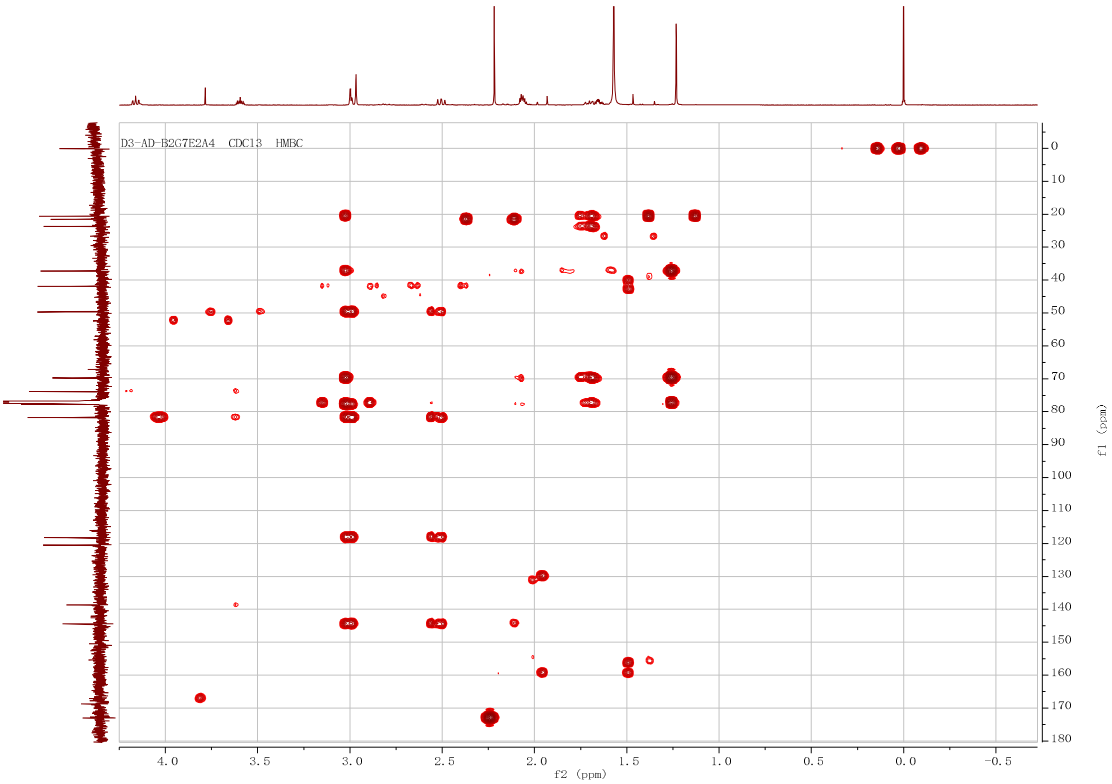

Figure S84 HMBC spectrum of compound **9**

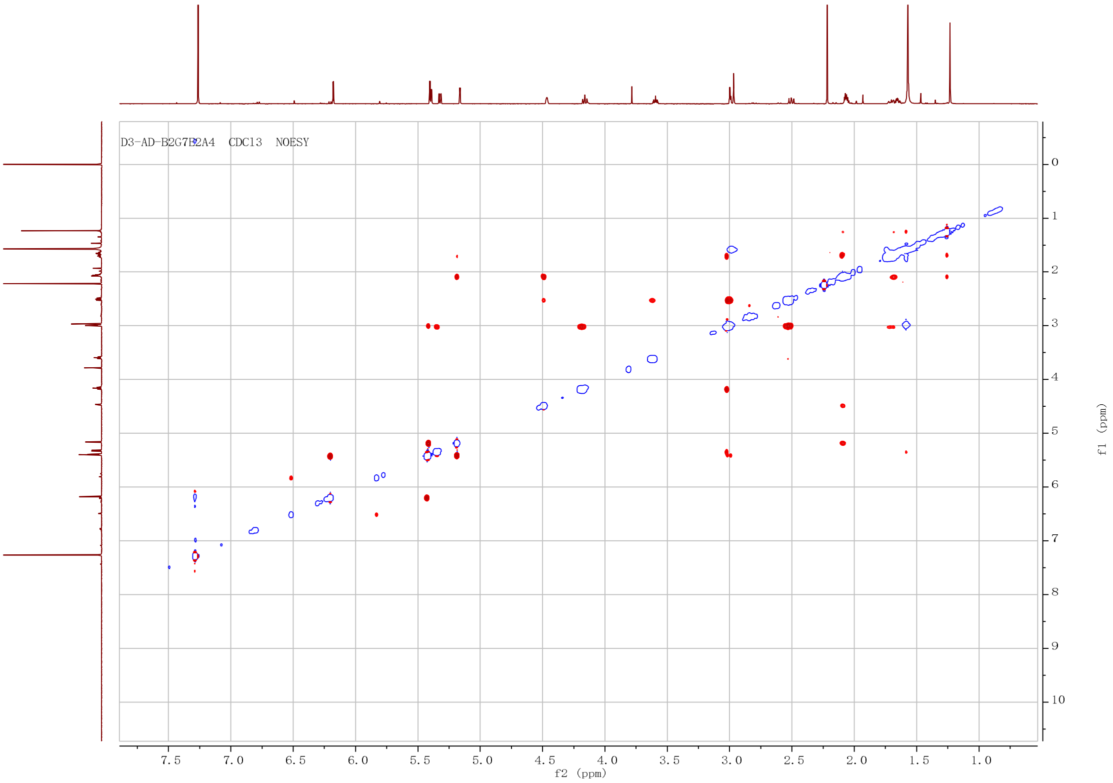

Figure S85 NOESY spectrum of compound **9**

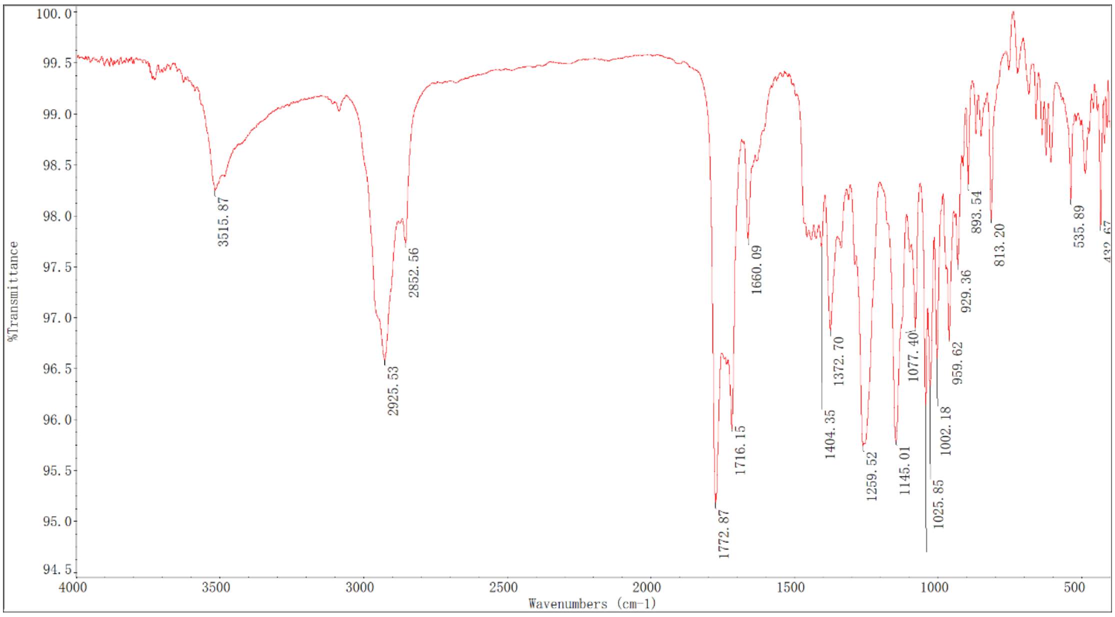

Figure S86 IR spectrum of compound **9**

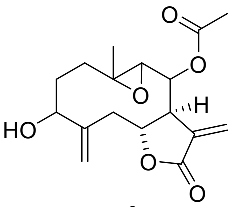

9

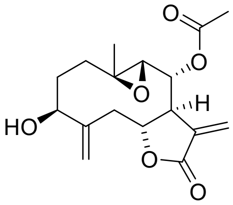

1

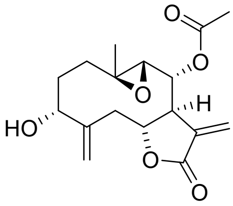

2

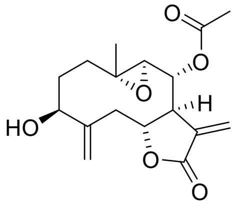

3

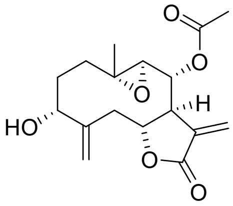

4

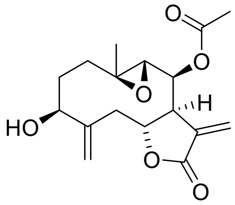

5

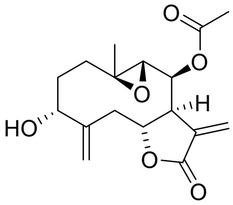

6

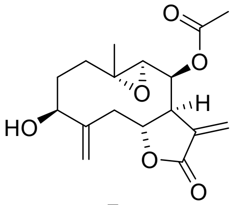

7

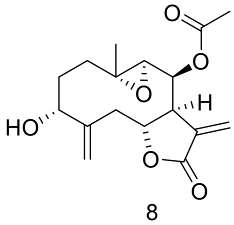

8

Figure S87 Possible isomers of compound **9**

Functional	Solvent?	Basis Set	Type of Data
mPW1PW91	PCM	6-311G(d,p)	Shielding Tensors

	Isomer 1	Isomer 2	Isomer 3	Isomer 4	Isomer 5	Isomer 6	Isomer 7	Isomer 8
sDP4+ (H data)	0.02%	16.99%	0.00%	0.01%	0.00%	82.37%	0.57%	0.04%
sDP4+ (C data)	0.07%	44.23%	0.02%	6.19%	0.11%	0.01%	49.37%	0.00%
sDP4+ (all data)	0.00%	96.29%	0.00%	0.01%	0.00%	0.10%	3.60%	0.00%
uDP4+ (H data)	0.05%	96.23%	0.00%	0.00%	0.00%	3.58%	0.13%	0.02%
uDP4+ (C data)	1.04%	9.56%	0.31%	2.18%	19.70%	38.83%	28.27%	0.11%
uDP4+ (all data)	0.00%	86.59%	0.00%	0.00%	0.00%	13.07%	0.34%	0.00%
DP4+ (H data)	0.00%	84.73%	0.00%	0.00%	0.00%	15.26%	0.00%	0.00%
DP4+ (C data)	0.00%	23.05%	0.00%	0.74%	0.11%	0.02%	76.07%	0.00%
DP4+ (all data)	0.00%	99.97%	0.00%	0.00%	0.00%	0.02%	0.01%	0.00%

Figure S88 DP4+ probability statistics of compound **9**
